# The default network of the human brain is associated with perceived social isolation

**DOI:** 10.1038/s41467-020-20039-w

**Published:** 2020-12-15

**Authors:** R. Nathan Spreng, Emile Dimas, Laetitia Mwilambwe-Tshilobo, Alain Dagher, Philipp Koellinger, Gideon Nave, Anthony Ong, Julius M. Kernbach, Thomas V. Wiecki, Tian Ge, Yue Li, Avram J. Holmes, B. T. Thomas Yeo, Gary R. Turner, Robin I. M. Dunbar, Danilo Bzdok

**Affiliations:** 1 Laboratory of Brain and Cognition, Montreal Neurological Institute, Department of Neurology and Neurosurgery, Faculty of Medicine, McGill University, Montreal, QC Canada; 2 Departments of Psychiatry and Psychology, McGill University, Montreal, QC Canada; 3 Douglas Mental Health University Institute, Verdun, QC HRH 1R3 Canada; 4 McConnell Brain Imaging Centre, Montreal Neurological Institute (MNI), McGill University, Montreal, QC Canada; 5 Department of Biomedical Engineering, Faculty of Medicine, McGill University, Montreal, QC Canada; 6 School of Business and Economics, Vrije Universiteit Amsterdam, Amsterdam, The Netherlands; 7 Marketing Department, the Wharton School, University of Pennsylvania, Pennsylvania, PA USA; 8 Department of Human Development, Cornell University, Ithaca, NY USA; 9 Division of Geriatrics and Palliative Medicine, Weill Cornell Medical College, New York, NY USA; 10 Department of Neurosurgery, Neurosurgical Artificial Intelligence Laboratory Aachen (NAILA), RWTH Aachen University Hospital, Aachen, Germany; 11 Quantopian Inc., Boston, MA USA; 12 Psychiatric and Neurodevelopmental Genetics Unit, Center for Genomic Medicine, Massachusetts General Hospital, Boston, MA 02114 USA; 13 Department of Psychiatry, Massachusetts General Hospital, Harvard Medical School, Boston, MA 02114 USA; 14 Athinoula A. Martinos Center for Biomedical Imaging, Massachusetts General Hospital, Charlestown, MA 02129 USA; 15 Stanley Center for Psychiatric Research, Broad Institute of MIT and Harvard, Cambridge, MA 02138 USA; 16 School of Computer Science, McGill University, Montreal, QC Canada; 17 Departments of Psychology and Psychiatry, Yale University, New Haven, CA 06520 USA; 18 Department of Electrical & Computer Engineering, Centre for Sleep & Cognition, Clinical Imaging Research Centre, N.1 Institute for Health, National University of Singapore, Singapore, Singapore; 19 Department of Psychology, York University, Toronto, ON Canada; 20 Department of Experimental Psychology, University of Oxford, Oxford, UK; 21 Mila - Quebec Artificial Intelligence Institute, Montreal, QC Canada

**Keywords:** Neuroscience, Health care

## Abstract

Humans survive and thrive through social exchange. Yet, social dependency also comes at a cost. Perceived social isolation, or loneliness, affects physical and mental health, cognitive performance, overall life expectancy, and increases vulnerability to Alzheimer’s disease-related dementias. Despite severe consequences on behavior and health, the neural basis of loneliness remains elusive. Using the UK Biobank population imaging-genetics cohort (*n* = ~40,000, aged 40–69 years when recruited, mean age = 54.9), we test for signatures of loneliness in grey matter morphology, intrinsic functional coupling, and fiber tract microstructure. The loneliness-linked neurobiological profiles converge on a collection of brain regions known as the ‘default network’. This higher associative network shows more consistent loneliness associations in grey matter volume than other cortical brain networks. Lonely individuals display stronger functional communication in the default network, and greater microstructural integrity of its fornix pathway. The findings fit with the possibility that the up-regulation of these neural circuits supports mentalizing, reminiscence and imagination to fill the social void.

## Abstract (web summary)

Here, using pattern-learning analyses of structural, functional, and diffusion brain scans in ~40,000 UK Biobank participants, the authors provide population-scale evidence that the default network is associated with perceived social isolation.

## Introduction

Human evolution has been shaped by selection pressures towards enhanced inter-individual cooperation^1,2^. Social interactions are crucial for survival, and fulfillment^3^. Our species’ extraordinary reliance on other individuals has led to the characterization of humans as the “ultra-social animal”^2^. Consequently, the absence of sufficient social engagement can impose substantial physical and psychological costs. A key concern is the experience of ‘loneliness’: the subjective perception of social isolation, or the discrepancy between one’s desired and perceived levels of social connection^4,5^. In the present study, we focus on the time-enduring, rather than momentary, nature of this negative sense of an unmet social need, which we henceforth refer to as ‘trait loneliness’. This concept is distinct from the amount of time spent alone^6^ or the frequency of social contact^4^. One may have few social contacts yet not feel lonely, and vice versa. While there is growing evidence that social connectedness may be associated with brain structure and function^7–9^ (and see Bzdok and Dunbar^10^ for a review), in the current report we directly investigate the neural correlates linked to trait loneliness, that is, the negative subjective experience of social isolation.

Loneliness is estimated to affect 10–20% of adults who lack companionship, consider themselves left out or isolated from others^11^. The health burden of loneliness is pervasive. Loneliness is closely related to morbidity, hypertension, and immune system dysfunction^12,13^ as well as increasing risk for suicide^11,14^. A sense of loneliness has also been associated with health risks that are equivalent to or exceed that of obesity or smoking 15 cigarettes daily^15^. Lonely individuals typically have poorer mental health, higher susceptibility to major psychiatric disorders^11^ and cognitive decline^16,17^, as well as greater neuropathological load with an increased risk of dementia^18–20^. Lonely older adults are 1.64 times more likely to develop clinical dementia than persons who do not self-report as lonely, after accounting for various factors including anxiety and depression^17^.

Given its central role in everyday life, loneliness is likely associated with specific burdens on the brain. Most salient manifestations, we argue, should be expected in brain regions that underwent evolutionary expansion in response to species-specific selection pressures for sociality^1,21^. However, animal studies have so far emphasized differences in subcortical reward systems associated with social isolation^22^. Human studies of loneliness have also observed dampened reward signaling in mesolimbic systems to social cues^23,24^. Research in humans is commonly grounded in the perceptual and attentional sequelae of loneliness. Lonely humans show greater vigilance for, and more rapid detection of, negative social information^22^. Its cognitive and affective characteristics also include heightened emotional reactivity to social stimuli, often in the context of reduced cognitive control^25,26^. Accordingly, brain differences related to the experience of loneliness have been reported in visual cortices, as well as visual attention networks, limbic structures, and prefrontal cortex^27–31^.

Social exchange among humans also encompasses more advanced neurocognitive processes necessary to contemplate one’s thoughts, as well as beliefs and intentions of a social agent. These more complex capacities are necessary for social abilities, such as imagining another’s perspective to hypothesize about social events and interaction partners. Higher-order social abilities are preferentially associated with a collection of brain regions including medial prefrontal and medial temporal lobes, the temporoparietal junction and posteromedial parietal cortex. Collectively, these regions are thought to form a core of the human ‘social brain’^32–34^. These higher-level social brain regions appear to be neglected in the few existing human neuroscience studies on loneliness that highlight lower visual, affective, and attentional circuits. This circumstance is at odds with the common view that recently evolved association cortices are intimately related to reflecting social relationships; and should therefore play a key role in the experience of trait loneliness.

We therefore conducted a systematic assessment of how trait loneliness is manifested in the human brain. Some research has hinted at the existence of a “lonely brain”. Existing studies, however, have relied on a single modality of brain imaging (e.g. structural, functional, or diffusion magnetic resonance imaging [MRI]) and in-laboratory recruitment with limited sample sizes. Here we report the findings of a multi-modal population neuroscience investigation to characterize the structural and functional features of the “lonely brain” in concert. In the UK Biobank imaging-genetics cohort (*n* = ~40,000), we probed gray matter morphology, intrinsic functional connectivity, and white matter tract microstructure to identify a neural signature that differentiates lonely from non-lonely individuals. Further, the present work follows our recent report of sex differentiation in brain volume linked to the regularity of social contact^35^. Building on these recent observations, we also investigated putative sex-specific associations in the context of loneliness across these three assays of brain structure and function.

## Results

Our investigation centered on the binary classification measure of loneliness collected as part of the UK Biobank initiative (data field 2020, “Do you often feel lonely”). 13.1% of participants included in our sample responded ‘yes’ to this questionnaire item (men 38.63%, women 61.37%), consistent with prevalence estimates for loneliness reported in other European population cohort studies^11^. Demographic characteristics for lonely versus non-lonely individuals are provided in Supplementary Table 1. As a preparatory check, we ascertained the biological meaningfulness of trait loneliness as captured in the UK Biobank initiative. Our sample size makes it possible to use LD score regression for direct estimates of shared genetic factors between loneliness and another phenotype of interest (v1.0.0, Bulik-Sullivan et al.^36^). The genome-wide association summary statistics for the loneliness field were obtained from an open UK Biobank resource (https://github.com/Nealelab/UK_Biobank_GWAS#imputed-v3-phenotypes). The genetic correlations between loneliness and the full collection of 774 available demographic, lifestyle, and disease phenotypes were then computed using HapMap3 single-nucleotide polymorphisms (SNPs) from the LDHUB platform (http://ldsc.broadinstitute.org/ldhub/). After Bonferroni’s correction for multiple comparisons, 264 genetically correlated pairs of phenotypes achieved statistical significance at *p* < 0.05. Importantly, our loneliness phenotype shared only moderate genetic overlap with body mass index (Rg = 0.26), level of education (Rg = −0.31), depressive disorder (Rg = 0.61), anxiety (Rg = 0.59), or alcohol intake (Rg = 0.37; see Supplementary Table 2 for full results including confidence intervals and *p*-values). These preliminary findings suggest that binary loneliness reports from UK Biobank participants reflect a heritable biological variation, which is associated with a specific set of driving genetic variants.

**Fig. 1:**
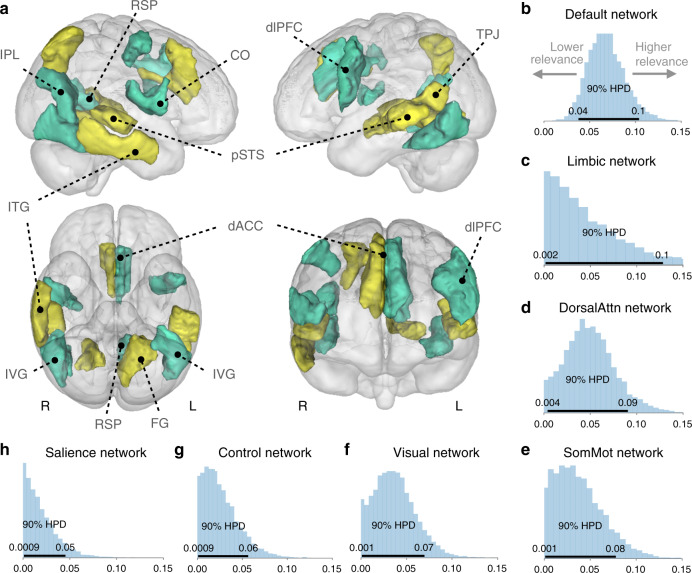
Population associations between loneliness and brain structure. To provide a richer and more precise picture of variation, we purpose-designed a Bayesian hierarchical framework. The fully probabilistic modeling approach allowed jointly estimating varying effects in separate brain regions and spatially distributed networks of constituent brain regions. In rough analogy to ANOVA, the network definitions could be viewed as “factors” and the region definitions could be viewed as continuous factor “levels”. This analysis tactic enabled quantifying the extent to which spatially dispersed regional variation in gray matter volume can be better explained by coherent differences in major brain networks. **a** Contribution of each regional brain volume (thresholded according to 5–95% highest posterior density [HPD], black horizontal line in **b**–**h**) to explain the difference between lonely and non-lonely individuals. Yellow/green = positive/negative volume association. **b**–**h** Shows the degree to which volume variation in each canonical network of regions reliably relates to loneliness. Posterior distributions for the variance parameter (sigma) of each brain network are ordered from the most important (default network) to the least explanatory (salience network). CO central operculum, ITG inferior temporal gyrus, pSTS posterior superior temporal sulcus, TPJ temporoparietal junction, IPL inferior parietal lobe, dACC dorsal anterior cingulate cortex, dlPFC dorsolateral prefrontal cortex, RSP retrosplenial cortex, FG fusiform gyrus, IVG inferior visual gyrus, L/R denotes left/right hemisphere. The shown Bayesian model was fitted once to our whole UKB sample, but brain-loneliness associations held up to cross-validated out-of-sample testing in structural MRI (Supplementary Fig. 11). Source data are provided as a Source Data file.

In the first of the three examined neuroimaging modalities, we explored whether loneliness can be explained by gray matter volume variation in large-scale brain networks across their constituent brain regions. A Bayesian hierarchical framework was devised to associate loneliness with volume variation in all 100 brain regions as a function of seven spatially distributed brain networks based on the Schaefer-Yeo atlas^37^. At the network level, volume variation in the default network dominated the relation to loneliness with the largest share of explained variance (posterior sigma = 0.07; 5–95% highest posterior density [HPD] = 0.04/.10; Fig. 1). The highest relevance of the collection of default network regions in loneliness was followed by overall associations of the limbic network (sigma = 0.06, HPD = 0.01/0.14), dorsal attention network (sigma = 0.05, HPD = 0.01/0.09), somatomotor network (sigma = 0.04, HPD = 0.01/0.08), visual network (sigma = 0.04, HPD = 0.01/0.07), frontoparietal control network (sigma = 0.03, HPD = 0.01/0.06), and, relatively least explanatory, the salience network (sigma = 0.02, HPD = 0.01/0.05). Considering the confidence estimates of overall network relevances (i.e., 5–95% HPD interval of the posterior density of sigma), the default network also prominently featured the most informative (i.e., tightest width) posterior distribution among all seven network variance components. This finding suggests that distributed patterns of gray matter variability across all examined canonical networks were linked to loneliness. However, regions collectively composing the default network showed consistently strongest volume deviations in lonely participants; some of which based on positive, some with negative regional associations with that trait (Fig. 1).

We subsequently examined the association between loneliness and regional brain structure in our population cohort. The Bayesian hierarchical approach was used to focus on variation in gray matter volume in the 100 individual atlas regions (Fig. 1). Positive region-level volume associations with loneliness emerged in the posterior superior temporal sulcus on the left (posterior mean = 0.14, 5–95% HPD = 0.01/0.27) and right (mean = 0.27, HPD = 0.10/0.44), the left temporoparietal junction (mean = 0.17, HPD = 0.05/0.28), as well as the left fusiform gyrus (posterior mean = 0.13, HPD = 0.01/0.26), right inferior temporal gyrus (mean = 0.31, HPD = 0.16/0.46), right posterior parietal lobe (mean = 0.15, HPD = 0.03/0.26), and right dorsal anterior cingulate cortex (mean = 0.14, HPD = 0.01/0.28). In contrast, the left dorsal anterior cingulate cortex showed a negative region-level volume association (mean = −0.14, HPD = −0.27/−0.01) in addition to the dorsolateral prefrontal cortex on the left (mean = −0.12, HPD = −0.24/−0.01) and right (mean = −0.12, HPD = −0.22/−0.02), the right central operculum (mean = −0.16, HPD = −0.29/−0.05) and the right inferior parietal lobule (mean = −0.27, HPD = −0.42/−0.14), left retrosplenial cortex (mean = −0.19, HPD = −0.30/−0.08) as well as the inferior visual cortex on the left (mean = −0.18, HPD = −0.33/−0.01) and right (mean = −0.19, HPD = −0.34/−0.03). Thus, regarding the level of individual brain regions, lonely UK Biobank participants showed idiosyncrasies in gray matter morphology distributed across the brain.

We next investigated the impact of biological sex on the association between gray matter volumes and trait loneliness. As observed in the main findings, the default network showed the most reliable relationship with loneliness. This association was consistent across men (posterior sigma = 0.08, 5–95% HPD = 0.05/0.13) and women (sigma = 0.08, HPD = 0.03/0.13; Supplementary Table 3). However, reliable sex differences in the relationship between loneliness and brain structure were observed in the somatomotor network: men showed stronger volume associations (sigma = 0.10, HPD = 0.02/0.16) than women (sigma = 0.03, HPD = 0.01/0.06; Supplementary Fig. 1). At the single-region level, the right dorsomedial prefrontal cortex showed a negative volume association in men (mean = −0.10, HPD = −0.22/−0.01), in contrast to that of women (mean = 0.04, HPD = −0.04/0.13). We thus identified robust gray matter differences between lonely men and women that were reliable, both in individual brain regions and integrative brain networks.

**Fig. 2:**
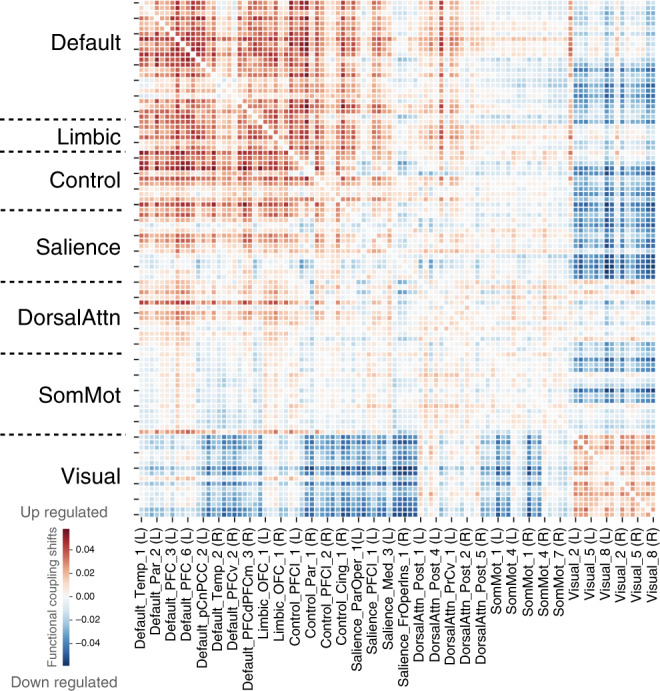
Statistically strongest population mode of functional connectivity deviations related to loneliness. Loneliness is linked to functional coupling shifts (on *z*-score scale) with increased intra-network connectivity especially in the default network and decreased inter-network connectivity of the visual cortex with various other neural systems, including the default network. The best partial least-squares mode was computed and found statistically significant at *p* < 0.05 according to non-parametric permutation testing (false discovery rate, one-sided test, no additional adjustment for multiple comparisons). L/R denotes left/right hemisphere. The shown machine-learning model was fitted once to our whole UKB sample, but brain-loneliness associations held up to cross-validated out-of-sample testing in functional MRI (Supplementary Fig. 11). Source data are provided as a Source Data file.

In a second set of analyses, we examined the resting-state functional connectivity between the 100 regions from the Schaefer-Yeo atlas (as above) with their relation to loneliness. This in vivo measure of cortex-wide intrinsic functional couplings in each participant was submitted to a multivariate pattern-recognition algorithm (Fig. 2). Partial least squares was used to identify the single most coherent pattern of deviation within the functional connectome that characterize lonely individuals. In the statistically strongest population mode of functional covariation (*p* < 0.05 based on non-parametric permutation test), lonely individuals demonstrated a greater magnitude of within-network coupling for the default network. That is, the most prominent within-network alterations emerged for regions communicating with other regions within the default network. Additionally, trait loneliness was also partly explained by up-regulated between-network coupling of the default network with the limbic, dorsal attention, and somatomotor networks. Those canonical networks are more functionally anti-correlated in non-lonely participants. As the second most prominent finding in brain function, lonely individuals showed a pattern of greater within-network coupling for the visual network. This lower-sensory network also showed weaker between-network coupling with several other neural systems of the brain.

We again explored potential sex differentiation by repeating these pattern-learning analyses in male and female cohorts separately. While the functional coupling patterns observed in the whole group analysis were preserved for the sex-based analyses (Supplementary Fig. 2), the connectivity pattern linked to loneliness appeared to be more strongly expressed in men than women. Taken together, the results on loneliness and functional brain connectivity suggest a shift of balance between networks situated lower (sensory) versus higher (integrative) systems in the neural processing hierarchy, and this shift appears greater for men.

**Fig. 3:**
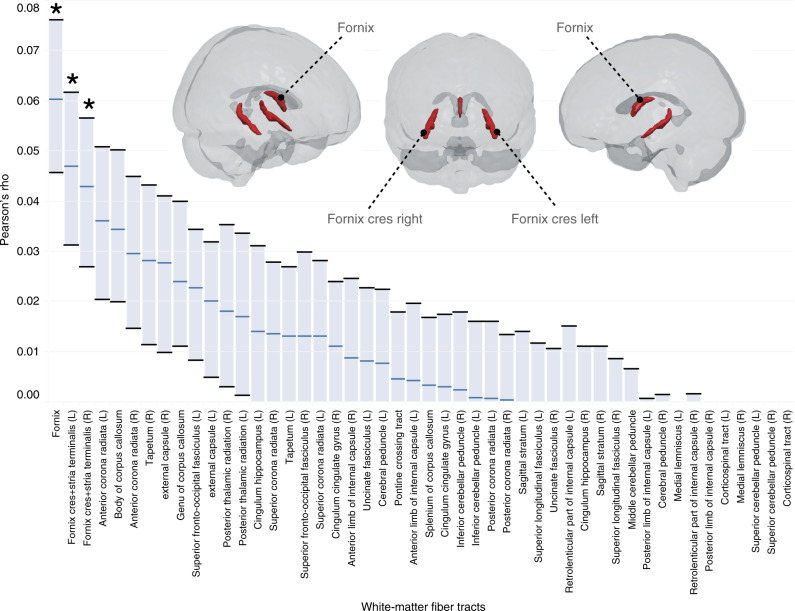
Fornix fibers emerging from the hippocampus are linked to loneliness. Among 48 examined major white matter tracts (ordered from strongest to weakest association), the top three fiber bundles with relation to loneliness carried fornix fibers (red) towards higher integrative brain areas including regions of the default network (tracts statistically significant at *p* < 0.001, after Bonferroni’s family-wise error correction, show asterisks). In addition to the obtained Pearson’s correlation rho between loneliness and tract microstructure (center of barplot), population intervals of 5–95% uncertainty (error bars) estimated the expected variation of this correlation effect size based on 100 bootstrap resampling iterations (cf. Supplementary Tables 4–6). L/R left/right hemisphere. The shown combination of Pearson’s correlation and bootstrapping was carried out in our whole UKB sample, but brain-loneliness associations held up to cross-validated out-of-sample testing in diffusion MRI (Supplementary Fig. 11). Source data are provided as a Source Data file.

In the third set of analyses, we examined whether loneliness is related to the white matter integrity of major tracts from the widely used John-Hopkins atlas (Fig. 3 and Supplementary Table 4). Among the 48 candidate tracts, the strongest linear associations were found in three tracts that are known to carry fibers of the fornix, which originate from the hippocampus (Pearson’s rho_fornix_ = 0.06, rho_fornix_cres_left_ = 0.05, rho_fornix_cres_right_ = 0.05; all *p* < 0.001 after family-wise error correction with Bonferroni’s method; see Supplementary Tables 2–4 for full details). Additional tests for non-linear associations (Spearman’s rho) that can detect more complicated patterns in the data further confirmed the fornix tracts to be the top 3 tracts with relation to loneliness.

As we did for the volumetric and functional coupling analyses, we repeated the diffusion analyses separately for males and females. For all three fornix-related fiber bundles, these analyses revealed stronger effect sizes in men (Pearson’s rho_fornix_ = 0.05, rho_fornix_cres_left_ = 0.05, rho_fornix_cres_right_ = 0.03) than in women (Pearson’s rho_fornix_ = 0.03, rho_fornix_cres_left_ = 0.02, rho_fornix_cres_right_ = 0.02). We made similar observations when testing for non-linear associations (Spearman’s rho). As such, the exploration of white matter microstructure revealed that the major output tract of the hippocampus in the limbic medial temporal lobe, channeling information to higher integrative brain regions, has greater microstructural integrity in individuals who report being lonely. These anatomical associations with loneliness were more pronounced in men, compared to women, in our population cohort (see Supplementary Tables 5 and 6 for details including population confidence intervals).

Note that the above findings in each neuroimaging modality were obtained after adjusting for nuisance variation that was related to body mass index, head size, head motion during task-related brain scans, head motion during resting-state fMRI scanning, head position as well as receiver coil in the scanner (x, y, and z), position of scanner table, and data acquisition site (cf. methods). Additionally, the convergent findings from structural, functional, and diffusion imaging modalities held up after accounting for variation in our brain data that could be explained by clinical diagnoses of major depressive disorder (ICD F32*/F33*) and anxiety disorders such as agoraphobia, social phobia, panic disorder, or generalized anxiety disorder (ICD F40*/F41; Supplementary Fig. 3 and Supplementary Tables 7–9). Accounting for trait neuroticism, regular alcohol consumption, education, higher-order age effects, and adiposity also confirmed the strongest gray matter deviation of default network regions in loneliness, heightened functional coupling inside the default network, and fornix fibers remained the strongest associations of white matter tracts with loneliness (Supplementary Figs. 4–8 and Supplementary Tables 10–24). Moreover, we also observed this constellation of findings after accounting for any ethnicity other than white British ancestry (Supplementary Fig. 9 and Supplementary Tables 25–27).

We next examined how the brain and trait loneliness associations for each modality were related across brain-imaging modalities. These linear associations confirm the broad pattern of associations, but also highlight that each modality-specific analysis revealed specific features of brain-loneliness associations (Supplementary Fig. 10). Finally, we computed modality-specific effect sizes as well as derived in-sample model accuracy and out-of-sample prediction performances in structural, functional, and diffusion MRI as additional evidence for the robustness of the observed associations between brain measures and trait loneliness (Supplementary Fig. 11).

## Discussion

The course of primate evolution has been marked by a trajectory towards social coordination and cooperation^1,2^. Increasing sociality has coincided with the emergence of more sophisticated, computationally more powerful, and metabolically more expensive brains^1^. Hence, we expected unmet desires for social interaction in humans (i.e., loneliness) to be associated with a unique neural signature, which implicates evolved higher-associative regions. Gray matter volumes, intrinsic functional connectivity, and white matter tract integrity showed distinctive features in the “lonely brain”. While differences were observed across the brain, three separate windows into the brain using multimodal neuroimaging converged on the default network as the center of the neural expression of loneliness.

Among the scant human neuroscience studies on loneliness, visual, attention, and limbic regions have been repeatedly emphasized^21,22,27^. These neural findings align with the behavioral sequelae of loneliness, which involves an attentional bias for negative social cues in the context of reduced cognitive control^22–24,30^. Consistent with these earlier neuroimaging results, we confirm gray matter deviations related to loneliness in attentional and limbic networks in UK Biobank participants. Further, at the population-level, we have associated loneliness with shifted functional connectivity within the visual sensory cortex and between the visual system and other canonical brain networks, paralleling early task functional MRI experiments^22^. Consistent with this, Layden et al.^30^ recently proposed a neural account of loneliness that included heightened functional connectivity within a cingulo-opercular vigilance network and reduced connectivity between this network and parietal brain regions implicated in control processes. In line with this previous research, we also observed consistent volumetric alterations in right inferior parietal and cingulo-opercular regions as well as in dorsolateral prefrontal cortex. Taken together, our findings support this tentative neural model of loneliness. Brain regions associated with perceptual, attentional, and affective processing of social information were indeed altered by the experience of loneliness.

However, our collective multimodal findings question the completeness of the predominant neural account of loneliness, which places emphasis on altered externally oriented social processing. Across three complementary brain measurements, we have observed the most salient brain manifestations of loneliness in the default network. Against our expectations, cortical volumes in several default network regions were larger for lonely than non-lonely individuals. A similarly unexpected pattern of positive associations was corroborated for both functional connectivity and white matter structure. Functional connectivity shifts within the default network, as well as between the default, limbic, and somatosensory networks, was positively linked to loneliness. In line with these observations, the microstructural integrity of the fornix, a core fiber tract carrying neural signals via axonal connections from the hippocampal subsystem into the cortical default network^38^, was greater in lonely individuals.

The default network is an assembly of higher association areas, which is known to overlap with the human social brain^34,39^. Advanced social abilities of humans likely developed in parallel with the rapid expansion of cortical volume in humans, involving default network regions, as compared to our closest primate ancestors^40^. Numerous studies have now shown that this collection of brain regions, including the medial temporal memory subsystem, is reliably engaged by experimental tasks that require withdrawal from perceptual experiences in the sensory environment to internally constructed mental simulations of physically, temporally, and socially distal events^41^. The default network is well-known to be implicated in mental representations of oneself across time and space, including the reconstruction of one’s personal past, prospecting and planning about an envisioned future, imagination and creative thought as well as simulating thoughts, places, and events^33^. The default network is also recognized for its role in representing other people, including their intentions, identity, and affiliation^33,34,42,43^.

Recent work has demonstrated that lonely individuals show greater distinction between neural representations of oneself from social others within the medial prefrontal cortex, a core node of the default network^44^. This observation was taken to suggest that the subjective experience of social distance is instantiated as greater distance between neural representations of oneself and social others in the default network of the brain^44^. These authors argue that the social brain facilitates navigation of the social world in part through an egocentric proximity mapping, based upon affiliation and closeness, of other people within our social network. Chronic feelings of social disconnection are mirrored by a ‘lonelier’ neural self-representation, which distorts the internal neural representation of others.

Our population-level findings are in line with and extend this previous view, which relates mnemonic as well as social functions of the default network to the subjective experience of loneliness. In the context of previous research, we speculate that in the absence of desired social experiences, lonely individuals may be biased towards internally directed cognitions mediated by default network brain regions^33^. Phenomenologically, this brain-behavior association would manifest as greater attention to one’s inner milieu, and a heightened focus on the self and self-reflective thoughts, which would naturally engage memory-based functions of the default network. These neurocognitive processes include reminiscing, future thinking, imagining or mentally simulating desired social exchanges. Consistent with this idea, persons who face social disconnection are known to more frequently engage in random imagination of social interaction^45^, nostalgic reminiscences^46,47^, hypothetical conversations^48^, and treating pets as if they were human agents^49^.

This link between loneliness and the mnemonic functions of the default network dovetails with our observation of greater microstructure of the fornix fiber bundle in lonely individuals. Direct axonal connections between the limbic system and regions of the higher association cortex are carried by the fornix white matter tract. Specifically, this major output pathway transports signals from the hippocampus to the medial prefrontal cortex of the default network^50^. Inter-individual differences in fornix microstructure were indeed reported to explain variation in episodic detail generation^51^ during both recollection and prospection^52^. Thus, the integrity of this pathway is likely implicated in recapitualization of the past and imagination of the future – all forms of mental simulation that are known to be heightened in lonely individuals^45–49^.

We extend the nascent neuroimaging research on loneliness by directly exploring sex differences in these brain-behavior associations. While one previous neuroimaging study did not find sex differences^31^, our population study uncovered a pattern of sex differentiation indexed by structural, functional, and diffusion imaging markers. The sex-focused analyses closely reflected the findings from the full sample. However, we observed a recurring trend of more prominent associations between loneliness and brain structure and function for men compared to women. This sex idiosyncrasy was particularly apparent in functional connectivity fingerprints and white matter architecture. Loneliness in men was associated with more pronounced within-network coupling of the default network and functional de-coupling of the lower visual-sensory networks, and greater structural integrity of the fornix. We thus report tentative sex-specific patterns in three separate types of brain tissue measures. Stigma may preclude more men from self-labeling as lonely, resulting in a lower reporting threshold for women than men^53^. Future work is necessary to further explore possible sex differentiation in loneliness and its neurocognitive bases.

From a broader perspective, our results also raise new questions regarding the potential neural substrates of loneliness in later life. There is now an abundance of evidence demonstrating that large-scale brain networks, including the default and frontoparietal control networks, display reduced within- and increased between- network connectivity in older adulthood^33,54–56^. The UK Biobank sample examined here represents a mid- to late-adulthood cohort (mean age: 54.9 years, range 40–69 years at enrollment). Thus, our findings of greater integrity within the default network may appear counterintuitive in this late middle-age to older adult cohort. In particular, earlier work reported lower intrinsic functional connectivity of the default network to be associated with loneliness in a cohort of young adults^57^. However, the mid- to late-adulthood sample assessed here possibly included more participants who experienced chronic periods of loneliness than in the young adult study. While the UK Biobank provides only a single brain scanning session for the majority of the participants, chronic loneliness may be associated with greater coupling among default network brain regions, as well as greater between-network connectivity with the frontoparietal control network, and signal a maladaptive shift in the network architecture of the lonely aging brain.

Our population neuroscience investigation capitalized on the rich UK Biobank imaging-genetics resource to systematically chart the brain manifestations linked to loneliness. Three separate windows into the human brain enabled a more integrated understanding of the neural signature linked to perceived social isolation. While some discovered brain substrates are subtle, the brain-loneliness associations held up to cross-validated prediction in unseen participants in all three brain-imaging modalities (Supplementary Fig. 11). As our core conclusion, brain divergence found in lonely, compared to non-lonely, individuals centered on the default network – a collection of brain regions otherwise known to overlap with the ‘social brain’. We speculate that the associations between the default network and loneliness revealed here reflect increased demands on episodic mental simulation of inner social events in the absence of desired social experience in the external world.

## Methods

### Rationale and workflow summary

To extend earlier neuroimaging studies on loneliness, we aimed at a comprehensive multimodal assessment of how loneliness manifests itself in the brain across structural, functional, and diffusion brain scanning. These three views into the neuroscientific phenomenon of trait loneliness however relied on divergent MRI physics, as well as different aspects of sampled tissue biology, and thus resulted in distinct signal properties. For these reasons, we aimed to carefully tailor our analytical approach to each imaging modality.

For the structural brain scans, the single set of gray matter measurements (100 regions) per participant was analyzed by assuming interregional associations, as defined through the hierarchical region-network information in the atlas, which could be naturally integrated into the design of a Bayesian hierarchical model, while the uncertainty in effect sizes can be evaluated by posterior parameter distributions. The time series derived from functional brain scans were first summarized into a connectivity matrix of interregional coupling estimates (4950 connectivity links) specific for each participant. These functional coupling matrices were then analyzed by partial least squares (PLS), which is known to deal well with strongly correlated high-dimensional data, while the uncertainty in effect sizes was evaluated based on empirical permutation distributions. For these cross-connectivity matrices, application of our Bayesian hierarchical approach would be more challenging for reasons including computational feasibility, inability to assign each region-region link to a single brain network, and the presence of strong collinearity in the data - a scenario for which PLS, however, is a natural choice. For the diffusion brain scans, in turn, microstructure of interregional fiber tracts (48 measurements) of each participant were associated with trait loneliness using Pearson’s correlation, while uncertainty in effect sizes was evaluated based on bootstrapping resampling distributions. The anatomical tract properties were analyzed with the simplest approach that answered our research question at hand. This is because no obvious hierarchical structure suggested itself (in contrast to structural MRI) and exploiting collinearity was less urgent (in contrast to functional MRI).

### Data resources

The UK Biobank is a prospective epidemiology resource that offers extensive behavioral and demographic assessments, medical and cognitive measures, as well as biological samples in a cohort of ~500,000 participants recruited from across Great Britain (https://www.ukbiobank.ac.uk/). This openly accessible population dataset aims to provide multi-modal brain imaging for ~100,000 individuals planned for completion in 2022. The present study was based on the recent data release from February/March 2020. To improve comparability and reproducibility, our study built on the uniform data preprocessing pipelines designed and carried out by FMRIB, Oxford University, UK^58^. Our study involved data from 38,701 participants with brain-imaging measures of gray matter morphology (T1-weighted MRI [sMRI]), white matter microstructure (diffusion MRI [dMRI]), and neural activity fluctuations (resting-state functional MRI [fMRI]) from 47.5% men and 52.5% women, aged 40–69 years when recruited (mean age 54.9, standard deviation [SD] 7.5 years; see Supplementary Table 2 for sociodemographic characteristics of the sample). The present analyses were conducted under UK Biobank application number 25163. All participants provided written, informed consent and the study was approved by the Research Ethics Committee (REC number 11/NW/0382). Further information on the consent procedure can be found elsewhere (http://biobank.ctsu.ox.ac.uk/crystal/field.cgi?id=200).

Our study focused on trait loneliness, which was based on the following question: “Do you often feel lonely?” (yes = 1, no = 0). The need for brief loneliness assessments has long been recognized, particularly for inclusion in large population-based studies^59^. Self-reported binary loneliness assessment shows strong convergent validity with more extensive self-report measures^60^. Indeed, in the original validation study of the UCLA loneliness scale, which is considered a gold standard assessment, Russell et al.^61^ reported strong and reliable correlations with a single-item loneliness measure (rho = 0.79). As additional support of our target measure, in a systematic review of loneliness and health in older adulthood, Ong et al.^59^ identified numerous studies that have successfully used a single item to assess the experience of loneliness (e.g., refs. ^62,63^). Consistent with this standard, several behavioral and health-related studies have now reported results using the UK Biobank measure of trait loneliness^64–67^.

### Multimodal brain imaging and preprocessing procedures

Magnetic resonance imaging (MRI) scanners (3T Siemens Skyra) were matched at several dedicated imaging sites with the same acquisition protocols and standard Siemens 32-channel radiofrequency receiver head coils. To protect the anonymity of the study participants, brain-imaging data were defaced and any sensitive meta-information was removed. Automated processing and quality control pipelines were deployed^58,68^. To improve homogeneity of the imaging data, noise was removed by means of 190 sensitivity features. This approach allowed for the reliable identification and exclusion of problematic brain scans, such as due to excessive head motion.

#### Structural MRI

The sMRI data were acquired as high-resolution T1-weighted images of brain anatomy using a 3D MPRAGE sequence at 1 mm isotropic resolution. Preprocessing included gradient distortion correction (GDC), field of view reduction using the Brain Extraction Tool^69^ and FLIRT^70,71^, as well as non-linear registration to MNI152 standard space at 1 mm resolution using FNIRT^72^ (tools part of FMRIB Software Library v6.0). To avoid unnecessary interpolation, all image transformations were estimated, combined and applied by a single interpolation step. Tissue-type segmentation into cerebrospinal fluid (CSF), gray matter (GM), and white matter (WM) was applied using FAST (FMRIB’s Automated Segmentation Tool)^73^ to generate full bias-field-corrected images. SIENAX^74^, in turn, was used to derive volumetric measures normalized for head sizes.

#### Resting-state functional MRI

The fMRI data of intrinsic brain activity were acquired without engagement in a predefined experimental task context at 2.4 mm spatial resolution, time to repeat = 0.735 s, and with multiband acceleration of 8. A single-band reference image with higher between-tissue contrast and without T1-saturation effects was acquired within the same geometry as the time series of neural activity maps. The reference scan was used for the alignment to other brain-imaging modalities and correction for head motion. Preprocessing was performed using MELODIC^75^ (tools part of FMRIB Software Library v6.0), including EPI and GDC unwarping, motion correction, grand-mean intensity normalization, and high-pass temporal filtering (Gaussian-weighted least-squares straight line fitting, sigma = 50 s). The ensuing images were submitted to motion correction using MCFLIRT^70^. Structured artefacts were removed by combining ICA and FMRIB’s ICA-based X-noiseifier^76^. To help reduce unnecessary interpolation effects, all intermediate warp operations were merged into a composite transformation allowing for simultaneous application to fMRI maps.

#### Diffusion-weighted MRI

All dMRI data were encoded in AP direction and acquired with two *b*-values (*b* = 1000 and *b* = 2000 s/mm^3^) at 2 mm spatial resolution. Preprocessing included eddy current-induced distortion correction, correction for head motion, and removal of outlier brain slices. GDC was applied after eddy correction to avoid moving data out of the image plane^77^. In all, 50 distinct diffusion-encoding directions were acquired. Diffusion tensor imaging (DTI) then yielded several quantities of fiber-tract anatomy, including the widely used fractional anisotropy (FA). Modeling DTI parameters was based on tract-based spatial statistics (TBSS, tools part of FMRIB Software Library v6.0)^78^. All FA images were aligned to create a group mean FA skeleton using high-dimensional non-linear registration^79^.

### Analysis of associations between loneliness and gray matter structure

Neurobiologically interpretable measures of gray matter volume were extracted in all participants by summarizing whole-brain sMRI maps in MNI reference space. This feature-generation step was guided by the topographical brain region definitions of the commonly used Schaefer-Yeo atlas comprising 100 parcels^37^. The derived quantities of local gray matter morphology comprised 100 volume measures for each participant. The subject-level brain region volumes provided the input variables for our Bayesian hierarchical modeling approach (cf. below). As a data-cleaning step, inter-individual variation in brain region volumes that could be explained by nuisance variables of no interest were regressed out: body mass index, head size, head motion during task-related brain scans, head motion during resting-state fMRI scanning, head position and receiver coil in the scanner (x, y, and z), position of scanner table, as well as the data acquisition site.

To examine normative population variation of our atlas regions in the context of trait loneliness, Bayesian hierarchical modeling was a natural choice of method, building on our previous work on joint region-network modeling^80–82^. In contrast, classical linear regression combined with statistical significance testing would simply have provided *p*-values against the null hypothesis of no difference between lonely and non-lonely participants in each brain region. Instead of limiting our results and conclusions to strict categorical statements, each region being either relevant or not, our analytical strategy aimed at full probability distributions that expose how brain region volumes converge or diverge in their relation to loneliness to the extent supported by the UK Biobank population. In this way, our approach provided coherent, continuous estimates of uncertainty for each model parameter at play for its relevance in loneliness. Our study thus addressed the question ‘How certain are we that a regional brain volume is divergent between lonely and non-lonely individuals?’. This analysis did not ask ‘Is there a strict categorical difference in region volume between lonely and non-lonely individuals?’.

The elected Bayesian hierarchical framework also enabled simultaneous modeling of multiple organizational principles: segregation into separate brain regions and integration into groups of brain regions by spatially distributed brain networks. Two regions of the same brain network are more likely to exhibit correlated volume associations than two regions belonging to two distinct brain networks. Each of the 100 region definitions was pre-assigned to one of the seven large-scale network definitions in the Schaefer-Yeo atlas^37^, providing a native multilevel structure. Setting up a hierarchical generative process enabled our analytical approach to borrow statistical strength between model parameters at the higher network level and model parameters of the lower level of constituent brain regions. By virtue of partial pooling, the brain region parameters were modeled themselves by the hyper-parameters of the hierarchical regression as a function of the network hierarchy to explain trait loneliness. Assigning informative priors, centered around zero, provided an additional form of regularization by shrinking coefficients to zero in the absence of evidence to the contrary. We could thus provide fully probabilistic answers to questions about the morphological relevance of individual local brain regions and distributed cortical networks by a joint varying-effects estimation that profited from several neurobiologically meaningful sources of population variation.

The first model specification was tailored to careful inference of the posterior distributions of parameters at the brain region level to explain possible divergence between lonely and non-lonely individuals (model equation (1)):

$$y\sim {\mathrm{Bernoulli}}\left( p \right)$$

$$\begin{array}{l}{\mathrm{logit}}\left( p \right) = {\mathbf{x}}_1 \ast \beta _{{\mathrm{region}}_1} + \ldots + {\mathbf{x}}_{100} \ast \beta _{{\mathrm{region}}_{100}} + {}\\ \alpha _{{\mathrm{men}}\left[ {{\mathrm{sex}}} \right]} + \alpha _{{\mathrm{women}}\left[ {{\mathrm{sex}}} \right]} + \alpha _{{\mathrm{men}}\_{\mathrm{age}}\left[ {{\mathrm{sex}}} \right]} \ast {\mathrm{age}}_{{\mathrm{men}}} + \alpha _{{\mathrm{women}}\_{\mathrm{age}}\left[ {{\mathrm{sex}}} \right]} \ast {\mathrm{age}}_{{\mathrm{women}}}\end{array}$$

$$\beta _{{\mathrm{region}}_{{\mathrm{network}}\_{\mathrm{Visual}}}}\sim {\mathrm{MVNormal}}\left( {\left[ {\begin{array}{*{20}{c}} 0 \\ \vdots \\ 0 \end{array}} \right],\,{{\Sigma }}_{{\mathrm{Visual}}}} \right);{{\Sigma }}_{{\mathrm{Visual}}} = \left[ {\begin{array}{*{20}{c}} {\sigma _{o1}^2} & \cdots & {\,} \\ \vdots & \ddots & \vdots \\ {\,} & \cdots & {\sigma _{ot}^2} \end{array}} \right]$$

$$\beta _{{\mathrm{region}}_{{\mathrm{netwok}}\_{\mathrm{SomMot}}}}\sim {\mathrm{MVNormal}}\left( {\left[ {\begin{array}{*{20}{c}} 0 \\ \vdots \\ 0 \end{array}} \right],\,{{\Sigma }}_{{\mathrm{SomMot}}}} \right);{{\Sigma }}_{{\mathrm{SomMot}}} = \left[ {\begin{array}{*{20}{c}} {\sigma _{p1}^2} & \cdots & {\,} \\ \vdots & \ddots & \vdots \\ {\,} & \cdots & {\sigma _{pu}^2} \end{array}} \right]$$

$$\beta _{{\mathrm{region}}_{{\mathrm{network}}\_{\mathrm{Limbic}}}}\sim {\mathrm{MVNormal}}\left( {\left[ {\begin{array}{*{20}{c}} 0 \\ \vdots \\ 0 \end{array}} \right],{{\Sigma }}_{{\mathrm{Limbic}}}} \right);{{\Sigma }}_{{\mathrm{Limbic}}} = \left[ {\begin{array}{*{20}{c}} {\sigma _{q_1}^2} & \cdots & {\,} \\ \vdots & \ddots & \vdots \\ {\,} & \cdots & {\sigma _{qv}^2} \end{array}} \right]$$

$$\beta _{{\mathrm{region}}_{{\mathrm{network}}\_{\mathrm{Salience}}}}\sim {\mathrm{MVNormal}}\left( {\left[ {\begin{array}{*{20}{c}} 0 \\ \vdots \\ 0 \end{array}} \right],\Sigma _{{\mathrm{Salience}}}} \right);\Sigma _{{\mathrm{Salience}}} = \left[ {\begin{array}{*{20}{c}} {\sigma _{r1}^2} & \cdots & {\,} \\ \vdots & \ddots & \vdots \\ {\,} & \cdots & {\sigma _{r_w}^2} \end{array}} \right]$$

$$\beta _{{\mathrm{region}}_{{\mathrm{network}}\_{\mathrm{Control}}}}\sim {\mathrm{MVNormal}}\left( {\left[ {\begin{array}{*{20}{c}} 0 \\ \vdots \\ 0 \end{array}} \right],{{\Sigma }}_{{\mathrm{Control}}}} \right);{{\Sigma }}_{{\mathrm{Control}}} = \left[ {\begin{array}{*{20}{c}} {\sigma _{s1}^2} & \cdots & {\,} \\ \vdots & \ddots & \vdots \\ {\,} & \cdots & {\sigma _{s_x}^2} \end{array}} \right]$$

$$\beta _{{\mathrm{region}}_{{\mathrm{network}}\_{\mathrm{DorsalAttn}}}}\sim {\mathrm{MVNormal}}\left( {\left[ {\begin{array}{*{20}{c}} 0 \\ \vdots \\ 0 \end{array}} \right],{{\Sigma }}_{{\mathrm{DorsalAttn}}}} \right);{{\Sigma }}_{{\mathrm{DorsalAttn}}} = \left[ {\begin{array}{*{20}{c}} {\sigma _{t1}^2} & \cdots & {\,} \\ \vdots & \ddots & \vdots \\ {\,} & \cdots & {\sigma _{t_y}^2} \end{array}} \right]$$

$$\beta _{{\mathrm{region}}_{{\mathrm{network}}\_{\mathrm{Default}}}}\sim {\mathrm{MVNormal}}\left( {\left[ {\begin{array}{*{20}{c}} 0 \\ \vdots \\ 0 \end{array}} \right],{{\Sigma }}_{{\mathrm{Default}}}} \right);{{\Sigma }}_{{\mathrm{Default}}} = \left[ {\begin{array}{*{20}{c}} {\sigma _{u_1}^2} & \cdots & {\,} \\ \vdots & \ddots & \vdots \\ {\,} & \cdots & {\sigma _{u_z}^2} \end{array}} \right]$$

$$\alpha _{{\mathrm{men}}}\sim {\cal{N}}\left( {0,\,1} \right)$$

$$\alpha _{{\mathrm{women}}}\sim {\cal{N}}\left( {0,\,1} \right)$$

$$\alpha _{{\mathrm{men}}\_{\mathrm{age}}}\sim {\cal{N}}\left( {0,\,1} \right)$$

$$\alpha _{{\mathrm{women}}\_{\mathrm{age}}}\sim {\cal{N}}\left( {0,1} \right)$$

where *β* denote the slopes for the brain volumes *x* for all 100 regions of the Schaefer-Yeo atlas (*z*-scored across participants), *y* denotes trait loneliness of each participants, and the hyper-parameters capture volume variation at the network level through seven multivariate Gaussian distributions (MVNormal*)* that jointly inform parameters at the region level. The parameters of the network covariance matrices were directly inferred from the data. The estimation of these covariance relationships was guided by the LKJ correlation prior^83^. Potential confounding influences were acknowledged by the nuisance variables *α*, accounting for variation that could be explained by sex and (*z*-scored) age.

The second model specification put emphasis on careful inference of unique posterior distributions of parameters at the brain network level to discriminate lonely versus non-lonely individuals (model equation (2)):

$$y\sim {\mathrm{Bernoulli}}\left( p \right)$$

$$\begin{array}{l}{\mathrm{logit}}\left( p \right) = {\mathbf{x}}_1 \ast \beta _{{\mathrm{region}}1\left[ g \right]} + \ldots + {\mathbf{x}}_{100} \ast \beta _{{\mathrm{region}}100\left[ g \right]} + {}\\ \alpha _{{\mathrm{men}}\left[ {{\mathrm{sex}}} \right]} + \alpha _{{\mathrm{women}}\left[ {{\mathrm{sex}}} \right]} + \alpha _{{\mathrm{men}}\_{\mathrm{age}}\left[ {{\mathrm{sex}}} \right]} \ast age_{{\mathrm{men}}} + \alpha _{{\mathrm{women}}\_{\mathrm{age}}\left[ {{\mathrm{sex}}} \right]} \ast age_{{\mathrm{women}}}\end{array}$$

$$\beta _{{\mathrm{region}}_{{\mathrm{network}}\_{\mathrm{Visual}}}}\sim {\mathrm{MVNormal}}\left( {\left[ {\begin{array}{*{20}{c}} 0 \\ \vdots \\ 0 \end{array}} \right],{{\Sigma }}_{{\mathrm{Visual}}}} \right);{{\Sigma }}_{{\mathrm{Visual}}} = \left[ {\begin{array}{*{20}{c}} {\sigma _o^2} & \cdots & {\,} \\ \vdots & \ddots & \vdots \\ {\,} & \cdots & {\sigma _o^2} \end{array}} \right]$$

$$\beta _{{\mathrm{region}}_{{\mathrm{netwok}}\_{\mathrm{SomMot}}}}\sim {\mathrm{MVNormal}}\left( {\left[ {\begin{array}{*{20}{c}} 0 \\ \vdots \\ 0 \end{array}} \right],{{\Sigma }}_{{\mathrm{SomMot}}}} \right);{{\Sigma }}_{{\mathrm{SomMot}}} = \left[ {\begin{array}{*{20}{c}} {\sigma _p^2} & \cdots & {\,} \\ \vdots & \ddots & \vdots \\ {\,} & \cdots & {\sigma _p^2} \end{array}} \right]$$

$$\beta _{{\mathrm{region}}_{{\mathrm{network}}\_{\mathrm{Limbic}}}}\sim {\mathrm{MVNormal}}\left( {\left[ {\begin{array}{*{20}{c}} 0 \\ \vdots \\ 0 \end{array}} \right],{{\Sigma }}_{{\mathrm{Limbic}}}} \right);{{\Sigma }}_{{\mathrm{Limbic}}} = \left[ {\begin{array}{*{20}{c}} {\sigma _q^2} & \cdots & {\,} \\ \vdots & \ddots & \vdots \\ {\,} & \cdots & {\sigma _q^2} \end{array}} \right]$$

$$\beta _{{\mathrm{region}}_{{\mathrm{network}}\_{\mathrm{Salience}}}}\sim {\mathrm{MVNormal}}\left( {\left[ {\begin{array}{*{20}{c}} 0 \\ \vdots \\ 0 \end{array}} \right],{{\Sigma }}_{{\mathrm{Salience}}}} \right);{{\Sigma }}_{{\mathrm{Salience}}} = \left[ {\begin{array}{*{20}{c}} {\sigma _r^2} & \cdots & {\,} \\ \vdots & \ddots & \vdots \\ {\,} & \cdots & {\sigma _r^2} \end{array}} \right]$$

$$\beta _{{\mathrm{region}}_{{\mathrm{network}}\_{\mathrm{Control}}}}\sim {\mathrm{MVNormal}}\left( {\left[ {\begin{array}{*{20}{c}} 0 \\ \vdots \\ 0 \end{array}} \right],{{\Sigma }}_{{\mathrm{Control}}}} \right);{{\Sigma }}_{{\mathrm{Control}}} = \left[ {\begin{array}{*{20}{c}} {\sigma _s^2} & \cdots & {\,} \\ \vdots & \ddots & \vdots \\ {\,} & \cdots & {\sigma _s^2} \end{array}} \right]$$

$$\beta _{{\mathrm{region}}_{{\mathrm{network}}\_{\mathrm{DorsalAttn}}}}\sim {\mathrm{MVNormal}}\left( {\left[ {\begin{array}{*{20}{c}} 0 \\ \vdots \\ 0 \end{array}} \right],{{\Sigma }}_{{\mathrm{DorsalAttn}}}} \right);{{\Sigma }}_{{\mathrm{DorsalAttn}}} = \left[ {\begin{array}{*{20}{c}} {\sigma _t^2} & \cdots & {\,} \\ \vdots & \ddots & \vdots \\ {\,} & \cdots & {\sigma _t^2} \end{array}} \right]$$

$$\beta _{{\mathrm{region}}_{{\mathrm{network}}\_{\mathrm{Default}}}}\sim {\mathrm{MVNormal}}\left( {\left[ {\begin{array}{*{20}{c}} 0 \\ \vdots \\ 0 \end{array}} \right],{{\Sigma }}_{{\mathrm{Default}}}} \right);{{\Sigma }}_{{\mathrm{Default}}} = \left[ {\begin{array}{*{20}{c}} {\sigma _u^2} & \cdots & {\,} \\ \vdots & \ddots & \vdots \\ {\,} & \cdots & {\sigma _u^2} \end{array}} \right]$$

$$\alpha _{{\mathrm{men}}}\sim {\cal{N}}\left( {0,1} \right)$$

$$\alpha _{{\mathrm{women}}}\sim {\cal{N}}\left( {0,1} \right)$$

$$\alpha _{{\mathrm{men}}\_{\mathrm{age}}}\sim {\cal{N}}\left( {0,1} \right)$$

$$\alpha _{{\mathrm{women}}\_{\mathrm{age}}}\sim {\cal{N}}\left( {0,1} \right)$$

where Σ parameters estimated the overall variance across the regions that belong to a given canonical network, independent of whether the volume associations of the respective constituent brain regions had positive or negative direction. As such, the network variance parameters Σ directly quantified the magnitude of intra-network coefficients, and thus the overall relevance of a given network in explaining loneliness based on the dependent region morphology measures. All regions belonging to the same brain network share the same variance parameter in the diagonal of the covariance matrix, while off-diagonal covariance relationships are zero. The index *g* either collapsed to a single group or extended the model to jointly estimate parameters specific for men and women.

Probabilistic posterior distributions for all model parameters were estimated for the hierarchical models. Our Bayesian approach could thus simultaneously appreciate gray matter variation in segregated brain regions as well as in integrative brain networks in a population cohort. The approximation of the posterior distributions was carried out by the NUTS sampler^84^, a type of Markov chain Monte Carlo (MCMC), using the PyMC3 software (Python package, version 3.8)^85^. After tuning the sampler for 4000 steps, we drew 1000 samples from the joint posterior distribution over the full set of parameters in the model for analysis. Proper convergence was assessed by ensuring Rhat measures^84^ stayed below 1.02.

### Analysis of associations between loneliness and functional connectivity patterns

Quantitative measures of functional connectivity were computed for the same 100 brain regions as defined by the Schaefer-Yeo atlas^37^. Functional connectivity profiles for each participant were derived by computing Pearson’s correlation between their neural activity fluctuations. To this end, in each participant, the time series of whole-brain fMRI signals, obtained in a task-free manner, were summarized by averaging for each brain region in the atlas. The approach yielded the functional coupling signature of the whole cortex as a 100 × 100 region coupling matrix for each participant. The ensuing region-region coupling estimates underwent standardization across participants by centering to zero mean and unit scaling to a variance of one (cf. next step). Inter-individual variation in the functional coupling strengths between brain regions that could be explained by nuisance variables of no interest were regressed out in a data-cleaning step: body mass index, head size, head motion during task-related brain scans, head motion during resting-state fMRI scanning, head position as well as receiver coil in the scanner (x, y, and z), position of scanner table, and data acquisition site.

We then sought the dominant coupling regime - “mode” of population covariation - that provides insight into how functional variability in 100 brain regions can explain trait loneliness. Partial least squares was a natural choice of method (as implemented in Python package sklearn, version 0.21.3) to decompose the obtained 100 × 100 matrix of functional coupling patterns with respect to loneliness. The variable set *X* was constructed from the lower triangle of the participants’ functional coupling matrices. The target vector *y* encoded lonely participants as +1 and non-lonely participants as −1. In general, PLS involves finding the matrix factorization into *k* low-rank brain representations that maximize the correspondence with our social trait of interest. In our study, PLS was thus used to identify the most explanatory projection that yielded maximal covariance between sets of region couplings in the context of participant reports of perceived social isolation.

In other words, the extracted chief functional coupling mode identified linear combinations of cortical brain connections that featured the best correspondence to loneliness. Concretely, positive (negative) modulation weights revealed increased (decreased) correlation strengths, relative to average functional coupling. This is because the computed functional connectivity estimates were initially normalized to zero mean and unit variance across participants. For instance, a functional connectivity input into PLS of 0 denoted the average functional coupling strength in our UK Biobank sample, rather than an absence of functional connectivity between the region pair. The derived PLS weights thus indicated deviations from average functional coupling patterns in our cohort. Moreover, the variable sets were entered into PLS after a confound-removal procedure (cf. above).

The resulting dominant PLS mode of loneliness-related functional coupling deviations was assessed for statistical robustness in a non-parametric permutation procedure, which is in line with previous research^68^. Relying on minimal modeling assumptions, a valid empirical null distribution was derived for the achieved Pearson’s correlation between low-rank projections of each mode resulting from PLS analysis. In 1000 permutation iterations, the functional connectivity matrix was held constant, while the loneliness labels were submitted to random shuffling. The constructed surrogate data preserved the statistical structure idiosyncratic to the fMRI signals, yet permitted to selectively destroy the signal property related to loneliness^86^. The generated distribution of the test statistic reflected the null hypothesis of random association between the brain’s functional coupling and loneliness status across participants. The Pearson’s correlations rho between the perturbed low-rank projection was recorded in each iteration. *P*-values were derived based on the 1000 Pearson’s rho estimates from the null PLS model. Correction for multiple comparisons was not necessary since our study considered exclusively the leading functional coupling mode as estimated by PLS, which turned out to be statistically significant at *p* < 0.05.

### Analysis of associations between loneliness and white matter structure

In each participant, mean FA data (cf. above) from the local center of the neighboring tract were projected onto the common-space FA skeleton. For each voxel of the mean skeleton, the FA image of a given individual was searched perpendicularly to the tract direction to locate the subject-specific optimal FA value representing the fiber tract in that individual. Each voxel FA value was thus assigned to the representative location in the mean FA skeleton. The standard-space warping strategy was applied to ensure precise alignment of fiber-tract topography between individuals. For the mean FA dMRI parameter, white matter summary measures were extracted by computing the average within regions defined as the intersection of the skeleton with masks for 48 tracts from the widely used Johns Hopkins University (JHU) atlas^87^, and translated into corresponding image-derived phenotypes (IDPs).

This approach resulted in a set of summary estimates of fiber bundle microstructure for each of the 48 tracts from the JHU atlas. Inter-individual variation in brain fiber tract features that could be explained by nuisance variables of no interest were regressed out as a data-cleaning step: body mass index, head size, head motion during task-related brain scans, head motion during resting-state fMRI scanning, head position as well as receiver coil in the scanner (x, y, and z), position of scanner table, and data acquisition site.

The measures of tract microstructure were then submitted to assessment for linear (Pearson’s rho) and non-linear (Spearman’s rho) associations. To get a sense of uncertainty in the tract-loneliness associations, we carried out a bootstrap analysis with 100 resampling iterations. This procedure mimicked the participant recruitment process by repeating the association analysis based on 100 perturbed participant samples, selected with replacement. The ensuing 5–95% bootstrap confidence intervals quantified the expected variation in the effect size (correlation coefficient) if other participants would have been drawn from the same underlying population. Correction for multiple comparisons was carried out based on Bonferroni’s method.

### Reporting summary

Further information on research design is available in the Nature Research Reporting Summary linked to this article.

## Supplementary information

Supplementary Information

Reporting Summary

## Data Availability

All used data are available to other investigators online (ukbiobank.ac.uk). The John-Hopkins atlas is accessible online (https://fsl.fmrib.ox.ac.uk/fsl/fslwiki/Atlases). The Schaefer-Yeo atlas is accessible online (https://github.com/ThomasYeoLab/CBIG/tree/master/stable_projects/brain_parcellation/Schaefer2018_LocalGlobal). Source data are provided with this paper.

## Source data

Source Data
