# Publisher Correction: The default network of the human brain is associated with perceived social isolation

**DOI:** 10.1038/s41467-021-23623-w

**Published:** 2021-05-21

**Authors:** R. Nathan Spreng, Emile Dimas, Laetitia Mwilambwe-Tshilobo, Alain Dagher, Philipp Koellinger, Gideon Nave, Anthony Ong, Julius M. Kernbach, Thomas V. Wiecki, Tian Ge, Yue Li, Avram J. Holmes, B. T. Thomas Yeo, Gary R. Turner, Robin I. M. Dunbar, Danilo Bzdok

**Affiliations:** 1 Laboratory of Brain and Cognition, Montreal Neurological Institute, Department of Neurology and Neurosurgery, Faculty of Medicine, McGill University, Montreal, QC Canada; 2 Departments of Psychiatry and Psychology, McGill University, Montreal, QC Canada; 3 Douglas Mental Health University Institute, Verdun, QC HRH 1R3 Canada; 4 McConnell Brain Imaging Centre, Montreal Neurological Institute (MNI), McGill University, Montreal, QC Canada; 5 Department of Biomedical Engineering, Faculty of Medicine, McGill University, Montreal, QC Canada; 6 School of Business and Economics, Vrije Universiteit Amsterdam, Amsterdam, The Netherlands; 7 Marketing Department, the Wharton School, University of Pennsylvania, Pennsylvania, PA USA; 8 Department of Human Development, Cornell University, Ithaca, NY USA; 9 Division of Geriatrics and Palliative Medicine, Weill Cornell Medical College, New York, NY USA; 10 Department of Neurosurgery, Neurosurgical Artificial Intelligence Laboratory Aachen (NAILA), RWTH Aachen University Hospital, Aachen, Germany; 11 Quantopian Inc., Boston, MA USA; 12 Psychiatric and Neurodevelopmental Genetics Unit, Center for Genomic Medicine, Massachusetts General Hospital, Boston, MA 02114 USA; 13 Department of Psychiatry, Massachusetts General Hospital, Harvard Medical School, Boston, MA 02114 USA; 14 Athinoula A. Martinos Center for Biomedical Imaging, Massachusetts General Hospital, Charlestown, MA 02129 USA; 15 Stanley Center for Psychiatric Research, Broad Institute of MIT and Harvard, Cambridge, MA 02138 USA; 16 School of Computer Science, McGill University, Montreal, QC Canada; 17 Departments of Psychology and Psychiatry, Yale University, New Haven, CA 06520 USA; 18 Department of Electrical & Computer Engineering, Centre for Sleep & Cognition, Clinical Imaging Research Centre, N.1 Institute for Health, National University of Singapore, Singapore, Singapore; 19 Department of Psychology, York University, Toronto, ON Canada; 20 Department of Experimental Psychology, University of Oxford, Oxford, UK; 21 Mila - Quebec Artificial Intelligence Institute, Montreal, QC Canada

**Keywords:** Neuroscience, Health care

Correction to: *Nature Communications* 10.1038/s41467-020-20039-w, published online 15 December 2020.

The original version of this Article contained an error in the Peer review information. The Peer review information section originally incorrectly read:

“Nature Communications thanks Meghan Meyer, Theodore Satterthwaite and the other, anonymous, reviewer(s) for their contribution to the peer review of this work. Peer reviewer reports are available.”

The correct version states:

“*Nature Communications* thanks Meghan Meyer, Theodore Satterthwaite and the other, anonymous, reviewer(s) for their contribution to the peer review of this work.”

This has been corrected in both the PDF and HTML versions of the Article.

