# Supplementary material for: The default network of the human brain is associated with perceived social isolation
Source: Nat Commun. 2020 Dec 15;11:6393. doi: 10.1038/s41467-020-20039-w (PMC7738683; doi:10.1038/s41467-020-20039-w)
Supplement: Supplementary file 1 — Supplementary Information [file 41467_2020_20039_MOESM1_ESM.pdf]

Supplementary information

Supplementary Table 1: Sociodemographic characteristics

	**Lonely**	**Non-Lonely**
Overall participants	13.10%	86.90%
Age (*years*)	53.62 (+/- 7.35 SD)	55.03 (+/- 7.47 SD)
Sex (*men : women*)	38.63% : 61.37%	48.61% : 51.39%
Fluid IQ (0=*low, 13=high*)	6.49 (+/- 2.04 SD)	6.74 (+/- 2.03 SD)
Amount of social support / ability to confide to someone close (0=*almost never, 5=almost daily*)	2.77 (+/- 1.95 SD)	3.86 (+/- 1.73 SD)
Number of people living in household	2.34 (+/- 1.31 SD)	2.60 (+/- 1.23 SD)
Age completed education (*years*)	16.94 (+/- 2.96 SD)	17.09 (+/- 2.75 SD)
Adiposity (*waist circumference in cm*)	88.15 (+/- 13.23 SD)	87.81 (+/- 12.38 SD)
Alcohol intake (*1=almost daily, 6=never*)	2.91 (+/- 1.45 SD)	2.65 (+/- 1.38 SD)
Neuroticism (*12=high, 0=low*)	7.58 (+/- 2.88 SD)	3.21 (+/- 2.78 SD)
Number of depression episodes	9.91 (+/- 40.81 SD)	4.91 (+/- 27.97 SD)
Recent feelings or nervousness or anxiety (*1=not at all, 4=nearly every day*)	1.61 (+/- 0.80 SD)	1.28 (+/- 0.56 SD)
Dissatisfaction with friendships (*1=extremely happy, 6=extremely unhappy*)	2.70 (+/- 1.02 SD)	2.21 (+/- 0.74 SD)
Dissatisfaction with family relationships (*1=extremely happy, 6=extremely unhappy*)	2.76 (+/- 1.00 SD)	2.14 (+/- 0.82 SD)

Supplementary Table 2a: Genetic correlations between loneliness and other phenotypes (pvalue_bonf, corrected for multiple comparisons using Bonferroni's method).

phenotype 1	phenotype 2	Rg	se	z	pvalue	pvalue_bonf
loneliness	Age at first live birth	-0.4086	0.0339	-12.0411	2.16E-33	1.67E-30
loneliness	Age at last live birth	-0.2877	0.0447	-6.4388	1.20E-10	9.29E-08
loneliness	Age completed full time education	-0.3611	0.0318	-11.3561	6.92E-30	5.36E-27
loneliness	Age of first birth	-0.31	0.036	-8.6068	7.52E-18	5.82E-15
loneliness	Age started oral contraceptive pill	-0.289	0.0486	-5.9427	2.80E-09	2.17E-06
loneliness	Alcohol drinker status: Previous	0.3947	0.0545	7.2434	4.37E-13	3.38E-10
loneliness	Alcohol intake frequency.	0.319	0.0297	10.7506	5.89E-27	4.56E-24
loneliness	Alcohol intake versus 10 years previously	0.3657	0.0377	9.7075	2.80E-22	2.17E-19
loneliness	Alcohol usually taken with meals	-0.3104	0.0304	-10.2162	1.68E-24	1.30E-21
loneliness	Arm fat mass (left)	0.2317	0.023	10.056	8.64E-24	6.69E-21
loneliness	Arm fat mass (right)	0.2336	0.0235	9.9554	2.39E-23	1.85E-20
loneliness	Arm fat percentage (left)	0.2317	0.024	9.6542	4.72E-22	3.65E-19
loneliness	Arm fat percentage (right)	0.2343	0.0244	9.6113	7.16E-22	5.54E-19
loneliness	Arm fat-free mass (left)	0.127	0.0234	5.4279	5.70E-08	4.41E-05
loneliness	Arm fat-free mass (right)	0.1142	0.0234	4.8741	1.09E-06	0.00084366
loneliness	Arm predicted mass (left)	0.1236	0.0236	5.2446	1.57E-07	0.000121518
loneliness	Arm predicted mass (right)	0.1152	0.0235	4.8983	9.67E-07	0.000748458
loneliness	Average weekly beer plus cider intake	0.1971	0.0413	4.7694	1.85E-06	0.0014319
loneliness	Average weekly champagne plus white wine intake	-0.322	0.0455	-7.0811	1.43E-12	1.11E-09
loneliness	Average weekly red wine intake	-0.2761	0.0377	-7.3187	2.50E-13	1.94E-10
loneliness	Back pain for 3+ months	0.316	0.0646	4.8955	9.81E-07	0.000759294
loneliness	Basal metabolic rate	0.1185	0.0229	5.1795	2.22E-07	0.000171828
loneliness	Bilateral oophorectomy (both ovaries removed)	0.2592	0.0574	4.5153	6.32E-06	0.00489168
loneliness	diagnosed by doctor: Asthma	0.1613	0.0309	5.2163	1.83E-07	0.000141642
loneliness	diagnosed by doctor: Blood clot in the lung	0.3416	0.081	4.216	2.49E-05	0.0192726
loneliness	diagnosed by doctor: Emphysema/chronic bronchitis	0.4357	0.0702	6.2065	5.42E-10	4.20E-07
loneliness	diagnosed by doctor: None of the above	-0.1282	0.0253	-5.0695	3.99E-07	0.000308826
loneliness	Body fat	0.2387	0.0415	5.7509	8.88E-09	6.87E-06
loneliness	Body fat percentage	0.2377	0.024	9.9212	3.37E-23	2.61E-20
loneliness	Body mass index	0.1797	0.0297	6.0507	1.44E-09	1.11E-06
loneliness	Body mass index (BMI)	0.2635	0.0238	11.0587	1.99E-28	1.54E-25
loneliness	Bring up phlegm/sputum/mucus on most days	0.4581	0.0923	4.9626	6.96E-07	0.000538704
loneliness	Chest pain or discomfort	0.5524	0.0351	15.7268	9.91E-56	7.67E-53
loneliness	Chest pain or discomfort walking normally	0.318	0.0556	5.716	1.09E-08	8.44E-06
loneliness	College completion	-0.426	0.0415	-10.2588	1.08E-24	8.36E-22
loneliness	Coronary artery disease	0.1509	0.0361	4.1837	2.87E-05	0.0222138
loneliness	Cough on most days	0.3544	0.0606	5.8515	4.87E-09	3.77E-06
loneliness	Creatinine (enzymatic) in urine	0.1735	0.0312	5.5696	2.55E-08	1.97E-05
loneliness	Current employment status: Unable to work because of sickness or disability	0.475	0.0416	11.4271	3.06E-30	2.37E-27
loneliness	Current tobacco smoking	0.3852	0.0349	11.0479	2.25E-28	1.74E-25
loneliness	Daytime dozing / sleeping (narcolepsy)	0.2031	0.033	6.1575	7.39E-10	5.72E-07
loneliness	Diabetes diagnosed by doctor	0.2422	0.0379	6.3868	1.69E-10	1.31E-07
loneliness	Diagnoses - main ICD10: D12 Benign neoplasm of colon_ rectum_ anus	0.3341	0.0649	5.1474	2.64E-07	0.000204336
loneliness	Diagnoses - main ICD10: G56 Mononeuropathies of upper limb	0.3025	0.0495	6.1129	9.79E-10	7.58E-07

Supplementary Table 2b: Genetic correlations between loneliness and other phenotypes (pvalue_bonf, corrected for multiple comparisons using Bonferroni's method).

phenotype 1	phenotype 2	Rg	se	z	pvalue	pvalue_bonf
loneliness	Diagnoses - main ICD10: I20 Angina pectoris	0.3833	0.0692	5.5362	3.09E-08	2.39E-05
loneliness	Diagnoses - main ICD10: I25 Chronic ischaemic heart disease	0.2003	0.0445	4.4981	6.86E-06	0.00530964
loneliness	Diagnoses - main ICD10: I84 Haemorrhoids	0.323	0.0636	5.079	3.79E-07	0.000293346
loneliness	Diagnoses - main ICD10: K21 Gastro-oesophageal reflux disease	0.3872	0.0861	4.4953	6.95E-06	0.0053793
loneliness	Diagnoses - main ICD10: K29 Gastritis and duodenitis	0.3165	0.0747	4.2392	2.24E-05	0.0173376
loneliness	Diagnoses - main ICD10: K44 Diaphragmatic hernia	0.4033	0.0951	4.2414	2.22E-05	0.0171828
loneliness	Diagnoses - main ICD10: K52 Other non-infective gastro-enteritis and colitis	0.5646	0.0999	5.6491	1.61E-08	1.25E-05
loneliness	Diagnoses - main ICD10: K62 Other diseases of anus and rectum	0.5289	0.1079	4.9034	9.42E-07	0.000729108
loneliness	Diagnoses - main ICD10: M17 Gonarthrosis [arthrosis of knee]	0.2646	0.054	4.8964	9.76E-07	0.000755424
loneliness	Diagnoses - main ICD10: M54 Dorsalgia	0.3417	0.0637	5.3603	8.31E-08	6.43E-05
loneliness	Diagnoses - main ICD10: R07 Pain in throat and chest	0.4766	0.0525	9.0776	1.11E-19	8.59E-17
loneliness	Diagnoses - main ICD10: R10 Abdominal and pelvic pain	0.4811	0.0653	7.3645	1.78E-13	1.38E-10
loneliness	Drive faster than motorway speed limit	-0.2081	0.0373	-5.5805	2.40E-08	1.86E-05
loneliness	Duration of moderate activity	0.2408	0.0439	5.4821	4.20E-08	3.25E-05
loneliness	Duration of walks	0.2626	0.0344	7.6376	2.21E-14	1.71E-11
loneliness	Ever depressed for a whole week	0.5124	0.0449	11.4029	4.04E-30	3.13E-27
loneliness	Ever had stillbirth_ spontaneous miscarriage or termination	0.3556	0.069	5.1571	2.51E-07	0.000194274
loneliness	Ever highly irritable/argumentative for 2 days	0.5888	0.0523	11.2523	2.26E-29	1.75E-26
loneliness	Ever manic/hyper for 2 days	0.6059	0.0952	6.3658	1.94E-10	1.50E-07
loneliness	Ever smoked	0.1806	0.0298	6.0592	1.37E-09	1.06E-06
loneliness	Ever unenthusiastic/disinterested for a whole week	0.6115	0.0453	13.4919	1.75E-41	1.35E-38
loneliness	Ever used hormone-replacement therapy (HRT)	0.3339	0.0412	8.1123	4.97E-16	3.85E-13
loneliness	Ever vs never smoked	0.2099	0.0463	4.5338	5.79E-06	0.00448146
loneliness	Excessive daytime sleepiness	0.2261	0.0465	4.8617	1.16E-06	0.00089784
loneliness	Exposure to tobacco smoke at home	0.4283	0.0658	6.5134	7.35E-11	5.69E-08
loneliness	Exposure to tobacco smoke outside home	0.3995	0.041	9.7424	1.99E-22	1.54E-19
loneliness	Falls in the last year	0.3317	0.0397	8.3514	6.74E-17	5.22E-14
loneliness	Family relationship satisfaction	0.5028	0.0397	12.6562	1.03E-36	7.97E-34
loneliness	Fathers age at death	-0.2883	0.0503	-5.7297	1.01E-08	7.82E-06
loneliness	Fathers age at death	-0.3163	0.0715	-4.4233	9.72E-06	0.00752328
loneliness	Fed-up feelings	0.7991	0.0208	38.4705	0	0
loneliness	Financial situation satisfaction	0.571	0.0448	12.7365	3.71E-37	2.87E-34
loneliness	Fluid intelligence score	-0.1996	0.032	-6.2402	4.37E-10	3.38E-07
loneliness	Forced expiratory volume in 1 second	-0.1058	0.023	-4.6039	4.15E-06	0.0032121
loneliness	Forced expiratory volume in 1 second	-0.0971	0.023	-4.2242	2.40E-05	0.018576
loneliness	Forced expiratory volume in 1-second (FEV1)	-0.1689	0.0274	-6.1617	7.20E-10	5.57E-07
loneliness	Forced expiratory volume in 1-second (FEV1)_ Best measure	-0.1662	0.0273	-6.0867	1.15E-09	8.90E-07
loneliness	Forced expiratory volume in 1-second (FEV1)_ predicted percentage	-0.1481	0.031	-4.7721	1.82E-06	0.00140868
loneliness	Forced vital capacity	-0.1104	0.0236	-4.6706	3.00E-06	0.002322
loneliness	Forced vital capacity	-0.1238	0.0241	-5.1375	2.78E-07	0.000215172
loneliness	Forced vital capacity (FVC)	-0.1665	0.0274	-6.0787	1.21E-09	9.37E-07
loneliness	Forced vital capacity (FVC)_ Best measure	-0.1717	0.0277	-6.2031	5.53E-10	4.28E-07
loneliness	Former vs Current smoker	-0.3512	0.0813	-4.3214	1.55E-05	0.011997
loneliness	Fractured bone site(s): Other bones	0.2797	0.0668	4.1854	2.85E-05	0.022059
loneliness	Frequency of depressed mood in last 2 weeks	0.8415	0.0237	35.5319	1.58E-276	1.22E-273
loneliness	Frequency of tenseness / restlessness in last 2 weeks	0.7477	0.0277	26.954	5.12E-160	3.96E-157
loneliness	Frequency of tiredness / lethargy in last 2 weeks	0.6971	0.0241	28.9654	1.80E-184	1.39E-181

Supplementary Table 2c: Genetic correlations between loneliness and other phenotypes (pvalue_bonf, corrected for multiple comparisons using Bonferroni's method).

phenotype 1	phenotype 2	Rg	se	z	pvalue	pvalue_bonf
loneliness	Frequency of travelling from home to job workplace	0.35	0.067	5.2242	1.75E-07	0.00013545
loneliness	Frequency of unenthusiasm / disinterest in last 2 weeks	0.8384	0.0263	31.9084	2.04E-223	1.58E-220
loneliness	Frequency of walking for pleasure in last 4 weeks	0.1703	0.0399	4.2645	2.00E-05	0.01548
loneliness	Friendships satisfaction	0.493	0.0395	12.4776	9.90E-36	7.66E-33
loneliness	Getting up in morning	-0.1737	0.0318	-5.4652	4.62E-08	3.58E-05
loneliness	Guilty feelings	0.5732	0.0276	20.7612	9.72E-96	7.52E-93
loneliness	Had major operations	0.3265	0.0624	5.2361	1.64E-07	0.000126936
loneliness	Hand grip strength (left)	-0.1641	0.0296	-5.5428	2.98E-08	2.31E-05
loneliness	Hand grip strength (right)	-0.1879	0.0309	-6.0889	1.14E-09	8.82E-07
loneliness	Happiness	0.5575	0.04	13.9216	4.68E-44	3.62E-41
loneliness	Health satisfaction	0.5732	0.0413	13.8641	1.05E-43	8.13E-41
loneliness	Hearing difficulty/problems with background noise	0.3186	0.0341	9.3514	8.65E-21	6.70E-18
loneliness	Hearing difficulty/problems: Yes	0.318	0.035	9.0995	9.07E-20	7.02E-17
loneliness	Hip circumference	0.1843	0.0234	7.8745	3.42E-15	2.65E-12
loneliness	Hip circumference	0.1375	0.028	4.9105	9.09E-07	0.000703566
loneliness	stress in last 2 years: Death of a close relative	0.3724	0.0812	4.5861	4.52E-06	0.00349848
loneliness	stress in last 2 years: Financial difficulties	0.574	0.0355	16.148	1.17E-58	9.06E-56
loneliness	stress in last 2 years: Marital separation/divorce	0.5041	0.1131	4.459	8.24E-06	0.00637776
loneliness	stress in last 2 years: None of the above	-0.5104	0.0413	-12.3635	4.12E-35	3.19E-32
loneliness	stress in last 2 years: Serious illness_ injury or assault to yourself	0.4916	0.0555	8.8639	7.73E-19	5.98E-16
loneliness	Illnesses of father: Chronic bronchitis/emphysema	0.365	0.0548	6.6603	2.73E-11	2.11E-08
loneliness	Illnesses of father: Lung cancer	0.2143	0.0505	4.2415	2.22E-05	0.0171828
loneliness	Illnesses of mother: Chronic bronchitis/emphysema	0.3848	0.0617	6.2386	4.42E-10	3.42E-07
loneliness	Illnesses of mother: Heart disease	0.2312	0.0568	4.0705	4.69E-05	0.0363006
loneliness	Illnesses of siblings: Heart disease	0.3399	0.0538	6.319	2.63E-10	2.04E-07
loneliness	Illnesses of siblings: None of the above (group 1)	-0.2464	0.0428	-5.758	8.51E-09	6.59E-06
loneliness	Illnesses of siblings: None of the above (group 2)	-0.4623	0.0918	-5.036	4.75E-07	0.00036765
loneliness	Illnesses of siblings: Severe depression	0.3861	0.0673	5.7329	9.87E-09	7.64E-06
loneliness	Impedance of arm (left)	-0.163	0.0244	-6.6793	2.40E-11	1.86E-08
loneliness	Impedance of arm (right)	-0.1568	0.0244	-6.4332	1.25E-10	9.68E-08
loneliness	Impedance of whole body	-0.1387	0.0241	-5.7541	8.71E-09	6.74E-06
loneliness	Insomnia	0.4188	0.0438	9.5722	1.05E-21	8.13E-19
loneliness	Insomnia	0.4641	0.0504	9.2105	3.24E-20	2.51E-17
loneliness	Intelligence	-0.2134	0.0393	-5.4367	5.43E-08	4.20E-05
loneliness	Irritability	0.5703	0.0257	22.1831	5.00E-109	3.87E-106
loneliness	Job involves heavy manual or physical work	0.4069	0.0335	12.149	5.81E-34	4.50E-31
loneliness	Job involves mainly walking or standing	0.2998	0.0335	8.9491	3.59E-19	2.78E-16
loneliness	Job involves shift work	0.3579	0.0496	7.2201	5.20E-13	4.02E-10
loneliness	Knee pain for 3+ months	0.3333	0.0807	4.1283	3.65E-05	0.028251
loneliness	Leg fat mass (left)	0.2547	0.0237	10.7599	5.32E-27	4.12E-24
loneliness	Leg fat mass (right)	0.2574	0.0238	10.8157	2.90E-27	2.24E-24
loneliness	Leg fat percentage (left)	0.2784	0.0246	11.3308	9.24E-30	7.15E-27
loneliness	Leg fat percentage (right)	0.2819	0.0247	11.4208	3.29E-30	2.55E-27
loneliness	Leg fat-free mass (left)	0.1112	0.0236	4.7101	2.48E-06	0.00191952
loneliness	Leg fat-free mass (right)	0.0962	0.0237	4.0574	4.96E-05	0.0383904
loneliness	Leg pain on walking	0.5134	0.0462	11.1077	1.15E-28	8.90E-26
loneliness	Leg predicted mass (left)	0.1114	0.0236	4.7314	2.23E-06	0.00172602
loneliness	Leg predicted mass (right)	0.097	0.0238	4.0798	4.51E-05	0.0349074

Supplementary Table 2d: Genetic correlations between loneliness and other phenotypes (pvalue_bonf, corrected for multiple comparisons using Bonferroni's method).

phenotype 1	phenotype 2	Rg	se	z	pvalue	pvalue_bonf
loneliness	Long-standing illness_ disability or infirmity	0.481	0.0313	15.3532	3.37E-53	2.61E-50
loneliness	Loud music exposure frequency	0.3202	0.0608	5.2615	1.43E-07	0.000110682
loneliness	Lung cancer	0.3166	0.0601	5.2637	1.41E-07	0.000109134
loneliness	Lung cancer (all)	0.3014	0.0683	4.4098	1.03E-05	0.0079722
loneliness	Major depressive disorder	0.6086	0.074	8.2213	2.01E-16	1.56E-13
loneliness	Maternal smoking around birth	0.3166	0.037	8.5501	1.23E-17	9.52E-15
loneliness	Maximum workload during fitness test	-0.3302	0.0782	-4.2224	2.42E-05	0.0187308
loneliness	Cholesterol lowering medication	0.1911	0.0459	4.1624	3.15E-05	0.024381
loneliness	Medication: Aspirin	0.2531	0.0464	5.4569	4.84E-08	3.75E-05
loneliness	Medication: Ibuprofen (e.g. Nurofen)	0.2835	0.0453	6.2577	3.91E-10	3.03E-07
loneliness	Medication: Laxatives (e.g. Dulcolax_ Senokot)	0.4467	0.0589	7.5885	3.24E-14	2.51E-11
loneliness	Medication: None of the above	-0.4246	0.0297	-14.3209	1.62E-46	1.25E-43
loneliness	Medication: Omeprazole (e.g. Zanprol)	0.3695	0.0511	7.2259	4.98E-13	3.85E-10
loneliness	Medication: Paracetamol	0.3701	0.0303	12.2218	2.38E-34	1.84E-31
loneliness	Miserableness	0.7562	0.0228	33.1282	1.17E-240	9.06E-238
loneliness	Mood swings	0.7467	0.022	33.9616	8.23E-253	6.37E-250
loneliness	Mothers age at death	-0.3212	0.0595	-5.4004	6.65E-08	5.15E-05
loneliness	Mothers age at death	-0.3031	0.0652	-4.6482	3.35E-06	0.0025929
loneliness	Mouth/teeth dental problems: Dentures	0.2609	0.0392	6.654	2.85E-11	2.21E-08
loneliness	Mouth/teeth dental problems: Loose teeth	0.3005	0.0604	4.9787	6.40E-07	0.00049536
loneliness	Mouth/teeth dental problems: None of the above	-0.3519	0.0358	-9.8328	8.13E-23	6.29E-20
loneliness	Mouth/teeth dental problems: Painful gums	0.3895	0.0722	5.3962	6.81E-08	5.27E-05
loneliness	Mouth/teeth dental problems: Toothache	0.3048	0.0687	4.4354	9.19E-06	0.00711306
loneliness	Nap during day	0.1755	0.0281	6.2423	4.31E-10	3.34E-07
loneliness	Nervous feelings	0.374	0.0321	11.6617	2.00E-31	1.55E-28
loneliness	Neuroticism	0.7233	0.0247	29.3281	4.54E-189	3.51E-186
loneliness	Noisy workplace	0.4651	0.0451	10.3039	6.77E-25	5.24E-22
loneliness	Non-cancer illness: angina	0.2984	0.0434	6.88	5.98E-12	4.63E-09
loneliness	Non-cancer illness: anxiety/panic attacks	0.5046	0.0886	5.6983	1.21E-08	9.37E-06
loneliness	Non-cancer illness: asthma	0.1603	0.0309	5.1844	2.17E-07	0.000167958
loneliness	Non-cancer illness: chronic obstructive airways disease/copd	0.3469	0.0805	4.3095	1.64E-05	0.0126936
loneliness	Non-cancer illness: depression	0.6273	0.0425	14.7458	3.27E-49	2.53E-46
loneliness	Non-cancer illness: diabetes	0.2491	0.0391	6.3647	1.96E-10	1.52E-07
loneliness	Non-cancer illness: emphysema/chronic bronchitis	0.4559	0.0832	5.4793	4.27E-08	3.30E-05
loneliness	Non-cancer illness: gastro-oesophageal reflux (gord) / gastric reflux	0.4093	0.0592	6.9112	4.81E-12	3.72E-09
loneliness	Non-cancer illness: hiatus hernia	0.2969	0.0652	4.5536	5.27E-06	0.00407898
loneliness	Non-cancer illness: hypothyroidism/myxoedema	0.1901	0.0392	4.8466	1.26E-06	0.00097524
loneliness	Non-cancer illness: osteoarthritis	0.3551	0.0493	7.2008	5.99E-13	4.64E-10
loneliness	Non-cancer illness: pulmonary embolism +/- dvt	0.34	0.0851	3.9957	6.45E-05	0.049923
loneliness	Number of cigarettes currently smoked daily (current cigarette smokers)	0.3363	0.0722	4.6566	3.21E-06	0.00248454
loneliness	Number of days/week of moderate physical activity 10+ minutes	0.1769	0.0388	4.5538	5.27E-06	0.00407898
loneliness	Number of depression episodes	0.4612	0.1103	4.1821	2.89E-05	0.0223686
loneliness	Number of full brothers	0.3386	0.0551	6.1411	8.19E-10	6.34E-07
loneliness	Number of full sisters	0.2204	0.0511	4.3121	1.62E-05	0.0125388
loneliness	Number of operations_ self-reported	0.2973	0.0414	7.1893	6.51E-13	5.04E-10
loneliness	Number of self-reported non-cancer illnesses	0.418	0.0323	12.9279	3.13E-38	2.42E-35
loneliness	Number of treatments/medications taken	0.3988	0.0305	13.0887	3.82E-39	2.96E-36

Supplementary Table 2e: Genetic correlations between loneliness and other phenotypes (pvalue_bonf, corrected for multiple comparisons using Bonferroni's method).

phenotype 1	phenotype 2	Rg	se	z	pvalue	pvalue_bonf
loneliness	Obesity class 1	0.2073	0.0322	6.4353	1.23E-10	9.52E-08
loneliness	Obesity class 2	0.2136	0.0396	5.3894	7.07E-08	5.47E-05
loneliness	Other eye problems	0.4437	0.0687	6.4606	1.04E-10	8.05E-08
loneliness	Other serious medical condition/disability diagnosed by doctor	0.4051	0.0378	10.7109	9.05E-27	7.00E-24
loneliness	Overall health rating	0.5673	0.0271	20.9181	3.66E-97	2.83E-94
loneliness	Overweight	0.2089	0.0355	5.8793	4.12E-09	3.19E-06
loneliness	PGC cross-disorder analysis	0.2922	0.0438	6.6666	2.62E-11	2.03E-08
loneliness	Pack years adult smoking as proportion of life span exposed to smoking	0.3934	0.0402	9.7799	1.37E-22	1.06E-19
loneliness	Pack years of smoking PREVIEW ONLY	0.3897	0.042	9.2682	1.89E-20	1.46E-17
loneliness	Pain type(s) experienced in last month: Back pain	0.4408	0.0336	13.1376	2.01E-39	1.56E-36
loneliness	Pain type(s) experienced in last month: Facial pain	0.5117	0.0796	6.4264	1.31E-10	1.01E-07
loneliness	Pain type(s) experienced in last month: Headache	0.3807	0.0304	12.517	6.03E-36	4.67E-33
loneliness	Pain type(s) experienced in last month: Hip pain	0.509	0.0395	12.8903	5.11E-38	3.96E-35
loneliness	Pain type(s) experienced in last month: Knee pain	0.3966	0.0331	11.9818	4.43E-33	3.43E-30
loneliness	Pain type(s) experienced in last month: Neck or shoulder pain	0.5031	0.0374	13.4662	2.47E-41	1.91E-38
loneliness	Pain type(s) experienced in last month: None of the above	-0.546	0.0276	-19.8085	2.51E-87	1.94E-84
loneliness	Pain type(s) experienced in last month: Pain all over the body	0.515	0.0645	7.9795	1.47E-15	1.14E-12
loneliness	Pain type(s) experienced in last month: Stomach or abdominal pain	0.5776	0.0413	13.9946	1.68E-44	1.30E-41
loneliness	Past tobacco smoking	-0.1529	0.0296	-5.1608	2.46E-07	0.000190404
loneliness	Peak expiratory flow (PEF)	-0.1554	0.032	-4.8546	1.21E-06	0.00093654
loneliness	Prospective memory result	0.2185	0.0491	4.4507	8.56E-06	0.00662544
loneliness	Qualifications: A levels/AS levels or equivalent	-0.3477	0.0275	-12.6563	1.03E-36	7.97E-34
loneliness	Qualifications: CSEs or equivalent	0.3811	0.0532	7.1618	7.97E-13	6.17E-10
loneliness	Qualifications: College or University degree	-0.313	0.0244	-12.8058	1.52E-37	1.18E-34
loneliness	Qualifications: NVQ or HND or HNC or equivalent	0.4196	0.0566	7.4195	1.18E-13	9.13E-11
loneliness	Qualifications: None of the above	0.3629	0.0269	13.4797	2.06E-41	1.59E-38
loneliness	Qualifications: O levels/GCSEs or equivalent	-0.3499	0.0345	-10.137	3.78E-24	2.93E-21
loneliness	Qualifications: Other professional qualifications eg: nursing_ teaching	-0.3324	0.0325	-10.233	1.41E-24	1.09E-21
loneliness	Risk taking	0.2127	0.0356	5.9841	2.18E-09	1.69E-06
loneliness	Schizophrenia	0.1822	0.0276	6.6102	3.84E-11	2.97E-08
loneliness	Seen a psychiatrist for nerves_ anxiety_ tension or depression	0.5529	0.0338	16.3355	5.52E-60	4.27E-57
loneliness	Seen doctor (GP) for nerves_ anxiety_ tension or depression	0.5944	0.0279	21.2723	2.05E-100	1.59E-97
loneliness	Sensitivity / hurt feelings	0.5416	0.0277	19.5251	6.72E-85	5.20E-82
loneliness	Shortness of breath walking on level ground	0.4927	0.0509	9.6742	3.88E-22	3.00E-19
loneliness	Sleep duration	-0.1899	0.033	-5.7596	8.43E-09	6.52E-06
loneliness	Sleeplessness / insomnia	0.419	0.0305	13.7405	5.81E-43	4.50E-40
loneliness	Smoking status: Current	0.371	0.0358	10.3564	3.91E-25	3.03E-22
loneliness	Smoking/smokers in household	0.4613	0.0687	6.7185	1.84E-11	1.42E-08
loneliness	Snoring	-0.1406	0.0347	-4.0503	5.12E-05	0.0396288
loneliness	Sodium in urine	0.2523	0.0309	8.1643	3.23E-16	2.50E-13
loneliness	Standing height	-0.0925	0.0209	-4.4364	9.15E-06	0.0070821
loneliness	Subjective well being	-0.6859	0.0478	-14.3458	1.13E-46	8.75E-44
loneliness	Suffer from nerves	0.5394	0.0324	16.6473	3.17E-62	2.45E-59
loneliness	Taking other prescription medications	0.4686	0.0331	14.1494	1.88E-45	1.46E-42
loneliness	Tense / highly strung	0.5348	0.0293	18.2747	1.32E-74	1.02E-71
loneliness	Time from waking to first cigarette	-0.453	0.0882	-5.1347	2.83E-07	0.000219042
loneliness	Time spent watching television (TV)	0.274	0.0301	9.0942	9.53E-20	7.38E-17

Supplementary Table 2f: Genetic correlations between loneliness and other phenotypes (pvalue_bonf, corrected for multiple comparisons using Bonferroni's method).

phenotype 1	phenotype 2	Rg	se	z	pvalue	pvalue_bonf
loneliness	Tinnitus: Yes_ but not now_ but have in the past	0.3362	0.0823	4.0866	4.38E-05	0.0339012
loneliness	Townsend deprivation index at recruitment	0.438	0.0361	12.118	8.48E-34	6.56E-31
loneliness	Transport type for commuting to job workplace: Cycle	-0.2208	0.0527	-4.1899	2.79E-05	0.0215946
loneliness	Triglycerides	0.1566	0.0366	4.2741	1.92E-05	0.0148608
loneliness	Trunk fat mass	0.2058	0.023	8.9499	3.56E-19	2.76E-16
loneliness	Trunk fat percentage	0.2106	0.024	8.7824	1.60E-18	1.24E-15
loneliness	Types of physical activity in last 4 weeks: Light DIY	-0.169	0.0372	-4.5393	5.64E-06	0.00436536
loneliness	Types of physical activity in last 4 weeks: None of the above	0.3774	0.0448	8.4199	3.77E-17	2.92E-14
loneliness	Types of physical activity in last 4 weeks: Other exercises	-0.3204	0.0357	-8.9621	3.18E-19	2.46E-16
loneliness	Types of physical activity in last 4 weeks: Strenuous sports	-0.2754	0.0448	-6.1479	7.85E-10	6.08E-07
loneliness	Types of physical activity in last 4 weeks: Walking for pleasure	-0.3467	0.0426	-8.1414	3.91E-16	3.03E-13
loneliness	Types of transport used (excluding work): Car/motor vehicle	-0.1898	0.0406	-4.6763	2.92E-06	0.00226008
loneliness	Types of transport used (excluding work): Cycle	-0.2021	0.0438	-4.6176	3.88E-06	0.00300312
loneliness	Usual walking pace	-0.2888	0.0293	-9.8676	5.76E-23	4.46E-20
loneliness	Vascular/heart problems diagnosed by doctor: Angina	0.2787	0.0434	6.425	1.32E-10	1.02E-07
loneliness	Vascular/heart problems diagnosed by doctor: Heart attack	0.2179	0.0531	4.1003	4.13E-05	0.0319662
loneliness	Vascular/heart problems diagnosed by doctor: None of the above	-0.1418	0.0306	-4.6284	3.69E-06	0.00285606
loneliness	Waist circumference	0.2574	0.0231	11.1255	9.43E-29	7.30E-26
loneliness	Waist circumference	0.198	0.0293	6.7513	1.47E-11	1.14E-08
loneliness	Waist-to-hip ratio	0.2089	0.0328	6.3594	2.03E-10	1.57E-07
loneliness	Weight	0.1869	0.0224	8.3474	6.98E-17	5.40E-14
loneliness	Weight change compared with 1 year ago	0.5333	0.0704	7.5795	3.47E-14	2.69E-11
loneliness	Wheeze or whistling in the chest in last year	0.4693	0.0327	14.3354	1.32E-46	1.02E-43
loneliness	Whole body fat mass	0.2263	0.0232	9.7579	1.71E-22	1.32E-19
loneliness	Why stopped smoking: Health precaution	-0.2909	0.0585	-4.9749	6.53E-07	0.000505422
loneliness	Work/job satisfaction	0.4355	0.0598	7.2827	3.27E-13	2.53E-10
loneliness	Worrier / anxious feelings	0.3994	0.0306	13.0694	4.92E-39	3.81E-36
loneliness	Worry too long after embarrassment	0.3627	0.0341	10.6367	2.01E-26	1.56E-23
loneliness	Years of schooling (proxy cognitive performance)	-0.3716	0.0408	-9.1044	8.67E-20	6.71E-17
loneliness	Years of schooling 2013	-0.3514	0.0391	-8.9868	2.55E-19	1.97E-16
loneliness	Years of schooling 2016	-0.313	0.0241	-12.9903	1.39E-38	1.08E-35

Supplementary Table 3: Impact of biological sex on the association between grey matter volumes and loneliness. Network-level posterior parameters for women (0) and men (1)

	mean	hpd_5%	hpd_95%
sigma_nets_Cont[0]	0.034	0.003	0.07
sigma_nets_Cont[1]	0.059	0.008	0.103
sigma_nets_Default[0]	0.081	0.033	0.125
sigma_nets_Default[1]	0.083	0.045	0.126
sigma_nets_DorsAttn[0]	0.055	0.004	0.097
sigma_nets_DorsAttn[1]	0.059	0.003	0.113
sigma_nets_Limbic[0]	0.08	0.004	0.158
sigma_nets_Limbic[1]	0.151	0.004	0.284
sigma_nets_SalVentAttn[0]	0.029	0.001	0.069
sigma_nets_SalVentAttn[1]	0.041	0.003	0.075
sigma_nets_SomMot[0]	0.025	0	0.063
sigma_nets_SomMot[1]	0.098	0.02	0.16
sigma_nets_Vis[0]	0.033	0.003	0.059
sigma_nets_Vis[1]	0.039	0.002	0.083

Supplementary Table 3 Note: Cont: frontoparietal control network, Default: default network, DorsAttn: dorsal attention network, Limbic: limbic network, SalVentAttn: salience network, SomMot: somatomotor network, Vis: visual network.

Supplementary Figure 1: Loneliness-related structural associations (A) and means of posterior parameter by network (B) in women and men. One Bayesian hierarchical model was carried out in our UK Biobank sample (n=38,701). Boxplots bounds are based on interquartile range from 25%-75% (IQR), centre shows mean, and whiskers show distance indicating 1.5 of IQR in both directions.

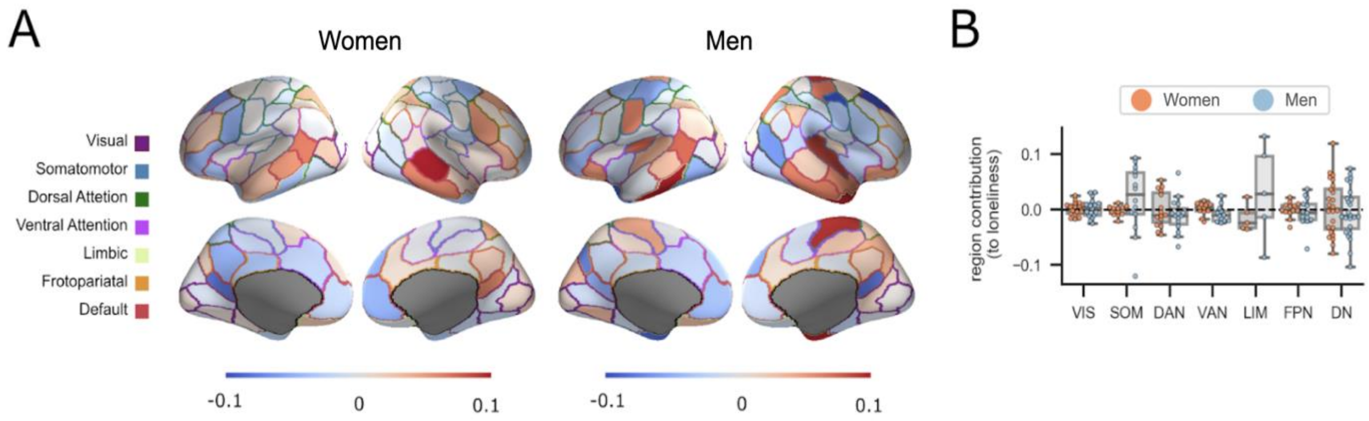

Supplementary Figure 2: Functional connectivity modes in men and women indicating deviation in loneliness

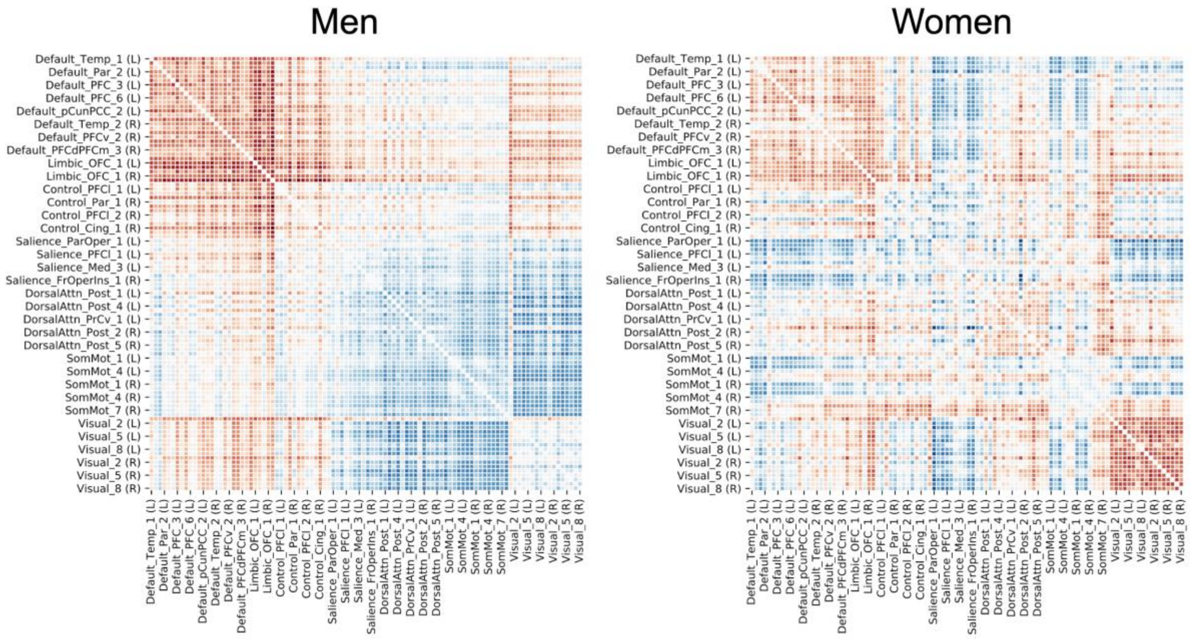

Supplementary Table 4: White matter tract associations with loneliness

	rho_pearson	rho_pearson_pvalues	rho_pearson_lower	rho_pearson_higher	rho_spearman	rho_spearman_pvalues	rho_pearson_pvalues_BONF0.001
fornix	0.060425487	6.35E-11	0.045682305	0.07605236	0.061374742	3.18E-11	TRUE
fornix cres+stria terminalis (L)	0.04702303	3.70E-07	0.031226124	0.061730809	0.047717263	2.48E-07	TRUE
fornix cres+stria terminalis (R)	0.042968632	3.40E-06	0.026864768	0.056504565	0.044286037	1.69E-06	TRUE
anterior corona radiata (L)	0.036139001	9.38E-05	0.020297436	0.05086813	0.032103004	0.000520987	FALSE
body of corpus callosum	0.034469943	0.000194777	0.019869653	0.050142623	0.036113154	9.48E-05	FALSE
anterior corona radiata (R)	0.02968374	0.001336007	0.014602076	0.04488316	0.026122252	0.00475654	FALSE
tapetum (R)	0.028333168	0.002197864	0.011388015	0.043223191	0.02993958	0.001213051	FALSE
external capsule (R)	0.027729528	0.002727927	0.009768096	0.040974991	0.025780404	0.00533433	FALSE
genu of corpus callosum	0.024025045	0.009421775	0.011060315	0.039870852	0.025553489	0.005752104	FALSE
superior fronto-occipital fasciculus (L)	0.022773941	0.01385052	0.008219155	0.034297766	0.015333435	0.097534742	FALSE
external capsule (L)	0.020241356	0.028715677	0.004796253	0.031784552	0.021717056	0.018931618	FALSE
posterior thalamic radiation (R)	0.018189519	0.049344966	0.003004463	0.035319678	0.021394669	0.020776161	FALSE
posterior thalamic radiation (L)	0.01701236	0.066007404	0.001260433	0.033555985	0.020351242	0.027860496	FALSE
cingulum hippocampus (L)	0.014091425	0.12783388	-0.001569021	0.031014388	0.019509174	0.035011114	FALSE
superior corona radiata (R)	0.013605641	0.1415072	-0.000702406	0.027823215	0.014056131	0.128791356	FALSE
tapetum (L)	0.013256463	0.152010342	-0.002110732	0.026872783	0.011626719	0.2089902	FALSE
superior fronto-occipital fasciculus (R)	0.013251854	0.152152846	-0.001209497	0.029761505	0.008273994	0.371296709	FALSE
superior corona radiata (L)	0.013135276	0.15579112	-0.002250892	0.028174313	0.013668352	0.139681395	FALSE
cingulum cingulate gyrus (R)	0.01116535	0.227628707	-0.003278867	0.023967617	0.010267155	0.267245403	FALSE
anterior limb of internal capsule (R)	0.008860277	0.338365224	-0.00717379	0.024579883	0.008757753	0.343984586	FALSE
uncinate fasciculus (L)	0.008173797	0.377117487	-0.008077486	0.022702748	0.008142092	0.378970954	FALSE
cerebral peduncle (L)	0.007825089	0.397809915	-0.007496066	0.022293581	0.013663525	0.139821281	FALSE
pontine crossing tract	0.004731291	0.609187112	-0.011082473	0.017908171	0.006133333	0.507501777	FALSE
anterior limb of internal capsule (L)	0.004310325	0.641396799	-0.012139401	0.01961359	0.004831336	0.601639339	FALSE
splenium of corpus callosum	0.003490381	0.706063566	-0.011193877	0.016779527	0.009567252	0.301231497	FALSE
cingulum cingulate gyrus (L)	0.003115924	0.736353237	-0.010984853	0.017459736	0.00570013	0.537945692	FALSE
inferior cerebellar peduncle (R)	0.00241885	0.79381106	-0.011458598	0.017851889	-0.001919944	0.835653502	FALSE
inferior cerebellar peduncle (L)	0.000880142	0.924233632	-0.013818667	0.015964592	-0.002226986	0.80983928	FALSE
posterior corona radiata (L)	0.000736892	0.936536647	-0.013910369	0.016043975	0.001197583	0.897038951	FALSE
posterior corona radiata (R)	0.000524364	0.954816615	-0.01433956	0.013341008	0.004823613	0.602220492	FALSE
sagittal stratum (L)	-0.000459143	0.960431697	-0.016170723	0.013928627	0.00080264	0.930887845	FALSE
superior longitudinal fasciculus (L)	-0.001578184	0.864594953	-0.017363584	0.011702504	0.00098149	0.915540169	FALSE
uncinate fasciculus (R)	-0.002505072	0.78663601	-0.017963065	0.010497259	-0.003195418	0.729886788	FALSE
retrolenticular part of internal capsule (L)	-0.002812133	0.761234091	-0.015029839	0.015046665	-0.00159837	0.862880084	FALSE
cingulum hippocampus (R)	-0.004304049	0.641882356	-0.020894043	0.011014433	-0.001173753	0.899076617	FALSE
sagittal stratum (R)	-0.005072137	0.583647754	-0.019843723	0.011086925	-0.003954667	0.669149059	FALSE
superior longitudinal fasciculus (R)	-0.006533551	0.480201346	-0.022770473	0.008508833	-0.001673517	0.856501761	FALSE
middle cerebellar peduncle	-0.009558547	0.301671566	-0.02457115	0.006578967	-0.012875772	0.164126465	FALSE
posterior limb of internal capsule (L)	-0.011911547	0.19805012	-0.025500787	0.000679234	-0.011835496	0.200929348	FALSE
cerebral peduncle (R)	-0.013809224	0.135646107	-0.029012537	0.001362692	-0.010604073	0.251862784	FALSE
medial lemniscus (L)	-0.015916552	0.08544267	-0.033155191	-0.000527612	-0.012898433	0.163385459	FALSE
retrolenticular part of internal capsule (R)	-0.016426819	0.07588294	-0.029492736	0.001504657	-0.016163867	0.080694228	FALSE
posterior limb of internal capsule (R)	-0.017812054	0.054254051	-0.031455329	-0.004470121	-0.017658179	0.056368993	FALSE
corticospinal tract (L)	-0.023496294	0.0111106	-0.037922844	-0.008106271	-0.021468698	0.02033928	FALSE
medial lemniscus (R)	-0.027346163	0.003122701	-0.040254098	-0.013024722	-0.026604232	0.004037947	FALSE
superior cerebellar peduncle (L)	-0.033446716	0.000300305	-0.045214168	-0.018837831	-0.032269812	0.000487075	FALSE
superior cerebellar peduncle (R)	-0.042930766	3.47E-06	-0.056102778	-0.027819838	-0.040520535	1.19E-05	TRUE
corticospinal tract (R)	-0.046049475	6.41E-07	-0.063137722	-0.029725105	-0.043908268	2.07E-06	TRUE

Supplementary Table 5: White matter tract associations with loneliness in men

	rho_pearson	rho_pearson_pvalues	rho_pearson_lower	rho_pearson_higher
Mean FA in fornix on FA skeleton	0.05274587	0.000160382	0.030707465	0.071956762
Mean FA in fornix cres+stria terminalis on FA skeleton (left)	0.051998025	0.000198569	0.02805862	0.075054907
Mean FA in anterior corona radiata on FA skeleton (left)	0.035708642	0.010640318	0.014703969	0.057828784
Mean FA in external capsule on FA skeleton (right)	0.032625542	0.019614965	0.010131273	0.055983413
Mean FA in fornix cres+stria terminalis on FA skeleton (right)	0.026445727	0.058566782	-0.000729385	0.047830854
Mean FA in anterior corona radiata on FA skeleton (right)	0.025687905	0.066178469	0.004273709	0.051734147
Mean FA in cerebral peduncle on FA skeleton (left)	0.024651178	0.077893307	0.002284576	0.046890964
Mean FA in uncinate fasciculus on FA skeleton (left)	0.023979748	0.08634373	0.003114997	0.043379459
Mean FA in external capsule on FA skeleton (left)	0.022237669	0.111748187	-0.000974234	0.045865207
Mean FA in genu of corpus callosum on FA skeleton	0.017892281	0.200701007	-0.006112361	0.042532772
Mean FA in anterior limb of internal capsule on FA skeleton (right)	0.017766287	0.203890526	-0.007653142	0.039155472
Mean FA in tapetum on FA skeleton (right)	0.016987141	0.224436004	-0.009542728	0.035091501
Mean FA in superior fronto-occipital fasciculus on FA skeleton (left)	0.016872377	0.22758303	-0.00598509	0.041907331
Mean FA in cingulum cingulate gyrus on FA skeleton (right)	0.015859175	0.256735427	-0.00183044	0.042100357
Mean FA in anterior limb of internal capsule on FA skeleton (left)	0.013919993	0.319517727	-0.008688279	0.033907381
Mean FA in cingulum hippocampus on FA skeleton (left)	0.012419997	0.374447907	-0.006589819	0.035778052
Mean FA in body of corpus callosum on FA skeleton	0.011367172	0.416287767	-0.009707762	0.034546445
Mean FA in posterior thalamic radiation on FA skeleton (right)	0.010599999	0.44844266	-0.012693334	0.034428278
Mean FA in uncinate fasciculus on FA skeleton (right)	0.00958532	0.493060073	-0.011099093	0.034230923
Mean FA in superior fronto-occipital fasciculus on FA skeleton (right)	0.009407926	0.501097543	-0.012128254	0.032723366
Mean FA in middle cerebellar peduncle on FA skeleton	0.005503924	0.69388995	-0.017850503	0.030039892
Mean FA in tapetum on FA skeleton (left)	0.00519285	0.710386803	-0.016220633	0.026820188
Mean FA in cingulum cingulate gyrus on FA skeleton (left)	0.004562836	0.744209202	-0.010795038	0.029556278
Mean FA in cerebral peduncle on FA skeleton (right)	0.001534062	0.912648155	-0.020587876	0.021272488
Mean FA in cingulum hippocampus on FA skeleton (right)	-3.98E-05	0.997729154	-0.024020367	0.028303807
Mean FA in superior corona radiata on FA skeleton (right)	-0.002566977	0.854357397	-0.025948127	0.018782946
Mean FA in sagittal stratum on FA skeleton (right)	-0.002802764	0.841150176	-0.032994125	0.022356369
Mean FA in sagittal stratum on FA skeleton (left)	-0.003146396	0.821982901	-0.028393268	0.018911156
Mean FA in superior corona radiata on FA skeleton (left)	-0.003203262	0.81882084	-0.024779299	0.023655923
Mean FA in posterior thalamic radiation on FA skeleton (left)	-0.00350768	0.801945133	-0.028239362	0.018157763
Mean FA in splenium of corpus callosum on FA skeleton	-0.005528243	0.692606236	-0.032060328	0.019724315
Mean FA in posterior limb of internal capsule on FA skeleton (left)	-0.006844251	0.624537642	-0.030408503	0.012494102
Mean FA in inferior cerebellar peduncle on FA skeleton (left)	-0.006863431	0.623567116	-0.022999077	0.015291958
Mean FA in inferior cerebellar peduncle on FA skeleton (right)	-0.007931106	0.570610695	-0.027669534	0.01429176
Mean FA in retrolenticular part of internal capsule on FA skeleton (left)	-0.008413586	0.547403401	-0.027110998	0.012884858
Mean FA in retrolenticular part of internal capsule on FA skeleton (right)	-0.009175732	0.511721931	-0.027332815	0.013336494
Mean FA in posterior corona radiata on FA skeleton (left)	-0.014268407	0.307554095	-0.037639341	0.00975533
Mean FA in pontine crossing tract on FA skeleton	-0.014336581	0.305248283	-0.038449156	0.00943135
Mean FA in superior longitudinal fasciculus on FA skeleton (right)	-0.014426813	0.302214129	-0.03573612	0.010998869
Mean FA in posterior corona radiata on FA skeleton (right)	-0.016986084	0.224464837	-0.036520327	0.008739183
Mean FA in superior longitudinal fasciculus on FA skeleton (left)	-0.018744001	0.180090108	-0.037791625	0.005820741
Mean FA in posterior limb of internal capsule on FA skeleton (right)	-0.019433423	0.16459239	-0.039460433	0.000498642
Mean FA in medial lemniscus on FA skeleton (left)	-0.029977641	0.032021256	-0.052081986	-0.010092936
Mean FA in corticospinal tract on FA skeleton (left)	-0.040401046	0.003849871	-0.06946072	-0.016961692
Mean FA in superior cerebellar peduncle on FA skeleton (left)	-0.043012578	0.002089555	-0.067338614	-0.022182104
Mean FA in medial lemniscus on FA skeleton (right)	-0.046887316	0.000794517	-0.066844783	-0.024974768
Mean FA in corticospinal tract on FA skeleton (right)	-0.049317503	0.00041748	-0.074334136	-0.028117092
Mean FA in superior cerebellar peduncle on FA skeleton (right)	-0.059002569	2.41E-05	-0.084065885	-0.03440644

Supplementary Table 6: White matter tract associations with loneliness in women

	rho_pearson	rho_pearson_pvalues	rho_pearson_lower	rho_pearson_higher
Mean FA in body of corpus callosum on FA skeleton	0.033366121	0.006869619	0.012301629	0.052808617
Mean FA in fornix on FA skeleton	0.02846038	0.021138911	0.010906648	0.04647622
Mean FA in fornix cres+stria terminalis on FA skeleton (right)	0.023647204	0.05543229	0.001998902	0.044255666
Mean FA in cingulum hippocampus on FA skeleton (left)	0.019985535	0.105489234	0.003108789	0.040792262
Mean FA in tapetum on FA skeleton (right)	0.01325047	0.28317579	-0.004657575	0.030369685
Mean FA in genu of corpus callosum on FA skeleton	0.013179108	0.28577678	-0.005462297	0.030305225
Mean FA in cingulum cingulate gyrus on FA skeleton (right)	0.012191607	0.32342505	-0.006648595	0.028117099
Mean FA in pontine crossing tract on FA skeleton	0.011539388	0.349986894	-0.008164651	0.02886433
Mean FA in anterior corona radiata on FA skeleton (left)	0.011257689	0.361875365	-0.009291676	0.027332452
Mean FA in superior fronto-occipital fasciculus on FA skeleton (left)	0.010785202	0.382375954	-0.005636489	0.026625655
Mean FA in anterior corona radiata on FA skeleton (right)	0.009980531	0.418888702	-0.006538594	0.031260838
Mean FA in posterior thalamic radiation on FA skeleton (left)	0.009727415	0.430784932	-0.008867959	0.028321112
Mean FA in cingulum cingulate gyrus on FA skeleton (left)	0.009688359	0.432637813	-0.012839291	0.029061011
Mean FA in posterior corona radiata on FA skeleton (right)	0.008818912	0.475063868	-0.012984731	0.028764434
Mean FA in superior corona radiata on FA skeleton (left)	0.008660611	0.483026929	-0.012574021	0.028288094
Mean FA in tapetum on FA skeleton (left)	0.008563857	0.487929435	-0.014474248	0.027101368
Mean FA in inferior cerebellar peduncle on FA skeleton (right)	0.007500148	0.543551599	-0.010342747	0.02707971
Mean FA in superior corona radiata on FA skeleton (right)	0.007144125	0.562848723	-0.015164596	0.028046913
Mean FA in fornix cres+stria terminalis on FA skeleton (left)	0.006467076	0.600432421	-0.016048756	0.026180717
Mean FA in splenium of corpus callosum on FA skeleton	0.006085693	0.622090748	-0.018185601	0.022974183
Mean FA in external capsule on FA skeleton (right)	0.005887538	0.633475933	-0.010708177	0.024095941
Mean FA in posterior corona radiata on FA skeleton (left)	0.005239474	0.671309255	-0.011578771	0.024445124
Mean FA in inferior cerebellar peduncle on FA skeleton (left)	0.004513067	0.714724428	-0.014843703	0.027115456
Mean FA in anterior limb of internal capsule on FA skeleton (right)	0.004259416	0.730113329	-0.016522216	0.024380644
Mean FA in medial lemniscus on FA skeleton (left)	0.003604996	0.770307824	-0.013403421	0.026110887
Mean FA in external capsule on FA skeleton (left)	0.003261474	0.791664404	-0.015943793	0.019887291
Mean FA in anterior limb of internal capsule on FA skeleton (left)	0.003195584	0.795779238	-0.01766629	0.024035331
Mean FA in corticospinal tract on FA skeleton (left)	0.002387667	0.846662024	-0.020773576	0.023999669
Mean FA in cingulum hippocampus on FA skeleton (right)	0.001849155	0.880950346	-0.016053967	0.018131235
Mean FA in superior longitudinal fasciculus on FA skeleton (left)	0.001337319	0.913749365	-0.017853795	0.020136264
Mean FA in superior fronto-occipital fasciculus on FA skeleton (right)	0.00118376	0.923620942	-0.017142534	0.022520655
Mean FA in medial lemniscus on FA skeleton (right)	0.000611714	0.960486403	-0.020373154	0.021070464
Mean FA in posterior thalamic radiation on FA skeleton (right)	-0.000281333	0.981821457	-0.022151209	0.018104489
Mean FA in superior longitudinal fasciculus on FA skeleton (right)	-0.005538799	0.653723916	-0.025773727	0.010788035
Mean FA in uncinate fasciculus on FA skeleton (left)	-0.006313525	0.609111469	-0.018426032	0.008939858
Mean FA in uncinate fasciculus on FA skeleton (right)	-0.006767234	0.583630382	-0.023553315	0.015952285
Mean FA in retrolenticular part of internal capsule on FA skeleton (left)	-0.007260338	0.556513587	-0.027644923	0.012043385
Mean FA in posterior limb of internal capsule on FA skeleton (right)	-0.00933095	0.449806895	-0.029692963	0.012766653
Mean FA in cerebral peduncle on FA skeleton (left)	-0.009401	0.446411742	-0.033448981	0.013236881
Mean FA in posterior limb of internal capsule on FA skeleton (left)	-0.011688285	0.343804324	-0.030520081	0.006793725
Mean FA in sagittal stratum on FA skeleton (left)	-0.013434992	0.276524884	-0.033009516	0.008512162
Mean FA in middle cerebellar peduncle on FA skeleton	-0.013849171	0.261986445	-0.032888098	0.010480047
Mean FA in superior cerebellar peduncle on FA skeleton (left)	-0.014517504	0.239656691	-0.035808493	0.001515818
Mean FA in retrolenticular part of internal capsule on FA skeleton (right)	-0.015306125	0.21507638	-0.031263342	0.00380007
Mean FA in superior cerebellar peduncle on FA skeleton (right)	-0.01802982	0.144189193	-0.035304106	-0.002048382
Mean FA in sagittal stratum on FA skeleton (right)	-0.020226548	0.101352732	-0.042148495	0.001788266
Mean FA in corticospinal tract on FA skeleton (right)	-0.020796648	0.092081591	-0.038779764	-0.001717046
Mean FA in cerebral peduncle on FA skeleton (right)	-0.022174567	0.072469799	-0.046239181	0.000491639

Supplementary Table 7: Sub-analysis accounting for depression and anxiety diagnoses: Network-level posterior parameters

	mean	hpd_5%	hpd_95%
sigma_nets_Cont[0]	0.072	0.003	0.127
sigma_nets_Default[0]	0.084	0.029	0.124
sigma_nets_DorsAttn[0]	0.04	0.003	0.076
sigma_nets_Limbic[0]	0.07	0.004	0.155
sigma_nets_SalVentAttn[0]	0.06	0.003	0.111
sigma_nets_SomMot[0]	0.057	0.006	0.107
sigma_nets_Vis[0]	0.057	0.004	0.107

Supplementary Table 8: Sub-analysis accounting for depression and anxiety diagnoses: Region-level posterior parameters

	mean	hpd_5%	hpd_95%
RH_Cont_PFCmp_1	0.139	0.007	0.275
LH_Default_Temp_2	0.147	0.017	0.293
LH_Default_Par_1	0.169	0.043	0.277
LH_Default_pCunPCC_1	-0.185	-0.296	-0.073
RH_Default_Temp_1	0.323	0.117	0.469
RH_Default_Temp_3	0.266	0.108	0.412
RH_DorsAttn_Post_1	-0.28	-0.415	-0.121
RH_DorsAttn_Post_4	0.149	0.049	0.266
LH_SalVentAttn_Med_1	-0.149	-0.265	-0.004
RH_SomMot_3	-0.157	-0.275	-0.039
RH_SomMot_5	-0.117	-0.219	-0.009
LH_Vis_3	0.122	0.015	0.255
LH_Vis_6	-0.125	-0.235	-0.019
RH_Vis_3	-0.185	-0.344	-0.053

Supplementary Figure 3: Sub-analysis accounting for depression and anxiety diagnoses: Dominant functional connectivity mode

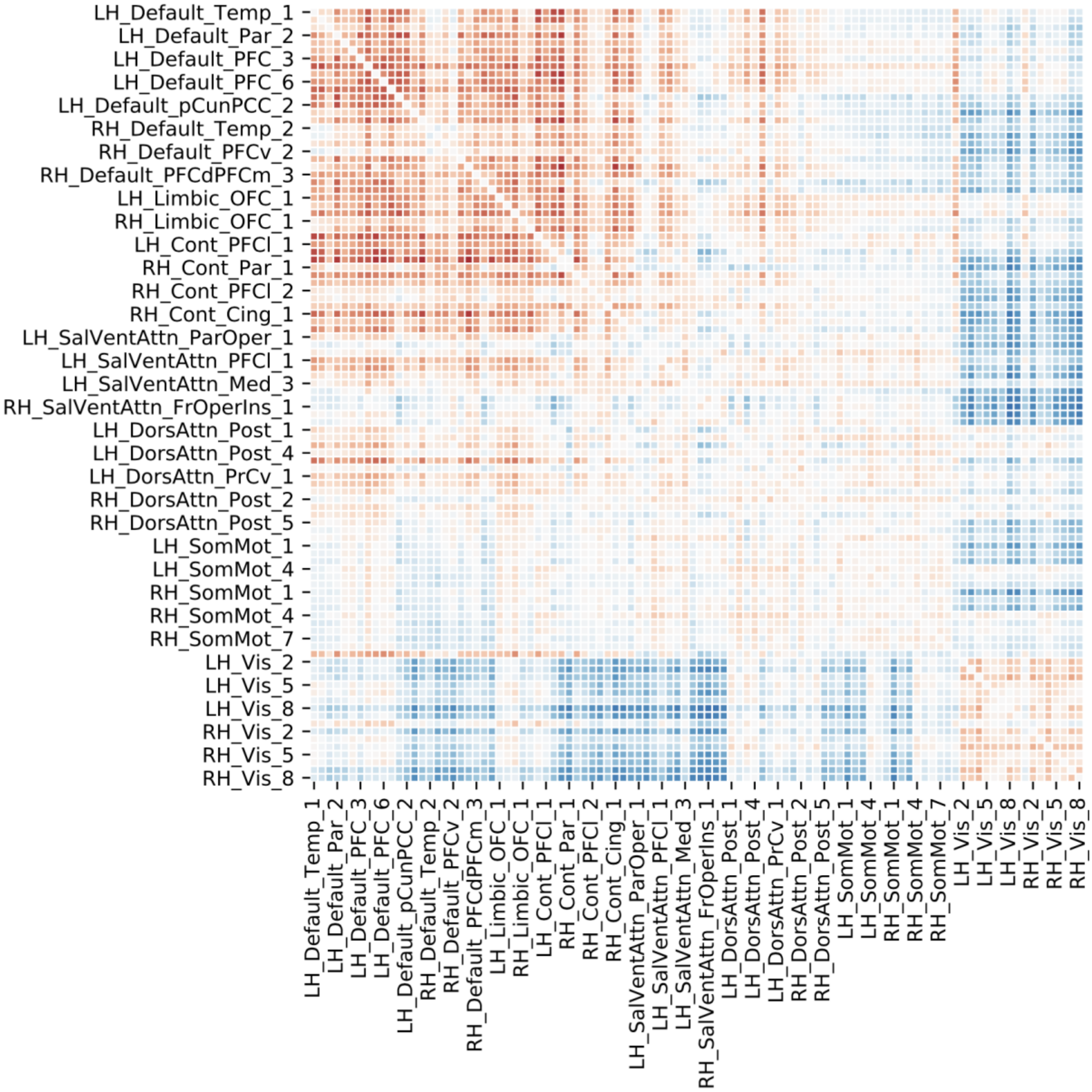

Supplementary Table 9: Sub-analysis accounting for depression and anxiety diagnoses: White matter tract associations with loneliness

	rho_pearson	rho_pearson_pvalues	rho_spearman	rho_spearman_pvalues
Mean FA in fornix on FA skeleton	0.060456585	6.21E-11	0.061341868	3.26E-11
Mean FA in fornix cres+stria terminalis on FA skeleton (left)	0.047110595	3.52E-07	0.047845151	2.31E-07
Mean FA in fornix cres+stria terminalis on FA skeleton (right)	0.043170078	3.06E-06	0.044625547	1.40E-06
Mean FA in anterior corona radiata on FA skeleton (left)	0.036273325	8.83E-05	0.032235617	0.000493854
Mean FA in body of corpus callosum on FA skeleton	0.034596453	0.000184476	0.036260045	8.88E-05
Mean FA in anterior corona radiata on FA skeleton (right)	0.02984755	0.001256024	0.026274223	0.004518362
Mean FA in tapetum on FA skeleton (right)	0.028509598	0.002061817	0.0300852	0.00114782
Mean FA in external capsule on FA skeleton (right)	0.027794589	0.002665645	0.025792599	0.005312675
Mean FA in genu of corpus callosum on FA skeleton	0.0242691	0.00872256	0.025728071	0.005428173
Mean FA in superior fronto-occipital fasciculus on FA skeleton (left)	0.022784031	0.01380847	0.015340548	0.097379397
Mean FA in external capsule on FA skeleton (left)	0.020276362	0.028440824	0.021738955	0.018811684
Mean FA in posterior thalamic radiation on FA skeleton (right)	0.018374633	0.047077084	0.021556752	0.019830093
Mean FA in posterior thalamic radiation on FA skeleton (left)	0.017224474	0.06270209	0.020529228	0.026521907
Mean FA in cingulum hippocampus on FA skeleton (left)	0.014086861	0.127957403	0.019486564	0.035222943
Mean FA in superior corona radiata on FA skeleton (right)	0.013673383	0.139535703	0.014119846	0.1270669
Mean FA in tapetum on FA skeleton (left)	0.01336482	0.14868936	0.011641352	0.208417691
Mean FA in superior fronto-occipital fasciculus on FA skeleton (right)	0.01319675	0.153864424	0.00822227	0.374294539
Mean FA in superior corona radiata on FA skeleton (left)	0.013174515	0.154559211	0.013695891	0.138885363
Mean FA in cingulum cingulate gyrus on FA skeleton (right)	0.011310711	0.221632783	0.010259432	0.267605426
Mean FA in anterior limb of internal capsule on FA skeleton (right)	0.00896065	0.332921246	0.008865571	0.338076699
Mean FA in uncinate fasciculus on FA skeleton (left)	0.008223127	0.374244735	0.008192394	0.376032894
Mean FA in cerebral peduncle on FA skeleton (left)	0.007789009	0.399989305	0.013641982	0.140446932
Mean FA in pontine crossing tract on FA skeleton	0.004801115	0.603914902	0.006134654	0.507410342
Mean FA in anterior limb of internal capsule on FA skeleton (left)	0.004536058	0.624036071	0.005074003	0.583509318
Mean FA in splenium of corpus callosum on FA skeleton	0.003650985	0.693210443	0.00955521	0.301840362
Mean FA in cingulum cingulate gyrus on FA skeleton (left)	0.003296261	0.721711289	0.005793925	0.531277209
Mean FA in inferior cerebellar peduncle on FA skeleton (right)	0.002434852	0.792478091	-0.001895758	0.837694788
Mean FA in inferior cerebellar peduncle on FA skeleton (left)	0.000933374	0.919666323	-0.002156615	0.815738501
Mean FA in posterior corona radiata on FA skeleton (left)	0.000858382	0.926101427	0.001215671	0.895492689
Mean FA in posterior corona radiata on FA skeleton (right)	0.000604044	0.947959901	0.004776208	0.605793227
Mean FA in sagittal stratum on FA skeleton (left)	-0.000307265	0.973514286	0.000986113	0.915143789
Mean FA in superior longitudinal fasciculus on FA skeleton (left)	-0.001494088	0.871746008	0.001043528	0.910223496
Mean FA in uncinate fasciculus on FA skeleton (right)	-0.002499525	0.787097061	-0.003208781	0.728801644
Mean FA in retrolenticular part of internal capsule on FA skeleton (left)	-0.002862281	0.757109081	-0.001739925	0.850872978
Mean FA in cingulum hippocampus on FA skeleton (right)	-0.004192253	0.650557028	-0.001021325	0.912125902
Mean FA in sagittal stratum on FA skeleton (right)	-0.004963888	0.591704637	-0.003923876	0.671573763
Mean FA in superior longitudinal fasciculus on FA skeleton (right)	-0.006424065	0.487589313	-0.001615848	0.861395739
Mean FA in middle cerebellar peduncle on FA skeleton	-0.009569296	0.301128211	-0.012877398	0.164073214
Mean FA in posterior limb of internal capsule on FA skeleton (left)	-0.011832257	0.201052641	-0.011739364	0.204612361
Mean FA in corticospinal tract on FA skeleton (left)	-0.023438195	0.011311658	-0.021404018	0.020720541
Mean FA in medial lemniscus on FA skeleton (right)	-0.027260591	0.003217644	-0.026510793	0.004169035
Mean FA in superior cerebellar peduncle on FA skeleton (left)	-0.033334635	0.000314667	-0.032175661	0.000505954
Mean FA in superior cerebellar peduncle on FA skeleton (right)	-0.042826132	3.66E-06	-0.040490252	1.20E-05
Mean FA in corticospinal tract on FA skeleton (right)	-0.046019742	6.52E-07	-0.04382687	2.16E-06

Supplementary Table 10: Sub-analysis accounting for neuroticism: Network-level posterior parameters

	mean	hpd_5%	hpd_95%
sigma_nets_Cont[0]	0.024	0.001	0.047
sigma_nets_Default[0]	0.051	0.018	0.079
sigma_nets_DorsAttn[0]	0.04	0.003	0.067
sigma_nets_Limbic[0]	0.073	0.003	0.158
sigma_nets_SalVentAttn[0]	0.041	0.003	0.081
sigma_nets_SomMot[0]	0.032	0.002	0.061
sigma_nets_Vis[0]	0.033	0.003	0.062

Supplementary Table 11: Sub-analysis accounting for neuroticism: Region-level posterior parameters

	mean	hpd_5%	hpd_95%
RH_Cont_Par_1	0.118	0.031	0.218
LH_Default_Par_1	0.131	0.015	0.262
LH_Default_pCunPCC_1	-0.206	-0.305	-0.085
RH_Default_Temp_1	0.232	0.076	0.372
RH_Default_Temp_3	0.216	0.06	0.364
RH_Default_pCunPCC_2	0.138	0.01	0.287
RH_DorsAttn_Post_1	-0.239	-0.394	-0.087
RH_DorsAttn_Post_4	0.139	0.025	0.243
LH_Limbic_OFC_1	0.177	0.006	0.365
LH_Limbic_TempPole_1	-0.186	-0.344	-0.005
LH_SalVentAttn_Med_1	-0.209	-0.363	-0.06
RH_SomMot_3	-0.128	-0.249	-0.024
LH_Vis_3	0.16	0.031	0.29

Supplementary Figure 4: Sub-analysis accounting for neuroticism:
Dominant functional connectivity mode

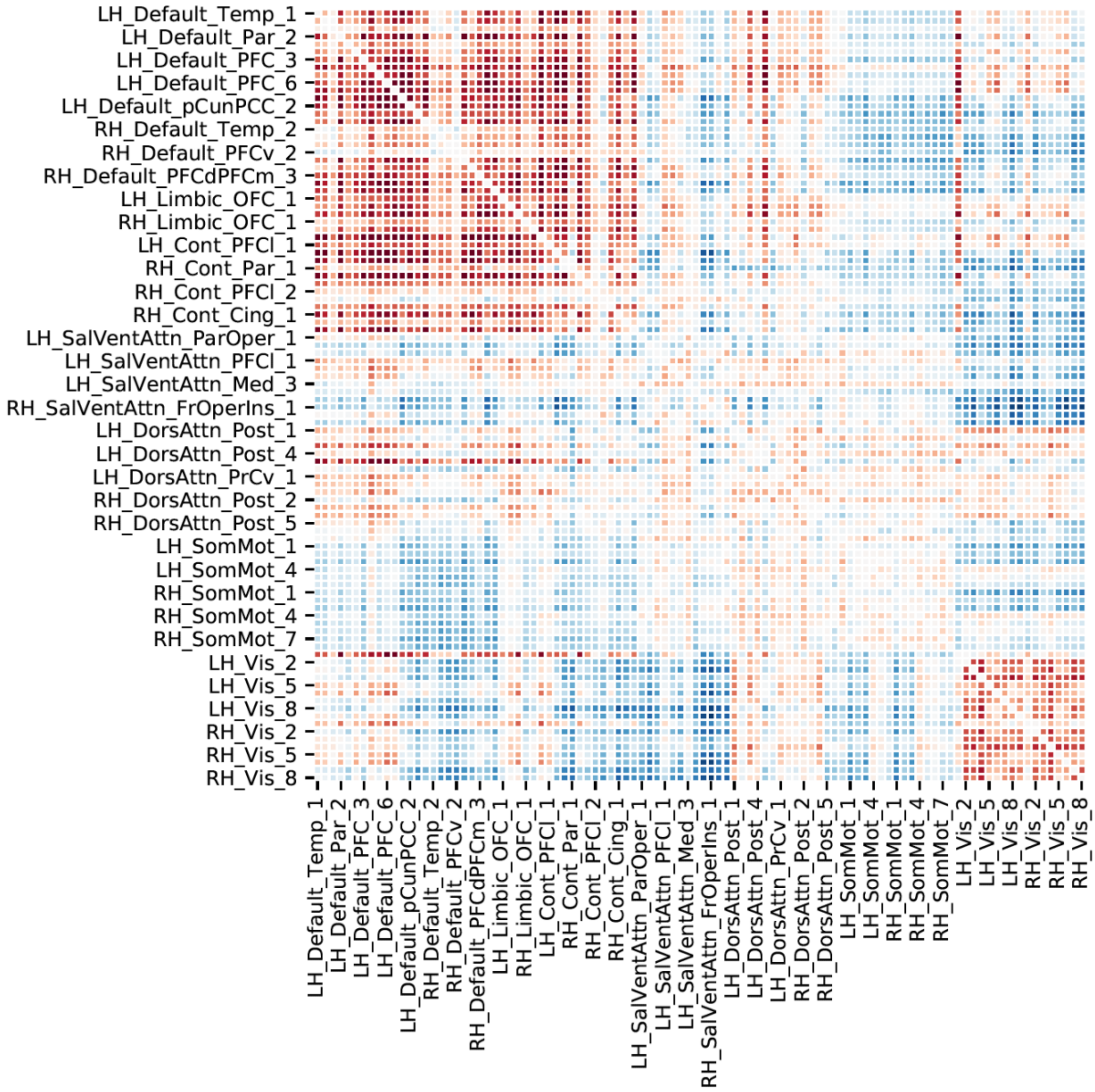

Supplementary Table 12: Sub-analysis accounting for neuroticism:
White matter tract associations with loneliness

	rho_pearson	rho_pearson_pvalues	rho_spearman	rho_spearman_pvalues
Mean FA in fornix on FA skeleton	0.028068347	0.002417562	0.027365614	0.00310148
Mean FA in fornix cres+stria terminalis on FA skeleton (left)	0.019564372	0.034498541	0.019325141	0.036767274
Mean FA in body of corpus callosum on FA skeleton	0.018306343	0.047903307	0.019281496	0.037194605
Mean FA in anterior corona radiata on FA skeleton (left)	0.016044179	0.082964772	0.012057381	0.192613569
Mean FA in fornix cres+stria terminalis on FA skeleton (right)	0.014878803	0.10788082	0.01509402	0.102879678
Mean FA in anterior corona radiata on FA skeleton (right)	0.014339264	0.121265565	0.010905933	0.238614726
Mean FA in external capsule on FA skeleton (right)	0.012846985	0.165071429	0.010211112	0.26986551
Mean FA in tapetum on FA skeleton (right)	0.012145267	0.189390713	0.013630194	0.14079018
Mean FA in genu of corpus callosum on FA skeleton	0.011582212	0.210738479	0.012233945	0.186179239
Mean FA in superior fronto-occipital fasciculus on FA skeleton (left)	0.010374918	0.262256579	0.002370168	0.797870018
Mean FA in external capsule on FA skeleton (left)	0.009203811	0.319968546	0.010371264	0.262424678
Mean FA in posterior thalamic radiation on FA skeleton (right)	0.009027324	0.3293364	0.011807042	0.202014382
Mean FA in cingulum hippocampus on FA skeleton (left)	0.00801326	0.386560074	0.013408461	0.147367587
Mean FA in posterior thalamic radiation on FA skeleton (left)	0.007972555	0.388977036	0.010835054	0.241680419
Mean FA in anterior limb of internal capsule on FA skeleton (right)	0.005856636	0.526842138	0.005627777	0.543118292
Mean FA in tapetum on FA skeleton (left)	0.005400005	0.55956198	0.003619475	0.695725326
Mean FA in superior fronto-occipital fasciculus on FA skeleton (right)	0.005396324	0.559829674	0.000196074	0.983096906
Mean FA in retrolenticular part of internal capsule on FA skeleton (left)	0.004366013	0.637095214	0.0058175	0.529607674
Mean FA in superior corona radiata on FA skeleton (right)	0.003731868	0.686770373	0.004329794	0.639891552
Mean FA in splenium of corpus callosum on FA skeleton	0.003493074	0.705847323	0.009570808	0.30105182
Mean FA in cingulum cingulate gyrus on FA skeleton (right)	0.00286733	0.756694084	0.002068867	0.823109042
Mean FA in superior corona radiata on FA skeleton (left)	0.002747451	0.766564615	0.003371677	0.715617871
Mean FA in anterior limb of internal capsule on FA skeleton (left)	0.002631197	0.776172944	0.003064228	0.740568595
Mean FA in pontine crossing tract on FA skeleton	-0.000486024	0.958117189	0.000949632	0.918271837
Mean FA in uncinate fasciculus on FA skeleton (left)	-0.000511394	0.955933059	-0.000450681	0.961160294
Mean FA in uncinate fasciculus on FA skeleton (right)	-0.000984193	0.915308448	-0.001695924	0.854601701
Mean FA in posterior corona radiata on FA skeleton (right)	-0.001979089	0.830666278	0.002225107	0.809996738
Mean FA in cerebral peduncle on FA skeleton (left)	-0.002685023	0.771719913	0.002837312	0.759162072
Mean FA in posterior corona radiata on FA skeleton (left)	-0.003882143	0.674865616	-0.00343778	0.710291709
Mean FA in inferior cerebellar peduncle on FA skeleton (right)	-0.00451439	0.625693716	-0.009800468	0.289600856
Mean FA in cingulum cingulate gyrus on FA skeleton (left)	-0.004927855	0.594397843	-0.00220524	0.811661152
Mean FA in sagittal stratum on FA skeleton (left)	-0.005012278	0.588096676	-0.003789027	0.682232866
Mean FA in inferior cerebellar peduncle on FA skeleton (left)	-0.005621744	0.543550715	-0.009840404	0.287639939
Mean FA in superior longitudinal fasciculus on FA skeleton (left)	-0.006369232	0.491312317	-0.003834349	0.678643171
Mean FA in cingulum hippocampus on FA skeleton (right)	-0.006940489	0.453284035	-0.003823171	0.679527848
Mean FA in posterior limb of internal capsule on FA skeleton (left)	-0.007908628	0.392791354	-0.007773672	0.400917922
Mean FA in retrolenticular part of internal capsule on FA skeleton (right)	-0.007974663	0.388851619	-0.007640957	0.409007149
Mean FA in posterior limb of internal capsule on FA skeleton (right)	-0.008787723	0.342335787	-0.008556496	0.355187596
Mean FA in middle cerebellar peduncle on FA skeleton	-0.009021369	0.329655563	-0.012316358	0.183230893
Mean FA in sagittal stratum on FA skeleton (right)	-0.009689796	0.29508192	-0.008682046	0.348172055
Mean FA in superior longitudinal fasciculus on FA skeleton (right)	-0.013809519	0.135637759	-0.009005653	0.330498837
Mean FA in cerebral peduncle on FA skeleton (right)	-0.016087246	0.082141887	-0.012938116	0.162093947
Mean FA in medial lemniscus on FA skeleton (left)	-0.016938432	0.067192613	-0.013970568	0.13113567
Mean FA in superior cerebellar peduncle on FA skeleton (left)	-0.017446583	0.059388923	-0.015486626	0.094232795
Mean FA in superior cerebellar peduncle on FA skeleton (right)	-0.022712553	0.014108801	-0.019225402	0.037750019
Mean FA in medial lemniscus on FA skeleton (right)	-0.0253743	0.00610261	-0.024515448	0.008064166
Mean FA in corticospinal tract on FA skeleton (left)	-0.026395829	0.004335614	-0.024408646	0.00834394
Mean FA in corticospinal tract on FA skeleton (right)	-0.040984339	9.41E-06	-0.038970638	2.53E-05

Supplementary Table 13: Sub-analysis accounting for alcohol intake: Network-level posterior parameters

	mean	hpd_5%	hpd_95%
sigma_nets_Cont[0]	0.026	0.001	0.05
sigma_nets_Default[0]	0.068	0.035	0.096
sigma_nets_DorsAttn[0]	0.05	0.008	0.087
sigma_nets_Limbic[0]	0.06	0.002	0.128
sigma_nets_SalVentAttn[0]	0.023	0.002	0.05
sigma_nets_SomMot[0]	0.032	0.002	0.066
sigma_nets_Vis[0]	0.032	0.007	0.061

Supplementary Table 14: Sub-analysis accounting for alcohol intake: Region-level posterior parameters

	mean	hpd_5%	hpd_95%
LH_Cont_pCun_1	-0.085	-0.179	-0.018
LH_Default_Par_1	0.158	0.056	0.301
LH_Default_pCunPCC_1	-0.174	-0.268	-0.063
RH_Default_Temp_1	0.309	0.169	0.445
RH_Default_Temp_3	0.256	0.129	0.416
RH_Default_PFCv_1	0.141	0.017	0.311
LH_DorsAttn_Post_1	-0.159	-0.35	-0.024
LH_DorsAttn_PrCv_1	-0.151	-0.262	-0.038
RH_DorsAttn_Post_1	-0.269	-0.423	-0.15
RH_DorsAttn_Post_4	0.152	0.042	0.247
LH_Limbic_TempPole_1	-0.217	-0.378	-0.057
LH_SalVentAttn_FrOperIns_1	0.138	0.001	0.286
RH_SomMot_2	0.172	0.013	0.363
RH_SomMot_3	-0.159	-0.255	-0.041
RH_SomMot_5	-0.121	-0.216	-0.01
LH_Vis_3	0.153	0.015	0.249
LH_Vis_6	-0.135	-0.232	-0.03

Supplementary Figure 5: Sub-analysis accounting for alcohol intake:
Dominant functional connectivity mode

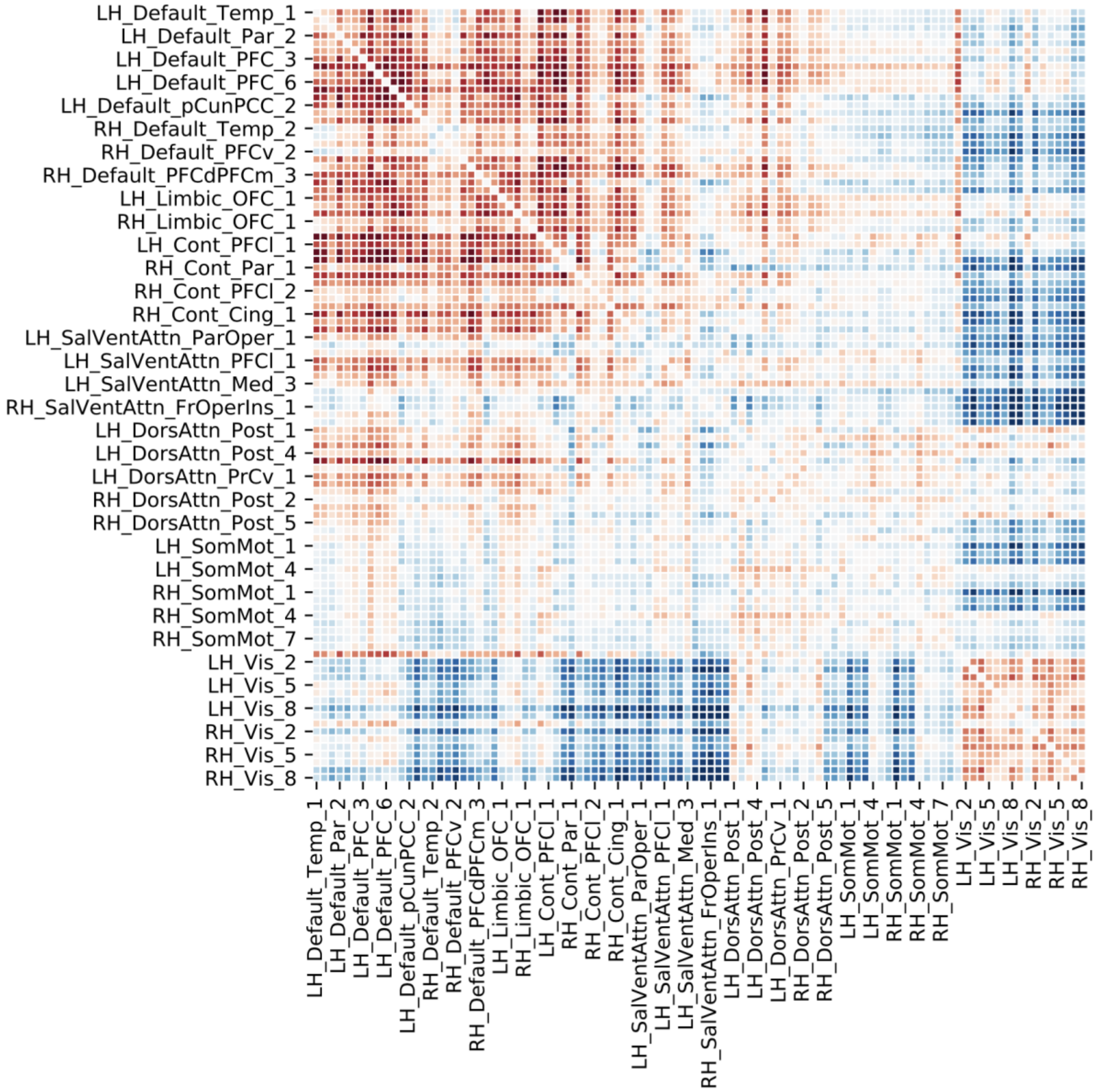

Supplementary Table 15: Sub-analysis accounting for alcohol intake:
White matter tract associations with loneliness

	rho_pearson	rho_pearson_pvalues	rho_spearman	rho_spearman_pvalues
Mean FA in fornix on FA skeleton	0.052607596	1.28E-08	0.053393462	7.77E-09
Mean FA in fornix cres+stria terminalis on FA skeleton (left)	0.039916212	1.60E-05	0.040190018	1.40E-05
Mean FA in fornix cres+stria terminalis on FA skeleton (right)	0.036734747	7.17E-05	0.037464792	5.13E-05
Mean FA in body of corpus callosum on FA skeleton	0.029295741	0.001544558	0.030782055	0.000878215
Mean FA in anterior corona radiata on FA skeleton (left)	0.028876156	0.001803528	0.024754253	0.007468762
Mean FA in external capsule on FA skeleton (right)	0.024774646	0.00741979	0.022596266	0.014609739
Mean FA in anterior corona radiata on FA skeleton (right)	0.022954463	0.01311502	0.019263204	0.037374949
Mean FA in tapetum on FA skeleton (right)	0.022540414	0.01485586	0.024047288	0.00935604
Mean FA in external capsule on FA skeleton (left)	0.018527714	0.045268481	0.020042675	0.030319343
Mean FA in genu of corpus callosum on FA skeleton	0.018503832	0.045546726	0.019343585	0.036587952
Mean FA in superior fronto-occipital fasciculus on FA skeleton (left)	0.018151826	0.04981781	0.010652749	0.249692511
Mean FA in cingulum hippocampus on FA skeleton (left)	0.014429198	0.118948439	0.019813168	0.032267031
Mean FA in posterior thalamic radiation on FA skeleton (right)	0.013491582	0.144874923	0.016403333	0.076302916
Mean FA in posterior thalamic radiation on FA skeleton (left)	0.012989176	0.160443466	0.016215947	0.079722127
Mean FA in superior corona radiata on FA skeleton (right)	0.010414308	0.260449159	0.010934844	0.237372188
Mean FA in superior corona radiata on FA skeleton (left)	0.009910198	0.28423455	0.010501133	0.256495757
Mean FA in tapetum on FA skeleton (left)	0.009832817	0.288011783	0.00803214	0.385442141
Mean FA in superior fronto-occipital fasciculus on FA skeleton (right)	0.009292389	0.315333158	0.004240572	0.64680186
Mean FA in cingulum cingulate gyrus on FA skeleton (right)	0.007929316	0.391554494	0.007151965	0.439639117
Mean FA in uncinate fasciculus on FA skeleton (left)	0.007808617	0.398804034	0.007749131	0.402406464
Mean FA in anterior limb of internal capsule on FA skeleton (right)	0.007450849	0.420762292	0.007315013	0.429281748
Mean FA in cerebral peduncle on FA skeleton (left)	0.005583446	0.546299754	0.011373941	0.219060264
Mean FA in pontine crossing tract on FA skeleton	0.003623288	0.69542088	0.004992098	0.589600087
Mean FA in anterior limb of internal capsule on FA skeleton (left)	0.003047502	0.741934099	0.003488844	0.70618699
Mean FA in splenium of corpus callosum on FA skeleton	0.002981671	0.747316388	0.009036545	0.328842602
Mean FA in inferior cerebellar peduncle on FA skeleton (right)	0.001319505	0.886624418	-0.003201465	0.729395724
Mean FA in inferior cerebellar peduncle on FA skeleton (left)	0.000388876	0.966483293	-0.002821967	0.760424578
Mean FA in cingulum cingulate gyrus on FA skeleton (left)	-0.000132535	0.988573996	0.002502121	0.78688128
Mean FA in posterior corona radiata on FA skeleton (right)	-0.00109617	0.905715283	0.003058791	0.741012354
Mean FA in posterior corona radiata on FA skeleton (left)	-0.001484008	0.872603841	-0.001010756	0.913031573
Mean FA in uncinate fasciculus on FA skeleton (right)	-0.0024833	0.78844612	-0.003178702	0.731245011
Mean FA in sagittal stratum on FA skeleton (left)	-0.002574521	0.780869785	-0.001277049	0.890248816
Mean FA in superior longitudinal fasciculus on FA skeleton (left)	-0.002998508	0.745938613	-0.000422838	0.963558137
Mean FA in cingulum hippocampus on FA skeleton (right)	-0.003395878	0.713666251	-0.000325233	0.971966174
Mean FA in retrolenticular part of internal capsule on FA skeleton (left)	-0.003518858	0.703778295	-0.002322915	0.801815028
Mean FA in sagittal stratum on FA skeleton (right)	-0.007307753	0.429739866	-0.006195372	0.503217179
Mean FA in superior longitudinal fasciculus on FA skeleton (right)	-0.00746482	0.41989168	-0.002606484	0.778219985
Mean FA in middle cerebellar peduncle on FA skeleton	-0.00852159	0.357153813	-0.011859275	0.200025836
Mean FA in posterior limb of internal capsule on FA skeleton (left)	-0.010525959	0.255373061	-0.010473238	0.257761314
Mean FA in medial lemniscus on FA skeleton (left)	-0.013785452	0.136320658	-0.010845724	0.241217153
Mean FA in cerebral peduncle on FA skeleton (right)	-0.014257104	0.123413095	-0.011066593	0.231767685
Mean FA in retrolenticular part of internal capsule on FA skeleton (right)	-0.015194196	0.100615568	-0.014937831	0.106490368
Mean FA in posterior limb of internal capsule on FA skeleton (right)	-0.016492205	0.074723597	-0.016318125	0.077842571
Mean FA in corticospinal tract on FA skeleton (left)	-0.021920946	0.017840423	-0.019946797	0.031120363
Mean FA in medial lemniscus on FA skeleton (right)	-0.024364646	0.008461703	-0.023528623	0.011000101
Mean FA in superior cerebellar peduncle on FA skeleton (left)	-0.030441853	0.001001508	-0.029110367	0.001654432
Mean FA in superior cerebellar peduncle on FA skeleton (right)	-0.039725345	1.75E-05	-0.037247581	5.67E-05
Mean FA in corticospinal tract on FA skeleton (right)	-0.042825412	3.66E-06	-0.040815993	1.02E-05

Supplementary Table 16: Sub-analysis accounting for age completed education: Network-level posterior parameters

	mean	hpd_5%	hpd_95%
sigma_nets_Cont[0]	0.029	0.002	0.059
sigma_nets_Default[0]	0.056	0.023	0.088
sigma_nets_DorsAttn[0]	0.032	0.002	0.062
sigma_nets_Limbic[0]	0.057	0.003	0.126
sigma_nets_SalVentAttn[0]	0.022	0.001	0.049
sigma_nets_SomMot[0]	0.027	0.002	0.06
sigma_nets_Vis[0]	0.046	0.006	0.077

Supplementary Table 17: Sub-analysis accounting for age completed education: Region-level posterior parameters

	mean	hpd_5%	hpd_95%
LH_Cont_pCun_1	-0.11	-0.199	-0.005
LH_Default_Par_1	0.198	0.075	0.309
LH_Default_pCunPCC_1	-0.176	-0.291	-0.065
RH_Default_Temp_1	0.36	0.224	0.55
RH_Default_Temp_3	0.268	0.08	0.43
LH_DorsAttn_Post_1	-0.19	-0.351	-0.033
RH_DorsAttn_Post_1	-0.268	-0.411	-0.117
RH_SomMot_3	-0.134	-0.259	-0.018
RH_SomMot_5	-0.101	-0.207	-0.005
RH_Vis_3	-0.208	-0.354	-0.039

Supplementary Figure 6: Sub-analysis accounting for age completed education:
Dominant functional connectivity mode

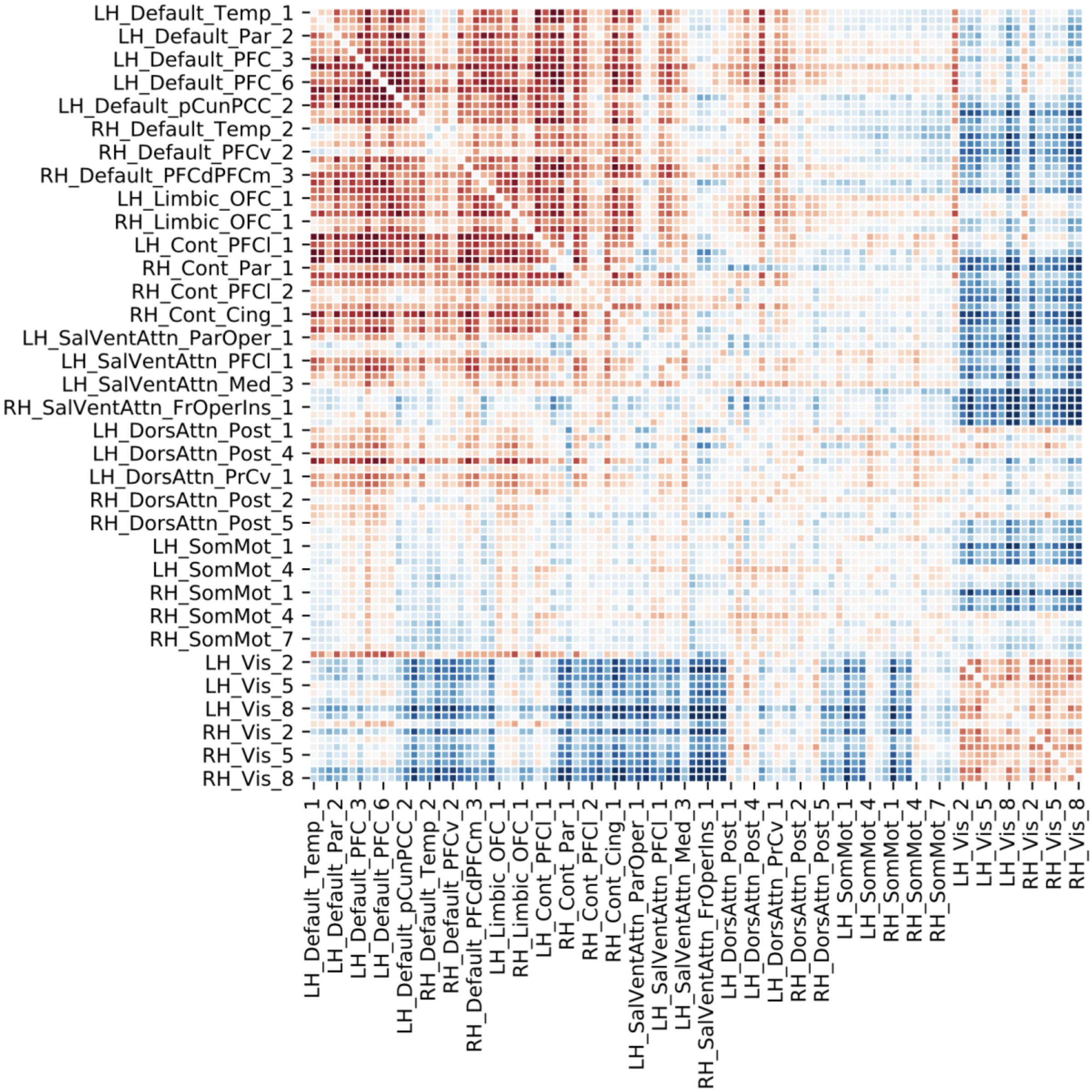

Supplementary Table 18: Sub-analysis accounting for age completed education: White matter tract associations with loneliness

	rho_pearson	rho_pearson_pvalues	rho_spearman	rho_spearman_pvalues
Mean FA in fornix on FA skeleton	0.058602564	2.33E-10	0.059536347	1.20E-10
Mean FA in fornix cres+stria terminalis on FA skeleton (left)	0.046159773	6.03E-07	0.04693388	3.89E-07
Mean FA in fornix cres+stria terminalis on FA skeleton (right)	0.042530683	4.27E-06	0.043886216	2.09E-06
Mean FA in anterior corona radiata on FA skeleton (left)	0.034894743	0.000162179	0.030889619	0.000842261
Mean FA in body of corpus callosum on FA skeleton	0.032305843	0.000480025	0.033965126	0.000241508
Mean FA in anterior corona radiata on FA skeleton (right)	0.028758234	0.00188318	0.025116322	0.00664104
Mean FA in tapetum on FA skeleton (right)	0.027421146	0.003041615	0.028989999	0.001729579
Mean FA in external capsule on FA skeleton (right)	0.027197123	0.003289751	0.025269412	0.006316594
Mean FA in genu of corpus callosum on FA skeleton	0.02259981	0.014594241	0.023875856	0.009873422
Mean FA in superior fronto-occipital fasciculus on FA skeleton (left)	0.022087582	0.016989925	0.014623776	0.114054039
Mean FA in external capsule on FA skeleton (left)	0.019791046	0.03246031	0.021251487	0.02164439
Mean FA in posterior thalamic radiation on FA skeleton (right)	0.01770776	0.055680152	0.020897801	0.023925624
Mean FA in posterior thalamic radiation on FA skeleton (left)	0.016436665	0.075707422	0.019792234	0.032449901
Mean FA in cingulum hippocampus on FA skeleton (left)	0.014358499	0.120767035	0.019735023	0.032954221
Mean FA in superior corona radiata on FA skeleton (right)	0.01359133	0.141926415	0.014040228	0.129224606
Mean FA in superior corona radiata on FA skeleton (left)	0.013066974	0.157953205	0.013600369	0.141661535
Mean FA in superior fronto-occipital fasciculus on FA skeleton (right)	0.012648904	0.171684994	0.007745574	0.402622469
Mean FA in tapetum on FA skeleton (left)	0.012534994	0.175576991	0.010906086	0.238608163
Mean FA in cingulum cingulate gyrus on FA skeleton (right)	0.010824485	0.242139924	0.00991794	0.283858495
Mean FA in anterior limb of internal capsule on FA skeleton (right)	0.009118352	0.324482717	0.009024859	0.329468505
Mean FA in uncinate fasciculus on FA skeleton (left)	0.008351099	0.366855579	0.008326328	0.368278747
Mean FA in cerebral peduncle on FA skeleton (left)	0.008234559	0.373580934	0.014059738	0.128693237
Mean FA in anterior limb of internal capsule on FA skeleton (left)	0.004928661	0.594337501	0.005428502	0.55749155
Mean FA in pontine crossing tract on FA skeleton	0.002930633	0.751497764	0.004401436	0.634365326
Mean FA in cingulum cingulate gyrus on FA skeleton (left)	0.002816074	0.760909598	0.005364126	0.562174072
Mean FA in splenium of corpus callosum on FA skeleton	0.002808022	0.76157254	0.008820299	0.340549363
Mean FA in inferior cerebellar peduncle on FA skeleton (right)	0.002511545	0.786098059	-0.001816191	0.844418085
Mean FA in inferior cerebellar peduncle on FA skeleton (left)	0.000638068	0.94503314	-0.002511114	0.786133852
Mean FA in posterior corona radiata on FA skeleton (right)	0.000207557	0.982107198	0.004513827	0.625736827
Mean FA in posterior corona radiata on FA skeleton (left)	0.00018333	0.984195402	0.000714231	0.938484341
Mean FA in sagittal stratum on FA skeleton (left)	-0.000493189	0.957500343	0.000767835	0.933877707
Mean FA in superior longitudinal fasciculus on FA skeleton (left)	-0.000791127	0.931876705	0.001749427	0.85006825
Mean FA in uncinate fasciculus on FA skeleton (right)	-0.00199161	0.829611291	-0.002765162	0.765103891
Mean FA in retrolenticular part of internal capsule on FA skeleton (left)	-0.002148168	0.816447312	-0.000922906	0.920564221
Mean FA in cingulum hippocampus on FA skeleton (right)	-0.003696126	0.689613444	-0.000544527	0.953081226
Mean FA in sagittal stratum on FA skeleton (right)	-0.005297431	0.567045208	-0.004175739	0.651842465
Mean FA in superior longitudinal fasciculus on FA skeleton (right)	-0.006051742	0.513165792	-0.001240365	0.893382423
Mean FA in middle cerebellar peduncle on FA skeleton	-0.01001664	0.279093752	-0.013335396	0.149585621
Mean FA in posterior limb of internal capsule on FA skeleton (left)	-0.010593097	0.252353978	-0.010705489	0.247355788
Mean FA in cerebral peduncle on FA skeleton (right)	-0.013364764	0.148691054	-0.010146126	0.272925687
Mean FA in medial lemniscus on FA skeleton (left)	-0.014834506	0.10893368	-0.011763244	0.203692897
Mean FA in retrolenticular part of internal capsule on FA skeleton (right)	-0.01607782	0.08232141	-0.015794735	0.087863215
Mean FA in posterior limb of internal capsule on FA skeleton (right)	-0.01696563	0.066754541	-0.016914732	0.067576248
Mean FA in corticospinal tract on FA skeleton (left)	-0.023544078	0.010947622	-0.021521896	0.020030306
Mean FA in medial lemniscus on FA skeleton (right)	-0.026279837	0.004509775	-0.025455832	0.005940809
Mean FA in superior cerebellar peduncle on FA skeleton (left)	-0.031937092	0.000556888	-0.030561693	0.000956356
Mean FA in superior cerebellar peduncle on FA skeleton (right)	-0.041724275	6.47E-06	-0.039233374	2.23E-05
Mean FA in corticospinal tract on FA skeleton (right)	-0.045850521	7.16E-07	-0.04372642	2.28E-06

Supplementary Table 19: Sub-analysis accounting for higher-order age effects (age, age_2, age_3, age_4, age_5): Network-level posterior parameters

	mean	hpd_5%	hpd_95%
sigma_nets_Cont[0]	0.042	0.013	0.076
sigma_nets_Default[0]	0.069	0.034	0.116
sigma_nets_DorsAttn[0]	0.038	0.007	0.066
sigma_nets_Limbic[0]	0.075	0.01	0.139
sigma_nets_SalVentAttn[0]	0.029	0.005	0.047
sigma_nets_SomMot[0]	0.035	0.008	0.061
sigma_nets_Vis[0]	0.048	0.014	0.079

Supplementary Table 20: Sub-analysis accounting for higher-order age effects (age, age_2, age_3, age_4, age_5): Region-level posterior parameters

	mean	hpd_5%	hpd_95%
LH_Cont_pCun_1	-0.079	-0.161	-0.006
RH_Cont_Par_2	-0.068	-0.166	-0.0
RH_Cont_PFCl_2	-0.11	-0.224	-0.023
RH_Cont_PFCmp_1	0.152	0.022	0.25
LH_Default_Temp_2	0.137	0.008	0.288
LH_Default_Par_1	0.168	0.004	0.256
LH_Default_PFC_2	-0.131	-0.297	-0.024
LH_Default_pCunPCC_1	-0.163	-0.282	-0.044
RH_Default_Temp_1	0.307	0.154	0.491
RH_Default_Temp_3	0.271	0.11	0.407
LH_DorsAttn_Post_1	-0.148	-0.287	-0.009
LH_DorsAttn_PrCv_1	-0.139	-0.246	-0.028
RH_DorsAttn_Post_1	-0.285	-0.41	-0.167
RH_DorsAttn_Post_4	0.138	0.024	0.262
LH_Limbic_TempPole_1	-0.181	-0.354	-0.045
LH_SalVentAttn_FrOperIns_1	0.142	0.013	0.243
LH_SalVentAttn_PFCl_1	0.128	0.002	0.266
LH_SalVentAttn_Med_1	-0.131	-0.258	-0.008
RH_SomMot_2	0.134	0.034	0.286
RH_SomMot_3	-0.151	-0.269	-0.031
RH_SomMot_5	-0.113	-0.216	-0.032
RH_Vis_1	-0.194	-0.398	-0.036
RH_Vis_3	-0.155	-0.311	-0.025
RH_Vis_7	0.122	0.013	0.238

Supplementary Figure 7: Sub-analysis accounting for higher-order age effects (age, age$_2$, age$_3$, age$_4$, age$_5$): Dominant functional connectivity mode

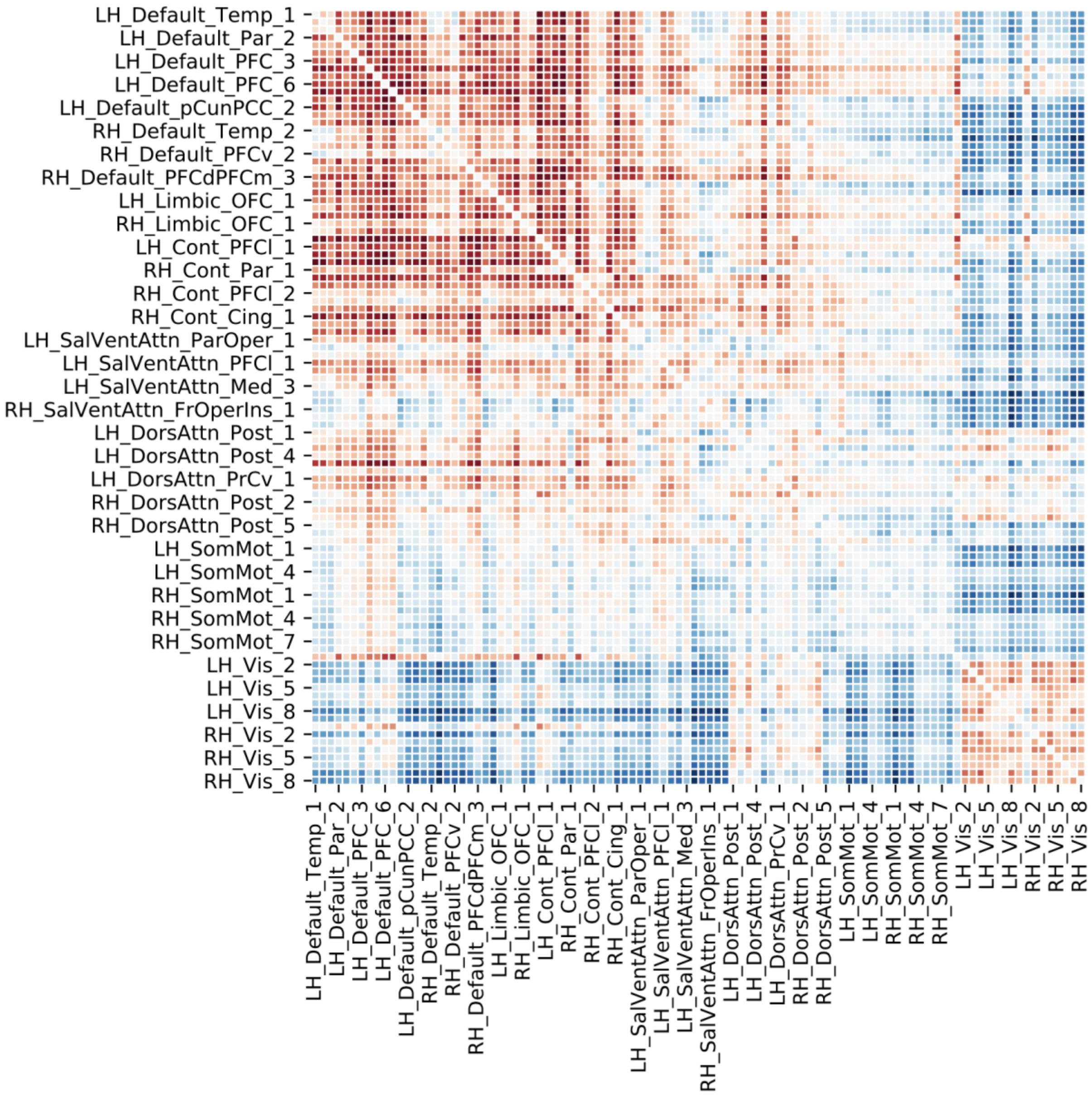

Supplementary Table 21: Sub-analysis accounting for higher-order age effects (age, age_2, age_3, age_4, age_5): White matter tract associations with loneliness

	rho_pearson	rho_pearson_pvalues	rho_spearman	rho_spearman_pvalues
Mean FA in fornix on FA skeleton	0.011927271	0.197458597	0.010665553	0.249123801
Mean FA in body of corpus callosum on FA skeleton	0.010080077	0.276060157	0.012622435	0.172583548
Mean FA in cingulum hippocampus on FA skeleton (left)	0.008875758	0.337521877	0.014260487	0.123324078
Mean FA in fornix cres+stria terminalis on FA skeleton (left)	0.006299376	0.496077375	0.008330037	0.368065434
Mean FA in fornix cres+stria terminalis on FA skeleton (right)	0.001375963	0.881808235	0.005057896	0.58470475
Mean FA in external capsule on FA skeleton (right)	0.001067337	0.908184203	-0.002476665	0.788997944
Mean FA in tapetum on FA skeleton (right)	-0.000598962	0.948397119	0.001426684	0.877485257
Mean FA in uncinate fasciculus on FA skeleton (left)	-0.002634612	0.775890186	-0.003012605	0.744785697
Mean FA in external capsule on FA skeleton (left)	-0.002991866	0.746482085	-0.002655769	0.77413918
Mean FA in splenium of corpus callosum on FA skeleton	-0.003048326	0.741866776	0.003017076	0.744420132
Mean FA in pontine crossing tract on FA skeleton	-0.003407322	0.712744114	-0.001774527	0.847943143
Mean FA in genu of corpus callosum on FA skeleton	-0.003422648	0.711509733	-0.001768175	0.848480769
Mean FA in cingulum hippocampus on FA skeleton (right)	-0.004643534	0.61584231	-0.001561837	0.865984096
Mean FA in cingulum cingulate gyrus on FA skeleton (right)	-0.005080992	0.582990923	-0.005506241	0.551862469
Mean FA in tapetum on FA skeleton (left)	-0.005458222	0.555336176	-0.006312945	0.495149857
Mean FA in superior corona radiata on FA skeleton (right)	-0.005516059	0.551153488	-0.004301036	0.642115535
Mean FA in anterior corona radiata on FA skeleton (left)	-0.005658374	0.540927848	-0.010002233	0.279785818
Mean FA in anterior corona radiata on FA skeleton (right)	-0.006270759	0.498036511	-0.008073144	0.383021049
Mean FA in anterior limb of internal capsule on FA skeleton (right)	-0.006751919	0.465650057	-0.006398051	0.489353673
Mean FA in superior corona radiata on FA skeleton (left)	-0.00697832	0.450825716	-0.006092228	0.510351144
Mean FA in uncinate fasciculus on FA skeleton (right)	-0.008582001	0.353755214	-0.009063677	0.327392393
Mean FA in anterior limb of internal capsule on FA skeleton (left)	-0.009734528	0.292858224	-0.009710535	0.294049551
Mean FA in superior fronto-occipital fasciculus on FA skeleton (left)	-0.009956477	0.281991597	-0.01383704	0.134860064
Mean FA in retrolenticular part of internal capsule on FA skeleton (left)	-0.011135399	0.228878407	-0.00999136	0.280308924
Mean FA in cingulum cingulate gyrus on FA skeleton (left)	-0.012163827	0.1887152	-0.008919835	0.335128066
Mean FA in inferior cerebellar peduncle on FA skeleton (right)	-0.012760218	0.167944439	-0.018956975	0.040506493
Mean FA in posterior corona radiata on FA skeleton (right)	-0.012806468	0.166408394	-0.008659893	0.349403484
Mean FA in posterior limb of internal capsule on FA skeleton (left)	-0.013663969	0.139808395	-0.013437829	0.146483171
Mean FA in inferior cerebellar peduncle on FA skeleton (left)	-0.013696233	0.138875496	-0.019151728	0.038490208
Mean FA in cerebral peduncle on FA skeleton (left)	-0.015297701	0.098318095	-0.010158117	0.272359247
Mean FA in posterior corona radiata on FA skeleton (left)	-0.015471087	0.094563588	-0.014558892	0.11566819
Mean FA in superior fronto-occipital fasciculus on FA skeleton (right)	-0.016298278	0.07820481	-0.020066911	0.030119686
Mean FA in posterior thalamic radiation on FA skeleton (right)	-0.017385794	0.060280939	-0.013890085	0.133370841
Mean FA in posterior thalamic radiation on FA skeleton (left)	-0.017632595	0.056727198	-0.012691435	0.170248511
Mean FA in retrolenticular part of internal capsule on FA skeleton (right)	-0.018062916	0.050948224	-0.017919035	0.052823004
Mean FA in superior cerebellar peduncle on FA skeleton (left)	-0.018285069	0.048163171	-0.016288503	0.078383713
Mean FA in posterior limb of internal capsule on FA skeleton (right)	-0.019157536	0.038431413	-0.019084558	0.03917577
Mean FA in superior longitudinal fasciculus on FA skeleton (left)	-0.019270636	0.037301588	-0.016140546	0.081132648
Mean FA in sagittal stratum on FA skeleton (left)	-0.01977251	0.032623016	-0.018029597	0.05137734
Mean FA in middle cerebellar peduncle on FA skeleton	-0.020040092	0.030340687	-0.023119402	0.012473424
Mean FA in medial lemniscus on FA skeleton (left)	-0.020131194	0.029595595	-0.017220606	0.062761118
Mean FA in superior longitudinal fasciculus on FA skeleton (right)	-0.021145784	0.022305457	-0.015969622	0.084405135
Mean FA in superior cerebellar peduncle on FA skeleton (right)	-0.026512222	0.004167002	-0.022315848	0.015882711
Mean FA in sagittal stratum on FA skeleton (right)	-0.026690999	0.003919579	-0.025162102	0.006542483
Mean FA in cerebral peduncle on FA skeleton (right)	-0.02777598	0.002683325	-0.025044732	0.006797844
Mean FA in medial lemniscus on FA skeleton (right)	-0.030112531	0.001135945	-0.029430671	0.001468853
Mean FA in corticospinal tract on FA skeleton (left)	-0.030182642	0.001105999	-0.027847798	0.002615678
Mean FA in corticospinal tract on FA skeleton (right)	-0.048204136	1.87E-07	-0.046100399	6.23E-07

Supplementary Table 22: Sub-analysis accounting for adiposity (waist circumference): Network-level posterior parameters

	mean	hpd_5%	hpd_95%
sigma_nets_Cont[0]	0.03	0.002	0.057
sigma_nets_Default[0]	0.067	0.029	0.099
sigma_nets_DorsAttn[0]	0.038	0.002	0.072
sigma_nets_Limbic[0]	0.072	0.003	0.147
sigma_nets_SalVentAttn[0]	0.026	0.002	0.051
sigma_nets_SomMot[0]	0.029	0.002	0.062
sigma_nets_Vis[0]	0.049	0.019	0.085

Supplementary Table 23: Sub-analysis accounting for adiposity (waist circumference): Region-level posterior parameters

	mean	hpd_5%	hpd_95%
LH_Cont_pCun_1	-0.098	-0.193	-0.009
LH_Default_Par_1	0.202	0.083	0.323
LH_Default_PFC_3	-0.184	-0.368	-0.002
LH_Default_pCunPCC_1	-0.176	-0.284	-0.063
RH_Default_Temp_1	0.349	0.202	0.495
RH_Default_Temp_3	0.277	0.106	0.45
LH_DorsAttn_Post_1	-0.206	-0.373	-0.043
RH_DorsAttn_Post_1	-0.279	-0.416	-0.132
RH_DorsAttn_Post_4	0.113	0.004	0.233
RH_SomMot_3	-0.142	-0.263	-0.023
RH_Vis_1	-0.186	-0.354	-0.008
RH_Vis_3	-0.184	-0.336	-0.033
RH_Vis_7	0.14	0.007	0.276

Supplementary Figure 8: Sub-analysis accounting for adiposity (waist circumference): Dominant functional connectivity mode

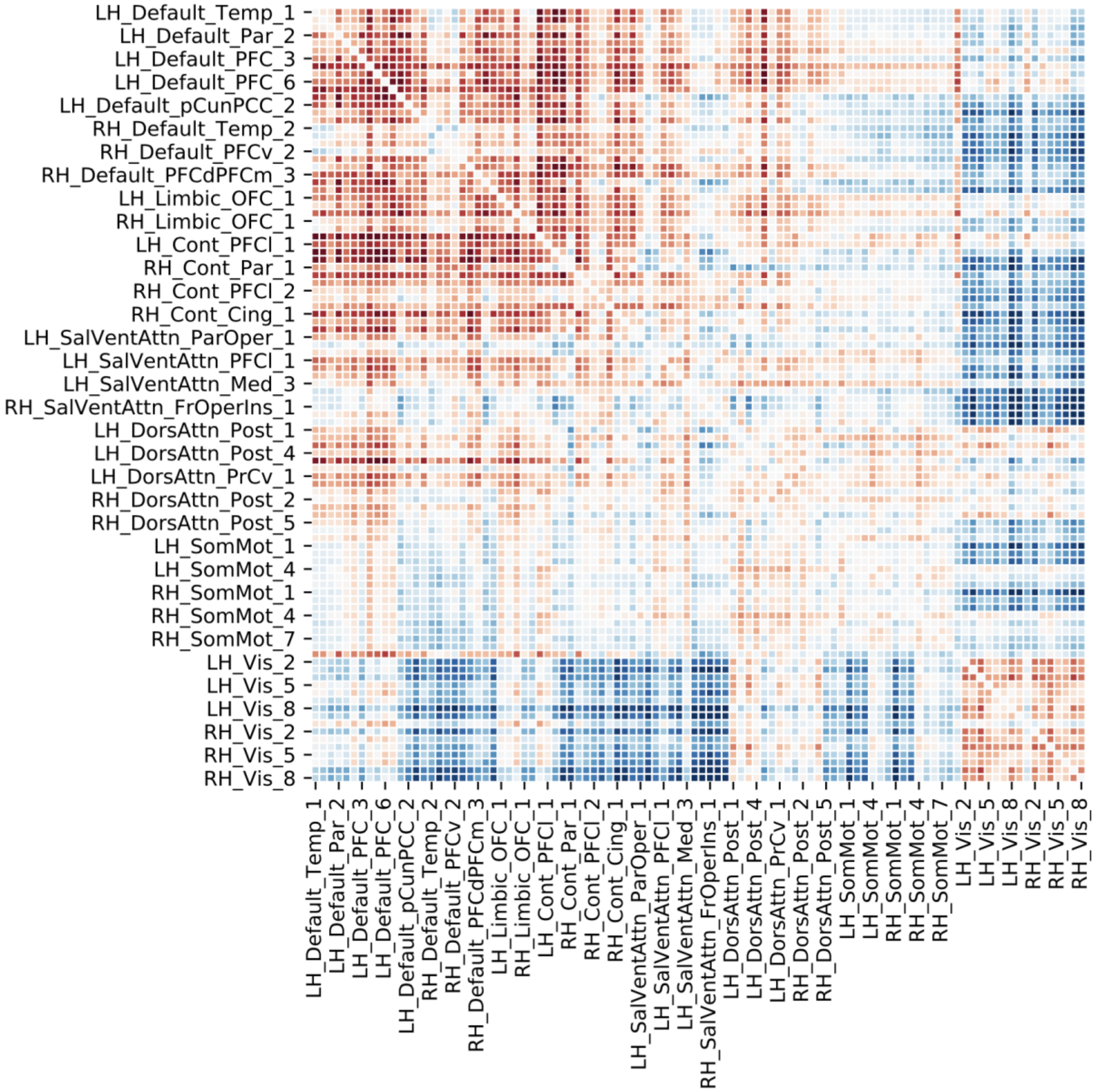

Supplementary Table 24: Sub-analysis accounting for adiposity (waist circumference): White matter tract associations with loneliness

	rho_pearson	rho_pearson_pvalues	rho_spearman	rho_spearman_pvalues
Mean FA in fornix on FA skeleton	0.050495584	4.77E-08	0.051539113	2.51E-08
Mean FA in fornix cres+stria terminalis on FA skeleton (left)	0.037371771	5.36E-05	0.038559537	3.07E-05
Mean FA in fornix cres+stria terminalis on FA skeleton (right)	0.034985094	0.000155943	0.036432798	8.22E-05
Mean FA in anterior corona radiata on FA skeleton (left)	0.02920112	0.001599772	0.025135427	0.00659975
Mean FA in body of corpus callosum on FA skeleton	0.028556531	0.002026951	0.030415412	0.001011732
Mean FA in anterior corona radiata on FA skeleton (right)	0.023337214	0.011668812	0.020083983	0.029979727
Mean FA in external capsule on FA skeleton (right)	0.022974548	0.01303536	0.02129117	0.021400655
Mean FA in tapetum on FA skeleton (right)	0.021753399	0.018732941	0.023177833	0.012252892
Mean FA in genu of corpus callosum on FA skeleton	0.019658086	0.033642986	0.021079852	0.022726627
Mean FA in superior fronto-occipital fasciculus on FA skeleton (left)	0.017703014	0.055745784	0.010502149	0.256449735
Mean FA in external capsule on FA skeleton (left)	0.016312827	0.077939132	0.018099918	0.050475198
Mean FA in cingulum hippocampus on FA skeleton (left)	0.015886576	0.086033243	0.021206826	0.021921595
Mean FA in posterior thalamic radiation on FA skeleton (right)	0.011554958	0.211814276	0.015707393	0.089632538
Mean FA in cingulum cingulate gyrus on FA skeleton (right)	0.011289081	0.222517751	0.010391232	0.261506966
Mean FA in posterior thalamic radiation on FA skeleton (left)	0.009944647	0.28256381	0.014078538	0.128182848
Mean FA in superior corona radiata on FA skeleton (right)	0.009587678	0.300200585	0.010226202	0.269158278
Mean FA in tapetum on FA skeleton (left)	0.009469065	0.306219948	0.007651982	0.408331435
Mean FA in superior corona radiata on FA skeleton (left)	0.009272287	0.316381245	0.01016142	0.272203378
Mean FA in anterior limb of internal capsule on FA skeleton (right)	0.009237321	0.318209724	0.009120127	0.324388522
Mean FA in superior fronto-occipital fasciculus on FA skeleton (right)	0.008455559	0.360891947	0.003301661	0.72127439
Mean FA in uncinate fasciculus on FA skeleton (left)	0.00755744	0.414147065	0.007519319	0.416505865
Mean FA in cerebral peduncle on FA skeleton (left)	0.006902075	0.455787991	0.012769682	0.167629266
Mean FA in anterior limb of internal capsule on FA skeleton (left)	0.005792656	0.531367114	0.00620594	0.502489198
Mean FA in cingulum cingulate gyrus on FA skeleton (left)	0.004008883	0.66488801	0.006541995	0.479634063
Mean FA in pontine crossing tract on FA skeleton	0.001557531	0.866350106	0.002835686	0.759295817
Mean FA in splenium of corpus callosum on FA skeleton	0.001262742	0.891470767	0.007523078	0.416272868
Mean FA in inferior cerebellar peduncle on FA skeleton (right)	0.001170171	0.899382969	-0.003281744	0.722886307
Mean FA in inferior cerebellar peduncle on FA skeleton (left)	0.000143423	0.987635466	-0.003144507	0.734026002
Mean FA in posterior corona radiata on FA skeleton (right)	-0.000921168	0.920713304	0.003453633	0.709016502
Mean FA in cingulum hippocampus on FA skeleton (right)	-0.0010386	0.910645706	0.002064396	0.823485035
Mean FA in posterior corona radiata on FA skeleton (left)	-0.001370314	0.882289931	-0.00081392	0.929919075
Mean FA in uncinate fasciculus on FA skeleton (right)	-0.001487874	0.87227482	-0.002218857	0.810520233
Mean FA in superior longitudinal fasciculus on FA skeleton (left)	-0.004187812	0.65090265	-0.001237418	0.893634226
Mean FA in sagittal stratum on FA skeleton (left)	-0.004908535	0.595844155	-0.003070325	0.740071023
Mean FA in retrolenticular part of internal capsule on FA skeleton (left)	-0.005661595	0.540697542	-0.004053491	0.661390178
Mean FA in superior longitudinal fasciculus on FA skeleton (right)	-0.008269649	0.371547991	-0.003265281	0.724219534
Mean FA in sagittal stratum on FA skeleton (right)	-0.008457727	0.360768831	-0.00702367	0.447888674
Mean FA in middle cerebellar peduncle on FA skeleton	-0.008647864	0.350073296	-0.011953831	0.19646237
Mean FA in posterior limb of internal capsule on FA skeleton (left)	-0.010086226	0.27576735	-0.009990953	0.280328492
Mean FA in retrolenticular part of internal capsule on FA skeleton (right)	-0.015248048	0.099414947	-0.015141527	0.101800958
Mean FA in corticospinal tract on FA skeleton (left)	-0.020392331	0.02754641	-0.018715324	0.043132125
Mean FA in medial lemniscus on FA skeleton (right)	-0.022191531	0.016477584	-0.02084689	0.024270591
Mean FA in superior cerebellar peduncle on FA skeleton (left)	-0.029967795	0.001200151	-0.028571886	0.002015662
Mean FA in superior cerebellar peduncle on FA skeleton (right)	-0.039364988	2.09E-05	-0.036790396	6.99E-05
Mean FA in corticospinal tract on FA skeleton (right)	-0.040495032	1.20E-05	-0.039037097	2.45E-05

Supplementary Table 25: Sub-analysis accounting for non-white-British ancestry: Network-level posterior parameters

	mean	hpd_5%	hpd_95%
sigma_nets_Cont[0]	0.03	0.001	0.058
sigma_nets_Default[0]	0.063	0.026	0.098
sigma_nets_DorsAttn[0]	0.045	0.007	0.077
sigma_nets_Limbic[0]	0.08	0.002	0.168
sigma_nets_SalVentAttn[0]	0.026	0.002	0.05
sigma_nets_SomMot[0]	0.03	0.001	0.062
sigma_nets_Vis[0]	0.039	0.005	0.072

Supplementary Table 26: Sub-analysis accounting for non-white-British ancestry: Region-level posterior parameters

	mean	hpd_5%	hpd_95%
LH_Cont_pCun_1	-0.105	-0.216	-0.03
LH_Default_Par_1	0.199	0.085	0.314
LH_Default_PFC_3	-0.178	-0.443	-0.026
LH_Default_pCunPCC_1	-0.188	-0.295	-0.085
RH_Default_Temp_1	0.345	0.2	0.509
RH_Default_Temp_3	0.257	0.133	0.423
LH_DorsAttn_Post_1	-0.186	-0.339	-0.029
RH_DorsAttn_Post_1	-0.269	-0.402	-0.125
RH_DorsAttn_Post_4	0.117	0.004	0.227
RH_Vis_3	-0.197	-0.381	-0.06
RH_Vis_7	0.136	0.01	0.263

Supplementary Figure 9: Sub-analysis accounting for non-white-British ancestry: Dominant functional connectivity mode

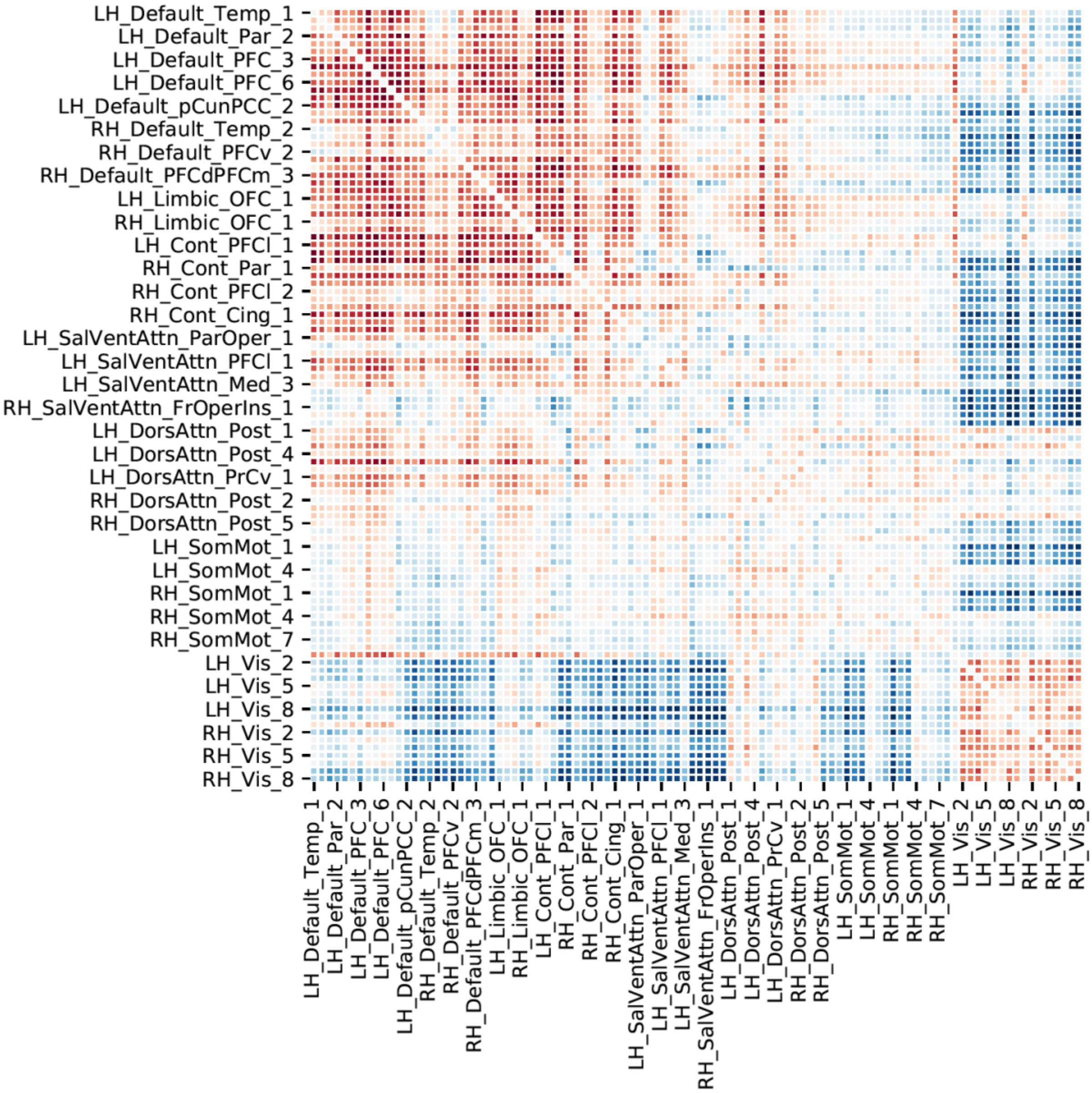

Supplementary Table 27: Sub-analysis accounting for non-white-British ancestry: White matter tract associations with loneliness

	rho_pearson	rho_pearson_pvalues	rho_spearman	rho_spearman_pvalues
Mean FA in body of corpus callosum on FA skeleton	0.032505783	0.008454387	0.032576339	0.008313083
Mean FA in fornix on FA skeleton	0.02693474	0.029119841	0.027200081	0.02756922
Mean FA in fornix cres+stria terminalis on FA skeleton (right)	0.022133879	0.0729955	0.023343902	0.058638018
Mean FA in cingulum hippocampus on FA skeleton (left)	0.020289901	0.100287123	0.027718928	0.024741959
Mean FA in genu of corpus callosum on FA skeleton	0.011956082	0.332861351	0.011684535	0.34395916
Mean FA in cingulum cingulate gyrus on FA skeleton (right)	0.011618417	0.346696704	0.013183802	0.285605204
Mean FA in pontine crossing tract on FA skeleton	0.011430749	0.3545421	0.013827036	0.262749809
Mean FA in tapetum on FA skeleton (right)	0.010387813	0.400157832	0.013820583	0.262972652
Mean FA in cingulum cingulate gyrus on FA skeleton (left)	0.009311285	0.450762613	0.009986345	0.418617745
Mean FA in anterior corona radiata on FA skeleton (left)	0.009144099	0.458934309	0.003234241	0.793364442
Mean FA in superior fronto-occipital fasciculus on FA skeleton (left)	0.009137441	0.459261441	0.005841703	0.636121948
Mean FA in posterior thalamic radiation on FA skeleton (left)	0.008303526	0.501252492	0.013225587	0.284080894
Mean FA in anterior corona radiata on FA skeleton (right)	0.008202578	0.50647012	0.004061717	0.742183786
Mean FA in inferior cerebellar peduncle on FA skeleton (right)	0.00812455	0.5105226	0.005686823	0.645097269
Mean FA in posterior corona radiata on FA skeleton (right)	0.008045281	0.514656788	0.009424423	0.445279786
Mean FA in superior corona radiata on FA skeleton (left)	0.007833616	0.525780617	0.008062934	0.5137346
Mean FA in tapetum on FA skeleton (left)	0.006509596	0.59803903	0.003147444	0.798789206
Mean FA in splenium of corpus callosum on FA skeleton	0.006117713	0.620259378	0.013283989	0.281959633
Mean FA in superior corona radiata on FA skeleton (right)	0.005371076	0.663554737	0.007041213	0.568487721
Mean FA in fornix cres+stria terminalis on FA skeleton (left)	0.004990424	0.686079863	0.004908876	0.690942826
Mean FA in inferior cerebellar peduncle on FA skeleton (left)	0.004922794	0.690111958	0.006437021	0.602126792
Mean FA in posterior corona radiata on FA skeleton (left)	0.004807045	0.697033234	0.007224487	0.558464209
Mean FA in medial lemniscus on FA skeleton (left)	0.004586031	0.710318801	0.007511982	0.542915847
Mean FA in external capsule on FA skeleton (right)	0.004288278	0.728356658	0.00492388	0.69004713
Mean FA in anterior limb of internal capsule on FA skeleton (right)	0.003545867	0.773971993	0.004348244	0.724711384
Mean FA in cingulum hippocampus on FA skeleton (right)	0.002995289	0.80832206	0.006959901	0.572962144
Mean FA in corticospinal tract on FA skeleton (left)	0.002632592	0.831158524	0.004547813	0.712625209
Mean FA in medial lemniscus on FA skeleton (right)	0.002448078	0.842832312	0.003005148	0.807703521
Mean FA in anterior limb of internal capsule on FA skeleton (left)	0.002206001	0.858200055	0.005186046	0.674467535
Mean FA in external capsule on FA skeleton (left)	0.001850221	0.880882257	0.004594438	0.709811755
Mean FA in superior longitudinal fasciculus on FA skeleton (left)	0.001078402	0.930400753	0.006948931	0.573567113
Mean FA in superior fronto-occipital fasciculus on FA skeleton (right)	-0.000644908	0.958344152	-0.00453539	0.713375477
Mean FA in posterior thalamic radiation on FA skeleton (right)	-0.000971119	0.937309668	0.007713326	0.532156641
Mean FA in superior longitudinal fasciculus on FA skeleton (right)	-0.005654475	0.646978476	0.000274911	0.982236355
Mean FA in retrolenticular part of internal capsule on FA skeleton (left)	-0.006767812	0.583598269	-0.003142926	0.799071804
Mean FA in uncinate fasciculus on FA skeleton (right)	-0.008218935	0.505622749	-0.006150171	0.618405353
Mean FA in uncinate fasciculus on FA skeleton (left)	-0.009173874	0.457472922	-0.004385189	0.722468642
Mean FA in posterior limb of internal capsule on FA skeleton (right)	-0.010549736	0.392853095	-0.01098548	0.373600689
Mean FA in cerebral peduncle on FA skeleton (left)	-0.010625742	0.389452342	-0.000895559	0.942178545
Mean FA in posterior limb of internal capsule on FA skeleton (left)	-0.012649542	0.305581814	-0.014976049	0.225133909
Mean FA in sagittal stratum on FA skeleton (left)	-0.013443565	0.276218516	-0.012297277	0.31924856
Mean FA in middle cerebellar peduncle on FA skeleton	-0.013735786	0.265912932	-0.009390059	0.44694109
Mean FA in retrolenticular part of internal capsule on FA skeleton (right)	-0.01475052	0.232196235	-0.013599234	0.270695219
Mean FA in superior cerebellar peduncle on FA skeleton (right)	-0.015792401	0.200855499	-0.007100422	0.565240104
Mean FA in sagittal stratum on FA skeleton (right)	-0.020273906	0.100555311	-0.016653588	0.177375518
Mean FA in corticospinal tract on FA skeleton (right)	-0.020453007	0.097584703	-0.017349256	0.159951461
Mean FA in cerebral peduncle on FA skeleton (right)	-0.022358196	0.070135707	-0.018637176	0.13115323

Supplementary Figure 10:

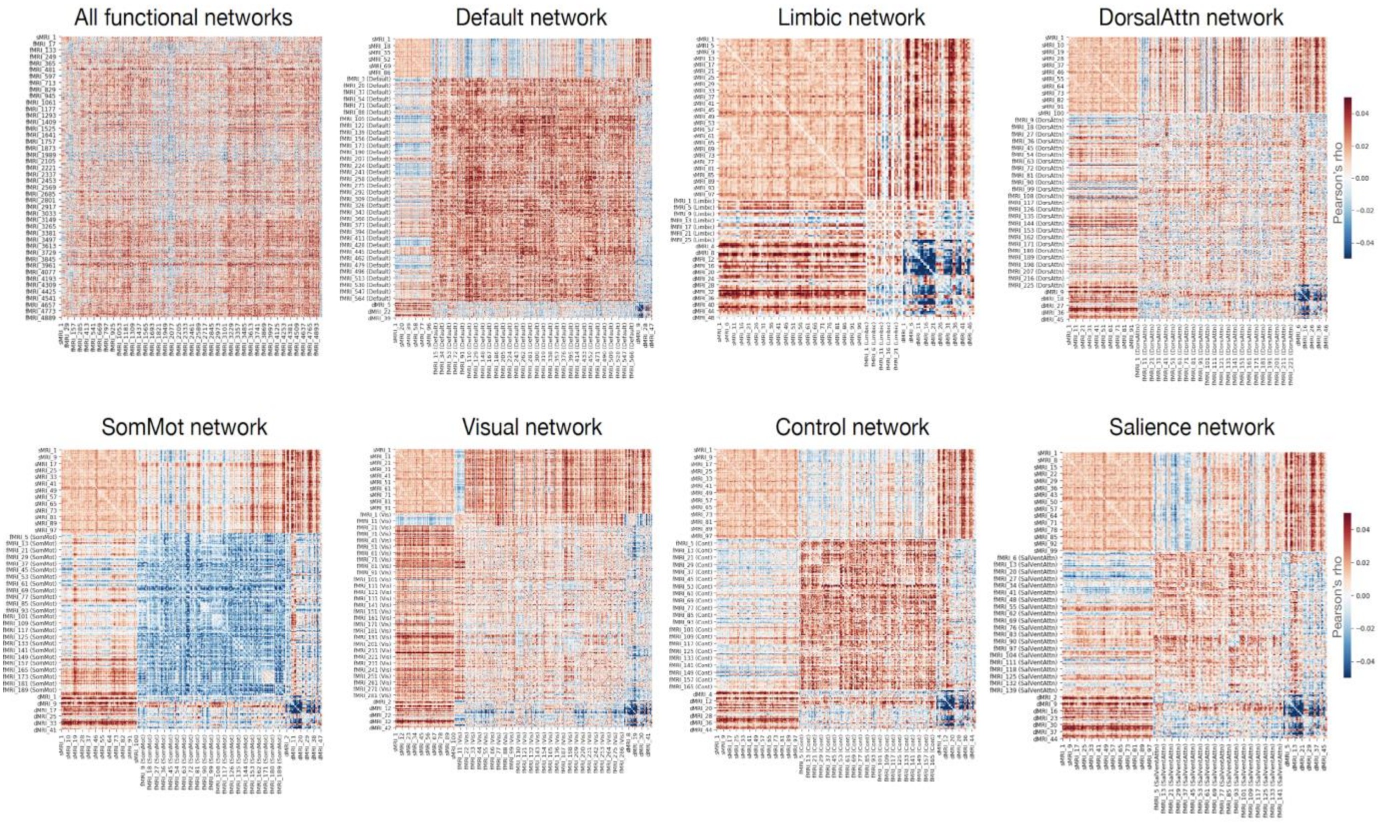

Across-modality relationships between brain measures and trait loneliness. Structural brain features (sMRI; *upper rows of each matrix*), functional brain features (fMRI; *middle rows of each matrix*), and diffusion brain features (dMRI; *lower rows of each matrix*) are shown with their extent of mutual linear associations (Pearson's correlation coefficient). We contrasted effect sizes (Pearson's rho) from lonely and non-lonely UK Biobank participants for the purpose of visualization. *Upper left panel*: linear associations between the original measures of 100 sMRI region volumes, 4950 fMRI connectivity strengths, and 48 dMRI fiber strengths in lonely versus non-lonely participants. *Other panels:* Shows the differential linear associations involving the same 100 sMRI and 48 dMRI features but subsetting the 4950 fMRI functional coupling links to the fMRI features that belong to one of the seven large-scale brain networks (default network, limbic network, etc.) to improve readability.

Supplementary Figure 11:

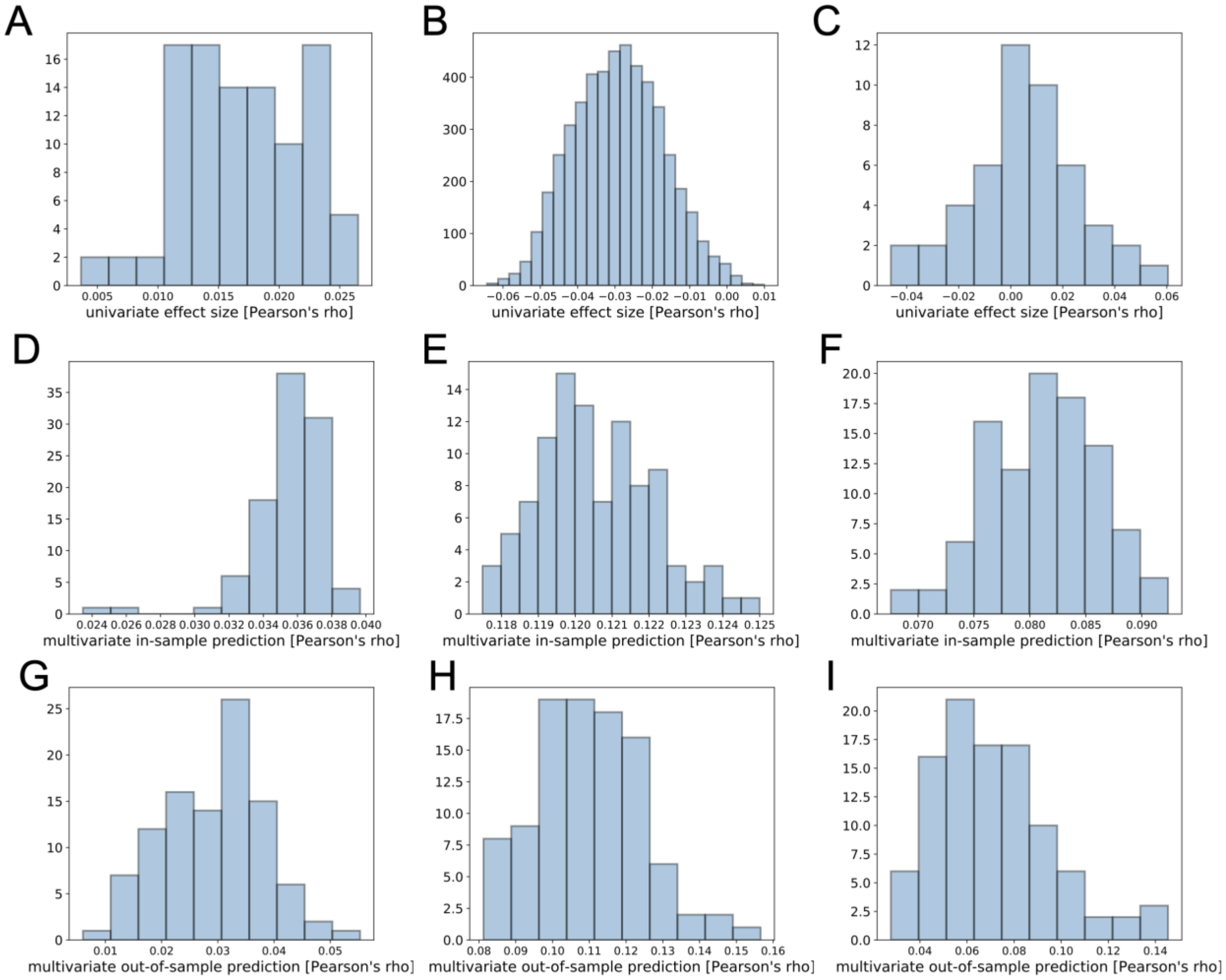

Effect sizes, in-sample accuracy and out-of-sample prediction compared across structural, functional and diffusion MRI. We computed additional characteristics based on the original 100 brain region volumes (*first column*), 4950 functional connectivity links (*second column)*, and 48 fiber tract microstructure measurements (*third column). A-C*) Each individual brain feature (i.e., specific region volume, connectivity link, or fiber microstructure, respectively) was assessed for its univariate association with trait loneliness using Pearson's correlation coefficient. Using one hundred iterations of Monte-Carlo cross-validation with 10 folds, multivariate partial least squares was applied to all features from a given brain-imaging modality to derive the dominant brain-behavior mode and compute *D-F)* the in-sample model performance on the training participants and *G-I)* out-of-sample prediction accuracy on the unseen testing participants. All panels show histograms pitting value range bins (*x axis)* against their occurrence (*y axis*).
